# The Missing Link in Ferrocenophane Chemistry: Isolation of a Carba[1]Ferrocenophane

**DOI:** 10.1002/anie.2211037

**Published:** 2026-07-12

**Authors:** Aylin Feuerstein, Sergi Danés, Kathrin Kolling, Bernd Morgenstern, Markus Gallei, André Schäfer

**Affiliations:** ^1^ Department of Chemistry Faculty of Natural Sciences and Technology Campus Saarbrücken Saarland University Saarbrücken Germany; ^2^ Institute of Chemical Research of Catalonia (ICIQ) Barcelona Institute of Science and Technology (BIST) Tarragona Spain; ^3^ Institute of Computational Chemistry and Catalysis (IQCC) University of Girona Girona Catalonia Spain; ^4^ Saarland Center for Energy Materials and Sustainability – Saarene Saarland University Saarbrücken Germany

**Keywords:** ferrocene, ferrocenophane, iron, metallocene, metallocenophane

## Abstract

[1]Ferrocenophanes, which are single‐atom bridged *ansa*‐ferrocenes, have gained significant interest as monomers for ring‐opening polymerization (ROP) reactions for the preparation of main‐chain ferrocene‐based metallopolymers. While all conceivable third and fourth row *p*‐block elements are known as bridging atoms, boron remained the only second row *p*‐block element to be incorporated into [1]ferrocenophanes, so far. Here, we describe the synthesis, isolation and full characterization of a carba[1]ferrocenophane, previously speculated to be unstable due to its high energy conformation. Single‐crystal X‐ray diffraction reveals the highest angular deflection from ferrocene's coplanarity so far (*α* = 36.7°, *δ* = 151.8°), which is reflected by its deep red color (*λ*_max_ = 511 nm) and high air‐sensitivity. Nonetheless, the carba[1]ferrocenophane is thermally robust up to 200°C, which, according to DFT calculations, is due to non‐covalent interactions arising from the ligand system.

## Graphical abstract

The synthesis and isolation of a crystalline carba[1]ferrocenophane is reported. A single carbon atom is the smallest bridging motif yet incorporated into a ferrocenophane, resulting in a dihedral angle between the cyclopentadienyl ring planes of 36.7°, which is the highest angular deflection from ferrocene's coplanarity observed so far. Although this bending indicates substantial ring‐strain, the compound exhibits high thermal stability.

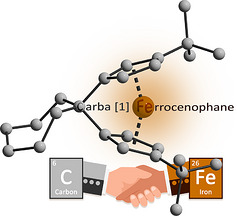

## 1 Introduction

**FIGURE 1:**
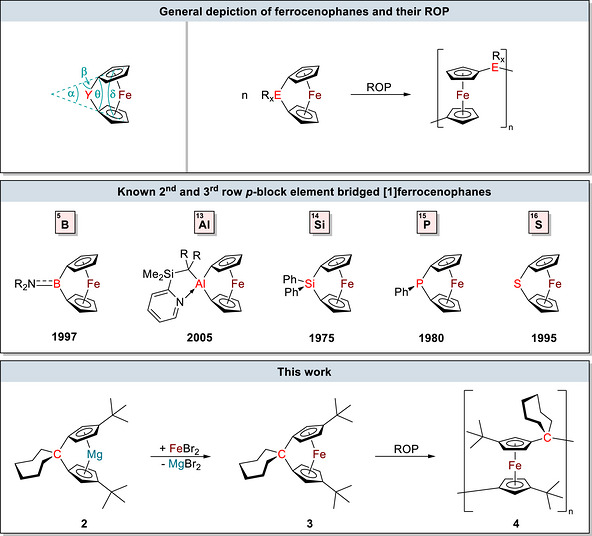
Top: General depiction of ferrocenophanes and their ROP; center: selected examples of second and third row *p*‐block element bridged [1]ferrocenophanes; bottom: summary of this work.

The discovery of ferrocene was undoubtedly one of the most important milestones in organometallic chemistry and the iconic “sandwich” molecule has become standard textbook knowledge nowadays [1, 2, 3, 4, 5, 6, 7, 8]. Many derivatives have been reported ever since and have not only expanded the fundamental understanding of metallocene chemistry, but have also had a profound impact on different fields, ranging from catalysis to medicine, as well as (opto)electronic applications and functional materials [9, 10, 11, 12, 13, 14, 15, 16, 17, 18, 19, 20, 21, 22, 23, 24, 25, 26, 27, 28, 29, 30, 31, 32, 33]. One important subclass are [1]ferrocenophanes, which are utilized as monomers in ring‐opening polymerization (ROP) reactions for the preparation of main‐chain ferrocene‐based metallopolymers [12, 13, 20, 34, 35, 36, 37, 38, 39, 40, 41, 42, 43, 44, 45, 46, 47, 48]. The first isolation of a [1]ferrocenophane, exhibiting a single‐silicon atom interlinking the two cyclopentadienyl (Cp) groups, dates back to 1975 (Figure 1) [49], though, it took more than 15 years to realize the potential of these ring‐strained organometallic molecules for polymer synthesis. The report of a thermal ROP of sila[1]ferrocenophanes in 1992 paved the way for the preparation of well‐defined, linear, high‐molecular weight ferrocene‐based main‐chain polymers, which combine the unique properties of ferrocene with the properties of a polymer [50]. These materials have a wide range of applications and are famous for their ability to form nanostructures via crystallization driven self‐assembly. Thus, [1]ferrocenophanes have gained increasing attention in recent decades [12, 13, 20, 26, 27, 28, 29, 30, 51, 52, 53, 54, 55, 56, 57, 58, 59, 60, 61, 62, 63].

Interestingly, a large variety of different bridging atoms have been reported for [1]ferrocenophanes (see Supporting Information for further details and references), which include all conceivable heavy third and fourth row *p*‐block elements: aluminum, silicon, phosphorous, sulfur, gallium, germanium, arsenic, and selenium, as well as the fifth row *p*‐block elements: indium, tin, and antimony (Figure 1 and Table S9). However, [1]ferrocenophanes bridged by lighter *p*‐block elements are almost unknown, as the only second row *p*‐block element to be implemented as bridging motif so far, is boron [35, 38, 40, 43, 45, 46, 47, 48, 49, 64, 65, 66, 67, 68, 69]. Whether this is a synthetic issue to overcome or the result of compound instabilities due to high ring‐strain, remained somewhat unclear and has been the subject of intense debate [35, 69, 70, 71]. Since the bridging of the Cp groups in [1]ferrocenophanes leads to an angular deflection from the coplanarity and untight structure of ferrocene, small mono‐atomic linkers lead to significant ring‐strain, which can be characterized by the angles *α*, *β*, *δ*, and *θ* (Figure 1).

The angle α, which is the dihedral angle between the Cp ring planes, is commonly seen as a measure of ring‐strain. Furthermore, an increase of the tilt angle α leads to a decrease of the HOMO–LUMO gap, whereby a bathochromic shift of the lowest energy absorbance is usually observed and results in an increase of the molecules total energy, accompanied by weakening of the *ipso*‐C^Cp^─E and the Fe─Cp bonds, which facilitates ring‐opening reactions [43, 72]. The former is reflected by the color of [1]ferrocenophanes, whereby a red‐shift is observed, as the bridging element decreases in size (ferrocene: orange; Si[1]‐ and P[1]ferrocenophanes: red; S[1]ferrocenophanes: dark red to purple) [35, 43]. The latter has led to speculations that carba[1]ferrocenophanes are not isolatable at standard conditions, due to their high energy conformation, respectively, ring‐strain, as a result of the small bridging carbon atom [35]. The synthesis of ferrocenophanes usually follows one of two routes: ring‐closing reaction of dilithioferrocene and a suitable element dihalide, or the so‐called “fly trap” method, which utilizes an iron dihalide with an appropriate dianionic *ansa*‐ligand [35]. In this regard, it is noteworthy, that carba[1]magnesocenophanes, have been used in ring‐opening transmetalation polymerization (ROTP) reactions, giving access to polycobaltoceniumyl‐ and polyferrocenylmethylenes [70, 71, 73]. However, the exact mechanism of this polymerization reaction remains unclear and has fanned speculations about carba[1]ferrocenophane intermediates, although, until now, no such species could be detected [70, 71].

In this work, we report the isolation and structural authentication of a carba[1]ferrocenophane, filling one of the last missing gaps in the field of [1]ferrocenophane chemistry. Cycloheptylidene[1](di‐*tert*‐butyl)ferrocenophane, **3**, was characterized by ^1^H and ^13^C{^1^H} NMR spectroscopy, UV–vis spectroscopy, as well as HR‐MS, CV, and DSC measurements, and—most importantly—its structure was determined by single crystal X‐ray diffraction (SC‐XRD).

## 2 Results and Discussions

### 2.1 Synthesis and Characterization

**FIGURE 2:**
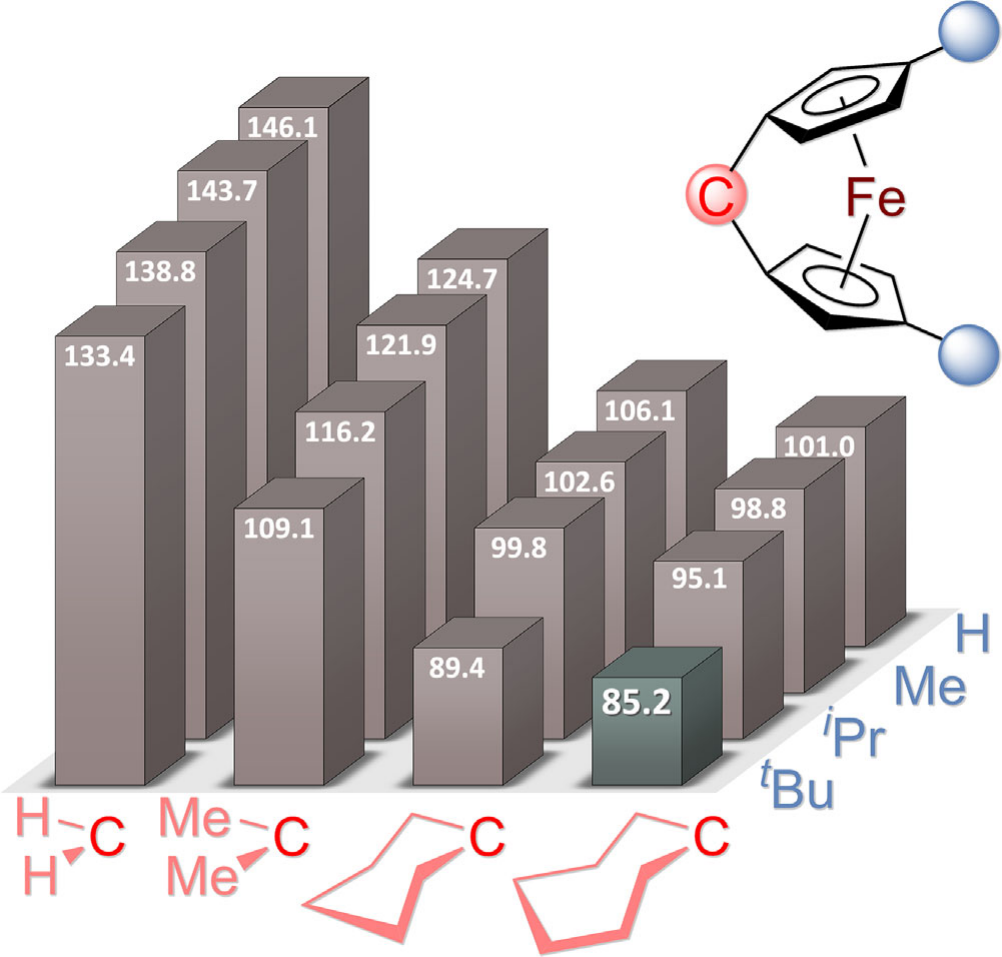
Calculated ring‐strain energies (in kJ mol^−1^) of C[1]ferrocenophanes.

As our initial experimental attempts to isolate a carba[1]ferrocenophane had failed [70, 71], we speculated that the substitution pattern, both at the bridging carbon atom and at the Cp rings, might be important. While Müller and coworkers had investigated the ring‐strain of different [1]ferrocenophanes both computationally and experimentally in 2017 [74], and their DFT calculations of a H_2_C[1]ferrocenophane and a Me_2_C[1]ferrocenophane suggested that these species could be stable at standard conditions, the influence of different substituents on the ring‐strain remained unclear. We, therefore, carried out a DFT study to computationally probe the ring‐strain of differently substituted carba[1]ferrocenophanes using Müller's method (Figure S31). These calculations suggested that the introduction of sterically demanding groups at the bridging carbon atom, as well as at the Cp rings, should lead to a substantial decrease in ring‐strain energy (Figure 2).

**FIGURE 3:**
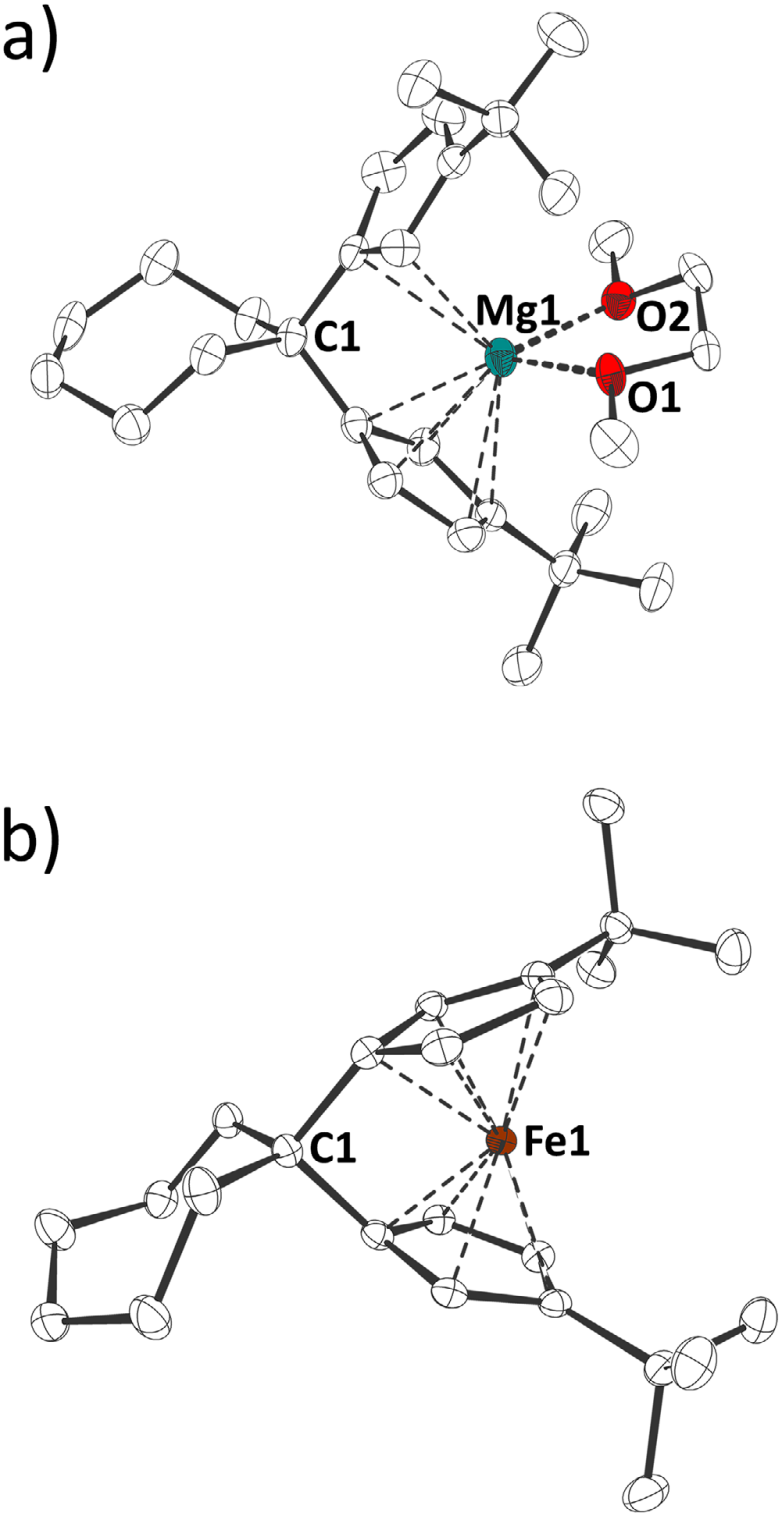
Molecular structure of (a) C[1]magnesocenophane dme adduct **2**·dme and (b) C[1]ferrocenophane **3** in the crystal (displacement ellipsoids at 50% probability level, hydrogen atoms omitted for clarity).

In fact, the ring‐strain of cycloheptylidene[1](di‐*tert*‐butyl)ferrocenophane, **3**, is predicted to be −85.2 kJ mol^−1^, which is close to Me_2_Si[1]ferrocenophane (−76.6 to −77.4 kJ mol^−1^) and substantially lower than in S[1]ferrocenophane (−114.4 to −120.8 kJ mol^−1^), both experimentally isolatable species [45, 49, 74]. Thus, this substitution pattern was identified as the most promising synthetic target and we set out to prepare the corresponding ligand **1** and subsequently C[1]magnesocenophane **2** as a transmetalation precursor. Following the general synthesis route for C[1]magnesocenophanes, **2** was obtained as a colorless and highly air‐sensitive solid from the reaction of the neutral *ansa*‐ligand 1,1’‐bis(*tert*‐butylcyclopentadienyl)cycloheptane, **1**, with *n*‐butyl‐*sec*‐butylmagnesium. Single crystals of **2·**dme were obtained from a dme solution at −25°C and enabled the structural characterization in the solid state by single‐crystal X‐ray diffraction (SC‐XRD) (Figure 3a) [75]. **2·**dme crystallizes in the monoclinic space group *P*2_1_/*c*, whereby the solid‐state molecular structure shows a ring‐slippage and η^5^/η^2^ coordination mode of the Cp groups, which is common for magnesocenophane ether adducts and results from the relatively ionic Mg─Cp bonds and the high steric demand of the two *tert*‐butyl groups [76]. ^1^H and ^13^C{^1^H} NMR spectroscopy of crystalline **2·**dme showed signal sets of two isomers. Therefore, ^1^H variable temperature NMR (VT‐NMR) spectroscopy was performed in the range of −40°C to +60°C and revealed a temperature‐dependent thermodynamic equilibrium between the two isomers (Figure S7), whereby the isomer, which is increasingly preferred at lower temperatures, is believed to be the *anti*‐isomer observed in the crystal structure. The presence of the two isomers and their interconversion can be attributed to the ionicity of the Mg─Cp bonds, which enables rotation of the Cp rings via uncapping.

**SCHEME 1:**
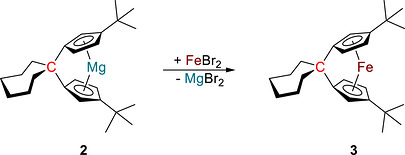
Synthesis of C[1]ferrocenophane **3** from C[1]magnesocenophane **2**.

With the transmetalation precursor **2** in hand, the stage was set for the preparation of C[1]ferrocenophane **3**, predicted to be isolatable according to DFT calculations. Thus, we treated **2** with iron(II) bromide in dme at ambient temperature, whereby the reaction mixture turned deeply red within a few seconds (Scheme 1). Noteworthy, in contrast to common ferrocenophane synthesis techniques, which often require low temperatures, this reaction proceeds selectively at room temperature. After work‐up, a highly air‐sensitive, deeply red colored oil was obtained. Characterization by one‐ and two‐dimensional multinuclear NMR spectroscopy (the NMR spectra also indicated two isomers, but no temperature dependent equilibrium was observed due to the strong covalent Fe─Cp*^t^* ^Bu^ bonds), as well as high‐resolution mass spectrometry (HR‐MS) indeed, clearly showed it to be C[1]ferrocenophane **3** (Figures S8–S12, S16). The compound appears to have a relatively low crystallinity, possibly originating from the two *tert*‐butyl groups, but careful crystallization from hexamethyldisiloxane at −25°C afforded single‐crystals, which enabled us to structurally authenticate **3** by SC‐XRD (Figures 3b and S15), unambiguously proving that a C[1]ferrocenophane is isolatable at standard conditions! [75]. **3** crystallizes in the monoclinic space group *P*2_1_/*n*, whereby the solid‐state structure shows a highly bent sandwich structure, with η^5^/η^5^ coordinated Cp groups, as to be expected. The Fe–Cp^cent^ bonds are 162.88(3) and 162.72(4) pm, which is similar to other [1]ferrocenophanes [38, 40, 64], while the *ipso*‐C^Cp^─C1 bond lengths are 156.51(19) pm and 157.05(19) pm, which is slightly longer than a common C─C single bond (∼154 pm [77]) and longer than in C[1]magnesocenophane **2** (152.45(35) to 153.57(23) pm), resulting from the high ring‐strain.

**FIGURE 4:**
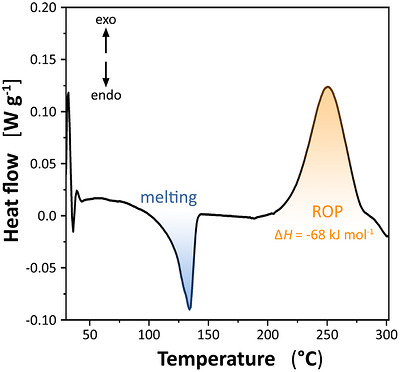
DSC diagram of C[1]ferrocenophane **3**.

As mentioned above, metallocenophanes can be characterized by the four angles *α*, *β*, *δ*, and *θ* (Tables 1 and S9). The solid‐state structure of **3** reveals a tilt angle of *α* = 36.7°, clearly indicating high ring‐strain. This is accompanied by a Cp^cent^–Fe–Cp^cent^ angle of *δ* = 151.8°, which is the smallest value reported for a [1]ferrocenophane to date. Accordingly, C[1]ferrocenophane **3** exhibits the highest angular deflection of any ferrocenophane reported so far, as a result of the small single‐carbon atom bridging motif, which is the shortest reported so far. The *ipso*‐carbon atoms of the Cp rings in [1]ferrocenophanes typically exhibit an upfield shift in the ^13^C{^1^H} NMR spectrum, which is also observed for **3** (δ^13^C(*ipso*‐C^Cp^) = 41.0 ppm) [35, 37, 40]. While C[1]ferrocenophane **3** is air‐sensitive, it is thermally quite robust. For instance, solid **3** can be stored under an atmosphere of dry argon for at least several months, and solutions of **3** in thf and toluene are stable at ambient temperatures for at least several days. Even in boiling toluene, no decomposition is observed after 3 h. The high thermal stability of **3**, despite its high ring‐strain, is also apparent from differential scanning calorimetry (DSC) measurements (Figure 4). DSC revealed an endothermic melting at 134°C and an exothermic peak at 250°C, which corresponds to Δ*H* = −68 kJ mol^−1^. This is lower than the reported Δ*H* value of a B[1]ferrocenophane (Δ*H* = −95 kJ mol^−1^), which is the [1]ferrocenophane with the second highest angular deflection (*α* = 32.4°, *δ* = 155.2°) [38, 44].

**TABLE 1:** Selected structural parameters and *λ*_max_ of [1]ferrocenophanes.

E[1]ferrocenophanes	*α* (°)	*β*/*β*' (°)	*δ* (°)	*θ* (°)	*λ*_max_ (°)	References
(PyMe_2_Si)R_2_C**Al**[1]	14.8	39.6/40.5	168.2	94.8	—^a^	47
Ph_2_ **Si**[1]	19.1	40.0	167.3	99.2	483^b^	78, 79
Ph**P**[1]	26.9 (26.7)	32.1/32.3 (32.4/31.6)	159.8 (159.9)	90.7 (90.6)	497^c^	48, 64, 66
**S**[1]	31.1	29.1/28.7	156.9	89.0	504^c^	40, 45
*^i^* Pr_2_N**B**[1]	32.4	33.8/33.4	155.2	100.1	479^c^	38, 44
**3** ((C_6_H_12_)**C**[1])	36.7	29.4/29.7	151.8	97.0	511^c^	this work

^a^ Not reported.

^b^ UV–vis data reported for cyclohexane solutions.

^c^ UV–vis data reported for hexane solutions.

Cyclic voltametric measurements (CV) of **3** in 1,2‐difluorobenzene versus Fc/Fc^+^ showed a reversible one‐electron oxidation that can be assigned to the ferrocenophane (Fe^2+^)/ferrocenophanium (Fe^3+^) redox couple (Figures S24–S27). The lower redox potential of *E*_1/2_ = −405 mV compared to ferrocene can be attributed to the inductive donation of electrons from the two *tert*‐butyl groups and the cycloheptylidene group, as well as the tilting and concomitant decrease of the HOMO–LUMO gap [40, 80, 81, 82]. As there is a steady gradient in color of [1]ferrocenophanes with a decrease of the atomic size of the bridging element and an increase of the tilt angle, UV–vis spectroscopy of a solution of **3** in hexane in the range of 200–800 nm was performed. A significant red shift of the lowest energy absorbance band is observed (*λ*_max_ = 511 nm) compared to a B[1]ferrocenophane (*λ*_max_ = 479 nm) or S[1]ferrocenophane (*λ*_max_ = 504 nm), justifying the characteristic deeply red color of **3** (Figures S21–S22). This is attributed to a decrease in the HOMO–LUMO gap, whereas the high intensity in color of **3** can be explained by the Laporte rule relaxation as the centrosymmetry of ferrocene is destroyed due to the bending, whereby *d*–*d* transitions become more allowed [35, 38, 40, 43].

**FIGURE 5:**
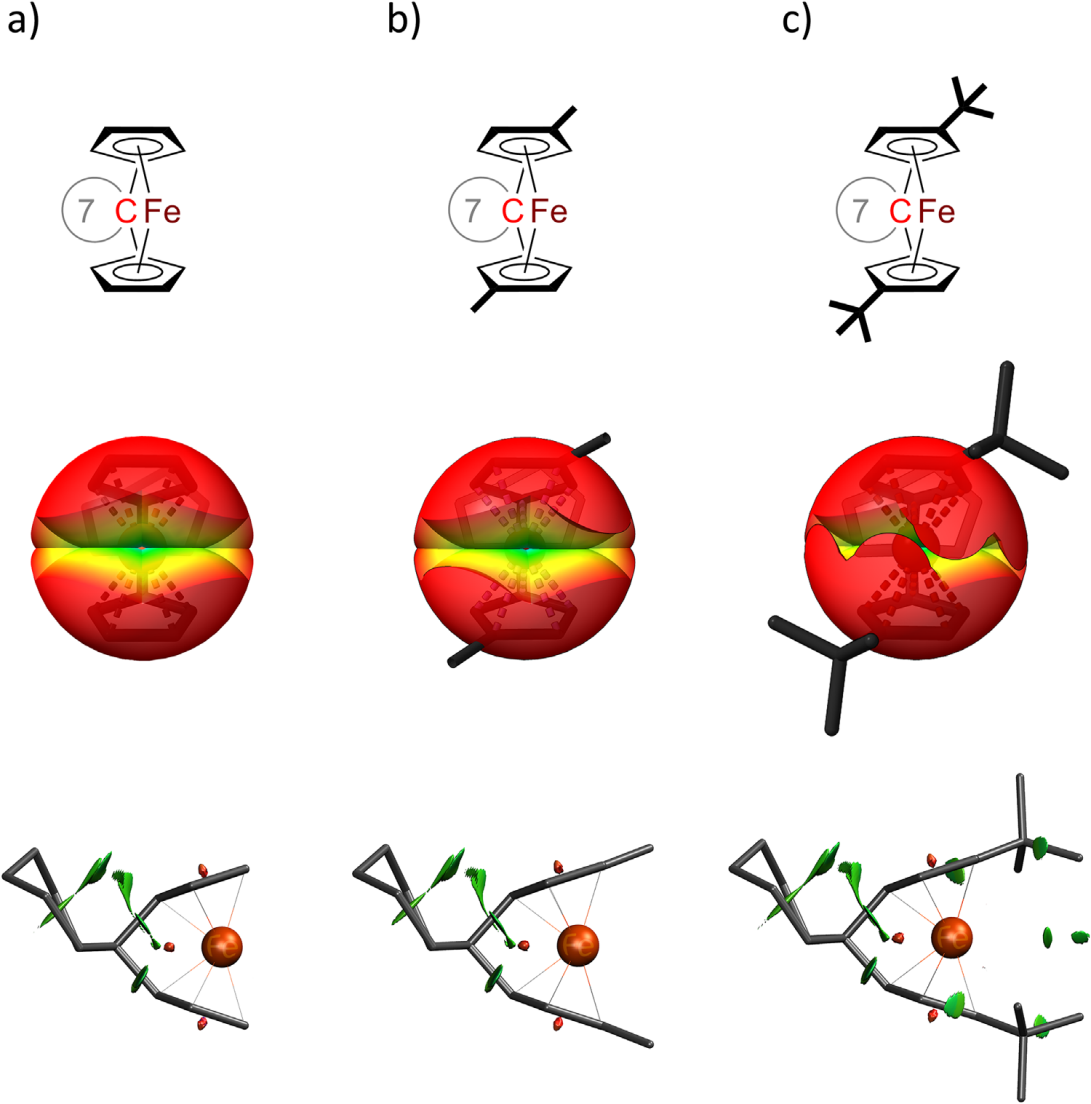
Lewis structure illustrations (top), 3D buried volume maps (center) and NCI plots (bottom) of a) Cp^H^‐substituted derivative of **3**, b) Cp^Me^‐substituted derivative of **3** and c) **3**, calculated at B3PW91‐D3(BJ)/def2‐TZVP//B3PW91‐D3(BJ)/def2‐SVP level of theory.

To gain further insight into the electronic properties of **3**, we analyzed its chemical bonding with different DFT techniques (see Supporting Information for further details and references). In comparison to other [1]ferrocenophanes, **3** exhibits similar partial charges and bond orders, suggesting a similar electronic structure and charge distribution (Tables S1 and S2). However, the analysis of Mayer's Effective Fragment Orbitals (EFOs) and their occupations uncovered differences [83, 84]. These localized orbitals have been widely employed across a range of chemical systems [85, 86, 87], and have been particularly useful to quantify the donor–acceptor character of different types of interactions [88]. In **3**, we identified three formally occupied *d*‐type EFOs at the iron center, with the $d_{z^{2}}$ EFO being the most occupied orbital (1.77 e), followed by the $d_{x^{2}-y^{2}}$ (1.63 e) and *d*_xy_ (1.51 e). Accordingly, substantially lower occupations are computed for the two formally unoccupied EFOs, *d*_xz_ (0.71 e) and *d*_yz_ (0.67 e). This pattern is consistent for all analyzed [1]ferrocenophanes (Table S3) and matches a *d* ^6^ configurated Fe^2+^ center (Figure S32). Noteworthy, occupations of the *d*_xz_ EFO show a correlation with the Fe–Cp^cent^ distances (Figure S33), as shorter Fe–Cp^cent^ distances favor stronger donation from the π system of the Cp groups to the *d*_xz_ orbital at the iron center. To gain further insight into the Fe–Cp bonding in **3**, we performed an Energy Decomposition Analysis (EDA) and compared the results to the Cp^H^‐substituted and Cp^Me^‐substituted theoretical derivatives (Tables S4–S6). According to EDA, **3** features the strongest orbital interaction (Δ*E*_*orb*_ = −2388 kJ mol^−1^) but the weakest total interaction among the analyzed complexes (Cp^H^ derivative: Δ*E*_*int*_ = −3406 kJ mol^−1^; Cp^Me^ derivative: Δ*E*_*int*_ = −3411 kJ mol^−1^; **3**: Δ*E*_*int*_ = −3377 kJ mol^−1^). This is due to the high Pauli repulsion and weak electrostatic attraction arising from the short Fe─Cp^cent^ bonds. Nonetheless, **3** has a dissociation energy similar to other [1]ferrocenophanes, due to low geometrical strain (i.e., preparation energy). Thus, we hypothesize that the stability of **3** originates not only from orbital interactions, but also from steric shielding and possibly non‐covalent interactions. The former is illustrated in the buried volume maps [89] (Figures 5 (center) and S34), which reveal that the *tert*‐butyl substituents of **3** effectively shield the iron center, which is not the case in the Cp^H^‐ and Cp^Me^‐substituted theoretical derivatives. Furthermore, the *tert*‐butyl groups not only provide steric protection but also exhibit attractive non‐covalent interactions (NCI), as indicated by NCI plots [90] in combination with a pairwise orbital decomposition analysis [91]. These NCI plots of **3** show stabilizing van der Waals surfaces, arising from C─H⋯C─H interactions of the *tert*‐butyl groups (Figure 5 (bottom) and Table S8). Thus, the high thermal stability of **3** is a consequence of a synergistic interplay of the increased Fe─Cp bond strength due to enhanced π(Cp)→*d*_xz_(Fe) orbital interactions, steric shielding of the iron center, and attractive non‐covalent interactions in the ligand system, all arising from the *tert*‐butyl substituents.

### 2.2 Ring‐Opening Reactions

**SCHEME 2:**
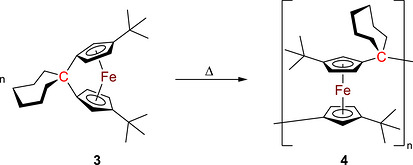
Ring‐opening oligomerization of C[1]ferrocenophane **3**.

A common route to obtain polyferrocenylsilanes is the (living) anionic ROP of Si[1]ferrocenophanes via treatment with alkyllithium reagents [51, 52]. This reaction is initiated by the nucleophilic attack of the carbanion at the electropositive silicon atom [51, 52, 92]. However, since the *ipso*‐C^Cp^─C1 bond in C[1]ferrocenophane **3** is not polarized, no reaction was observed upon treatment of **3** with butyllithium reagents. This is not surprising, as analogously no anionic ROP is induced when ethylene bridged C[2]ferrocenophanes are reacted with organolithium compounds, in which case a lithiation of the Cp rings occurs [92]. That no lithiation of the Cp rings in **3** took place can be attributed to the steric shielding and an inductive effect of the *tert*‐butyl groups. Following this, photo‐enabled anionic ROP was attempted by using different light sources (365 and 450 nm) and different initiators (LiCp, NaCp, KCp*), as this is reported for both Si[1]ferrocenophanes and C[2]ferrocenophanes [23, 57, 92]. However, likewise no reaction was observed in case of **3**. This is in agreement with relatively strong Fe─Cp bonds, originating from the *tert*‐butyl substituents, as suggested by the DFT calculations. As the DSC measurements showed an exothermic peak at ∼250°C (Figure 4), which can be rationalized by a ring‐opening reaction of **3**, we investigated the possibility of thermal ROP of **3** by heating neat samples to 250°C (Scheme 2).

In fact, a distinct color change was observed, and an air‐ and moisture‐stable, thf soluble, brownish‐colored oil, **4**, was obtained and characterized by ^1^H and ^13^C{^1^H} NMR spectroscopy, UV–vis spectroscopy, gel permeation chromatography (GPC), matrix‐assisted laser desorption ionization time‐of‐flight mass spectrometry (MALDI‐ToF MS), CV measurements, thermogravimetric analysis (TGA), and differential scanning calorimetry (DSC). In agreement with the observed color change, UV–vis spectroscopy of **4** reveals a hypsochromic shift of the lowest energy absorbance compared to **3** (*λ*_max_ = 457 nm, Figure S23), as a result of the loss of tilting and the formation of coplanar ferrocene moieties. GPC measurements revealed a molar mass distribution of only oligomeric species (*M*_w_ = 1800 g mol^−1^; Figure S19). However, as GPC measurements can underestimate the molecular weights of **4** [70], MALDI‐Tof mass spectrometry was performed and confirmed the formation of oligomeric species with up to four repeat units (Figure S20). Furthermore, CV measurements of **4** show one reversible oxidation and reduction potential with *E*_1/2_ = −58 mV (Figures S28–S30), suggesting there is no communication between the ferrocene moieties in oligomeric **4** [70]. TGA measurements show a distinct weight loss at 238°C (Figure S17), and DSC measurements reveal neither a melting point nor a glass transition temperature *T*_g_ in the analyzed temperature range of −80°C to 120°C (Figure S18).

## 3 Conclusion and Outlook

Over 50 years after the first report of a Si[1]ferrocenophane, all third‐ and fourth‐row elements of groups 13–16, as well as the fifth‐row elements In, Sn and Sb have been incorporated as bridging atoms in [1]ferrocenophanes. However, until now, boron remained the only second‐row *p*‐block element to be installed in [1]ferrocenophanes, which was speculated to be a result of ring‐strain and concomitant instability. The isolation of C[1]ferrocenophane **3** refutes this and fundamentally expands the field of [1]ferrocenophanes, while at the same time, opening the door for speculations about N[1]ferrocenophanes. The synthesis follows the “fly‐trap” route, using the corresponding C[1]magnesocenophane **2** as a transmetalation precursor. Cycloheptylidene[1](di‐*tert*‐butyl)ferrocenophane **3** was obtained as a deeply red colored solid and—while air‐sensitive—proved to be surprisingly thermally robust, undergoing ring‐opening reactions only at temperature above 200°C.

The reason why thermal ROP of **3** only yielded low molecular weight oligomers, can most likely be attributed to the two *tert*‐butyl groups and corresponding immense steric demand of the repeat unit. To improve ROP behavior and obtain higher molecular weight materials, future work will focus on the synthesis of C[1]ferrocenophanes with a less sterically demanding substitution pattern.

## Author Contributions

**Aylin Feuerstein**: investigation, writing – original draft, visualization, data curation, writing – review and editing. **Sergi Danés**: investigation, writing – review and editing. **Kathrin Kolling**: investigation. **Bernd Morgenstern**: investigation. **Markus Gallei**: writing – review and editing, resources. **André Schäfer**: writing – review and editing, conceptualization, funding acquisition, project administration, visualization, supervision, resources.

## Funding

Saarland University, German Research Foundation (DFG): SCHA1915/7‐1; GRK3082; INST163/445‐1; INST256/506‐1, Spanish Ministry of Science, Innovation and Universities (MICIU): PID2022‐140666NB‐C22, Horizon Europe PHOTOSINT project: 101118129.

## Conflicts of Interest

The authors declare no conflicts of interest.

## Supporting information




**Supporting File 1**: Data that supports the findings of this study are available as Supporting Information. This includes experimental procedures, NMR data and spectra, X‐ray crystallographic details, HR‐ and MALDI‐ToF MS data, TGA and DSC data, UV–vis data, CV data, and computational details, as well as additional references.^[93–136]^

**Supporting File 2**: anie73326‐sup‐0002‐DataFile.zip.

## Data Availability

The data that supports the findings of this study are available in the Supporting Information of this article.
