# Supplementary material for: The Missing Link in Ferrocenophane Chemistry: Isolation of a Carba[1]Ferrocenophane
Source: Angew Chem Int Ed Engl. 2026 Jul 12;65(34):e2211037. doi: 10.1002/anie.2211037 (PMC13480525; doi:10.1002/anie.2211037)
Supplement: Supplementary file 1 [file ANIE-65-e2211037-s001.pdf]

Supporting Information

for

The Missing Link in Ferrocenophane Chemistry: Isolation of a Carba[1]ferrocenophane

Aylin Feuerstein[a], Sergi Danés*[b],[c], Kathrin Kolling[a],[d], Bernd Morgenstern[a], Markus Gallei*[a],[d], André Schäfer*[a]

[a] Saarland University, Faculty of Natural Sciences and Technology, Department of Chemistry, Campus Saarbrücken, 66123 Saarbrücken, Germany.

[b] Institute of Chemical Research of Catalonia (ICIQ), Barcelona Institute of Science and Technology (BIST), Avinguda Països Catalans 16, 43007 Tarragona, Spain.

[c] Institute of Computational Chemistry and Catalysis (IQCC), University of Girona, 17003 Girona, Catalonia, Spain.

[d] Saarland Center for Energy Materials and Sustainability – Saarene, Saarland University, Campus Saarbrücken, 66123 Saarbrücken, Germany.

Table of Content

1. **Synthetic details and NMR data** **S2**

2. **NMR spectra** **S7**

3. **SC-XRD data** **S15**

4. **HR-MS data** **S19**

5. **TGA and DSC data** **S20**

6. **GPC data** **S22**

7. **MALDI-ToF MS data** **S23**

8. **UV-vis data** **S24**

9. **CV data** **S26**

10. **Computational details** **S30**

11. **Overview of selected [1]ferrocenophanes** **S44**

12. **References** **S49**

1. Synthetic details and NMR data

1.1 General details for Synthesis

All manipulations were carried out under an argon inert gas atmosphere using Schlenk line techniques or a glove box, except for the workup of ligand **1**. Solvents were purified using an MBraun Solvent Purification System.

Starting chemicals were purchased from Alfa Aesar, abcr or Merck / Sigma-Aldrich and were used as received. Monomeric cyclopentadiene was obtained from dicyclopentadiene by thermal cracking and distillation. Unless otherwise stated, all manipulations were carried out under an argon inert gas atmosphere using Schlenk line techniques or a glove box. Solvents were purified using an MBraun Solvent Purification System. NMR spectra were recorded on Bruker Avance III 400 and Bruker Avance III 300 spectrometers. Chemical shifts (δ) are reported in parts per million (ppm), relative to $\delta^{1}H(SiMe_4) = 0$ and $\delta^{13}C(SiMe_4) = 0$ and the spectra were referenced to the solvent signals.[93]

1.2 Compounds synthesis and characterization

***tert*-Butylcyclopentadiene:**

tert-Butylcyclopentadiene was synthesized according to a modified literature known procedure.[94]

A suspension of 20.0 g of sodium amide (513 mmol, 1.00 eq) in 100 ml thf was cooled to 0 °C and 55 ml of monomeric cyclopentadiene (44.0 g, 666 mmol, 1.30 eq) were slowly added, whereby a purple-colored solution was obtained. The reaction mixture was stirred overnight and allowed to warm to room temperature. All volatiles were removed *in vacuo* and the residue was dissolved in thf. The purple-colored solution was heated to 60 °C and 90.0 ml of *tert*-butyl bromide (110 g, 801 mmol, 1.56 eq) were added dropwise. The reaction mixture was allowed to cool to room temperature and stirred overnight. Subsequently, 50 ml of saturated aqueous ammonium chloride solution, 50 ml of water and 50 ml of pentane were added. The organic phase was separated, the aqueous phase was extracted with pentane (3 × 50 ml) and the combined organic phases were dried over magnesium sulfate. The solvent was removed under reduced pressure and *tert*-butylcyclopentadiene was obtained as a colorless liquid after distillation under reduced pressure (66 mbar, 50-80 °C). Yield: 27.3 g (224 mmol, 44 %).

^{1}H NMR (400.13 MHz, $CDCl_3$, 293 K, δ in ppm): 6.38-6.14 (m, 3H, Cp-H); 2.92 (m, 2H, Cp-H); 1.17 (s, 9H, CH_3).

$^{13}C\{^1H\}$ NMR (100.61 MHz, $CDCl_3$, 293 K, δ in ppm): 154.1 ($C_4H_5\underline{C}$-$C(CH_3)_3$); 136.4 (Cp); 134.6 (Cp); 132.5 (Cp); 129.9 (Cp); 126.9 (Cp); 116.7 (Cp); 58.2 ($\underline{C}(CH_3)_3$); 51.6 ($\underline{C}(CH_3)_3$); 47.1 (Cp); 44.5 (Cp); 42.2 (Cp); 35.2 ($\underline{C}H_3$); 31.9 ($\underline{C}H_3$).

Sodium *tert*-butylcyclopentadienide:

A suspension of 9.50 g of sodium amide (244 mmol, 1.00 eq) in 100 ml thf was cooled to 0 °C and 30.0 g *tert*-butylcyclopentadiene (245 mmol, 1.00 eq) were slowly added. The reaction mixture was stirred overnight and slowly allowed to warm to room temperature. All volatiles were removed *in vacuo* and sodium *tert*-butylcyclopentadienide was obtained as a soft pink solid. Yield: 34.0 g (236 mmol, 96 %).

^1H NMR (400.13 MHz, thf-d_8, 293 K, δ in ppm): 5.57-5.59 (m, 2H, Cp-$\underline{H}$); 5.50-5.52 (m, 2H, Cp-$\underline{H}$); 1.21 (s, 9H, $C\underline{H_3}$).

$^{13}C\{^1H\}$ NMR (100.61 MHz, thf-d_8, 293 K, δ in ppm): 131.1 ($C_4H_4\underline{C}$-$C(CH_3)_3$); 101.9 (Cp); 99.9 (Cp); 34.3 ($\underline{C}(CH_3)_3$); 32.8 ($\underline{C}H_3$).

3-*tert*-Butyl-6-cycloheptylidenefulvene:

3-*tert*-Butyl-6-cycloheptylidenefulvene was synthesized according to a modified literature known procedure. [94]

A solution of 12.7 g of *tert*-butylcyclopentadiene (104 mmol, 1.00 eq) and 12.3 ml cycloheptanone (11.7 g, 104 mmol, 1.00 eq) in 75 ml methanol was cooled to 0 °C and 21.4 ml pyrrolidine (18.5 g, 260 mmol, 2.50 eq) were slowly added. The reaction mixture was stirred for 5 h at room temperature. Afterwards, the reaction mixture was cooled to 0 °C and slowly neutralized with glacial acid. 50 ml of water and 50 ml of hexane were added, the organic phase was separated, the aqueous phase was extracted with hexane (3 × 50 ml) and the combined organic phases were dried over magnesium sulfate. The solvent was removed under reduced pressure and 3-*tert*-butyl-6-cycloheptylidenefulvene was obtained as an orange oil after distillation *in vacuo* (0.001 mbar, 72 °C). Yield: 14.0 g (64.7 mmol, 62 %).

^1H NMR (400.13 MHz, $CDCl_3$, 293 K, δ in ppm): 6.50 (m, 2H, Cp-$\underline{H}$); 6.11 (m, 1H, Cp-$\underline{H}$); 2.79-2.75 (m, 4H, $C\underline{H_2}$); 1.64-1.72 (m, 4H, $C\underline{H_2}$); 1.53-1.56 (m, 4H, $C\underline{H_2}$); 1.18 (s, 9H, $C(C\underline{H_3})_3$).

$^{13}C\{^{1}H\}$ NMR (100.61 MHz, $CDCl_3$, 293 K, δ in ppm): 157.2 (Cp^{tBu}=<u>C</u>(C_6H_{12})); 156.4 ((C_6H_{12})C=(C_4H_3<u>C</u>-C(CH_3)$_3$)); 141.7 ((*t*BuC_4H_3)<u>C</u>=C(C_6H_{12})); 130.4 (Cp); 121.3 (Cp); 111.8 (Cp); 34.4 (<u>C</u>H_2); 34.2 (<u>C</u>H_2); 32.4 (<u>C</u>(CH_3)$_3$); 30.0 (<u>C</u>H_3); 29.4 (<u>C</u>H_2); 28.2 (<u>C</u>H_2); 28.0 (<u>C</u>H_2).

1,1'-Bis(*tert*-butylcyclopentadienyl)cycloheptane 1:

A solution of 17.4 g sodium *tert*-butylcyclopentadienide (121 mmol, 1.00 eq) in 200 ml thf was heated to 60 °C and a solution of 26.1 g of 3-*tert*-butyl-6-cycloheptylidenefulvene (121 mmol, 1.00 eq) in 15 ml thf was added dropwise. The reaction mixture was stirred at 60 °C for 48 h and subsequently allowed to cool to room temperature. 50 ml of a saturated aqueous ammonium chloride solution was added, the phases were separated, the aqueous layer was extracted with diethyl ether three times, and the combined organic layers were dried over magnesium sulfate. All volatiles were removed under reduced pressure and the residue distilled *in vacuo* (0.002-0.007 mbar, 128-150 °C). 1,1'-Bis(*tert*-butylcyclopentadienyl)cycloheptane **1** was obtained as a mixture of isomers in form of a brownish colored, highly viscous oil. Yield: 19.8 g (58.5 mmol, 48 %).

^{1}H NMR (400.13 MHz, $CDCl_3$, 293 K, δ in ppm): 6.20-5.69 (m, 4H, Cp-<u>H</u>); 2.89-2.67 (m, 4H, Cp-<u>H</u>); 2.01-1.94 (m, 4H, C<u>H$_2$</u>); 1.52 (br s, 8H, C<u>H$_2$</u>); 1.08-1.13 (m, 18H, C<u>H$_3$</u>).

$^{13}C\{^{1}H\}$ NMR (100.61 MHz, $CDCl_3$, 293 K, δ in ppm): 159.1; 158.9; 158.8; 158.3; 158.2; 157.4; 157.4; 157.3; 157.3; 156.4; 156.3; 155.4; 155.1; 154.9; 154.7; 154.6; 154.1; 126.0; 125.4; 125.3; 125.3; 125.1; 125.0; 124.9; 124.7; 124.4; 122.7; 122.7; 122.7; 121.4; 121.3; 120.8; 120.6; 119.9; 119.8; 37.5; 33.3; 33.3; 32.3; 31.0; 31.0; 31.0; 31.0; 30.3; 30.2; 30.0; 30.0; 29.8; 29.4; 28.6; 23.4; 23.3; 23.3; 23.3; 23.2.

Cycloheptylidene[1](di-*tert*-butyl)magnesocenophane 2:

A solution of 2.49 g ligand **1** (7.35 mmol, 1.00 eq) in 50 ml hexane was cooled to -50 °C and 10.5 ml of *n*-butyl-*sec*-butylmagnesium (7.35 mmol, 1.00 eq) were added. The reaction mixture was warmed to 60 °C and stirred overnight. Subsequently, all volatiles were removed *in vacuo* and magnesocenophane **2** was obtained as a colorless solid. Yield: 2.05 g (5.68 mmol, 77 %). Single crystals suitable for X-ray diffraction were obtained from a dme solution at -25 °C.

^{1}H NMR (400.13 MHz, thf-d_8, 293 K, δ in ppm): 5.79-5.78 (m, 2H, Cp-<u>H</u>); 5.69-5.71 (m, 2H, Cp-<u>H</u>); 5.08-5.17 (m, 2H, Cp-<u>H</u>); 3.43 (s, 4H, dme); 3.27 (s, 6H, dme); 2.43-2.47 (m, 2H, C<u>H$_2$</u>);

2.13-2.15 (m, 2H, C<u>H</u>$_2$); 1.76-1.80 (m, 2H, C<u>H</u>$_2$); 1.55-1.66 (m, 6H, C<u>H</u>$_2$); 1.16-1.18 (m, 18H, C<u>H</u>$_3$).

^{13}C{^{1}H} NMR (100.61 MHz, thf-d$_8$, 293 K, δ in ppm): 142.0 (C_4H_3<u>C</u>-C(CH_3)$_3$); 135.8 (Cp); 134.8 (Cp); 106.2 (Cp), 105.3 (Cp); 99.5 (Cp), 98.1 (Cp); 89.0 (Cp); 87.3 (Cp), 72.9 (dme); 59.1 (dme); 45.3 ((C_6H_{12})<u>C</u>Cp^{tBu}_2); 43.5 (<u>C</u>H_2); 41.1 (<u>C</u>H_2); 38.9 (<u>C</u>H_2); 33.4 (<u>C</u>H_3); 33.3 (<u>C</u>H_3); 32.8 (<u>C</u>(CH_3)$_3$); 32.8 (<u>C</u>(CH_3)$_3$); 31.0 (<u>C</u>H_2); 30.9 (<u>C</u>H_2); 30.8 (<u>C</u>H_2); 25.2 (<u>C</u>H_2); 24.9 (<u>C</u>H_2).

Cycloheptylidene[1](di-*tert*-butyl)ferrocenophane 3:

2.02 g of magnesocenophane **2** (5.60 mmol, 1.00 eq) and 1.21 g of iron(II) bromide (5.60 mmol, 1.00 eq) were charged into a Schlenk flask and 30 ml dme were added. The reaction mixture was stirred at room temperature for 1 h. Subsequently, all volatiles were removed *in vacuo* and 50 ml of hexane were added. The deeply red colored suspension was filtered. The solvent was removed *in vacuo* to give a deeply red colored oil (crude yield: 1.75 g, 4.46 mmol), which was recrystallized from hexamethyldisiloxane at -25 °C to yield ferrocenophane **3** as a deeply red colored crystalline solid (in some cases, single crystals suitable for X-ray diffraction were obtained). Yield: 480 mg (1.22 mmol, 22 %). For further purification, the compound may be sublimed *in vacuo* at 80 °C.

^{1}H NMR (400.13 MHz, C_6D_6, 293 K, δ in ppm): 4.20-4.23 (m, 3H, Cp-<u>H</u>); 4.12-4.14 (m, 2H, Cp-<u>H</u>); 4.07-4.08 (m, 1H, Cp-<u>H</u>); 1.85-1.92 (m, 4H, C<u>H</u>$_2$); 1.67-1.70 (m, 4H, C<u>H</u>$_2$); 1.59-1.61 (m, 4H, C<u>H</u>$_2$); 1.25 (s, 18H, C<u>H</u>$_3$).

^{13}C{^{1}H} NMR (100.61 MHz, C_6D_6, 293 K, δ in ppm): 108.4 (C_4H_3<u>C</u>-C(CH_3)$_3$); 72.1 (Cp); 70.1 (Cp); 52.7 ((C_6H_{12})<u>C</u>Cp^{tBu}_2); 41.0 (*ipso*-<u>C</u>^{Cp}); 34.8 (<u>C</u>H_2); 32.2 (<u>C</u>H_3); 31.9 (<u>C</u>(CH_3)$_3$); 30.0 (<u>C</u>H_2); 28.5 (<u>C</u>H_2); 22.5 (<u>C</u>H_2).

***Syn* isomer of Cycloheptylidene[1](di-*tert*-butyl)ferrocenophane 3:**

Following the synthesis route of C[1]ferrocenophane **3**, the mother liquor of recrystallisation was dried *in vacuo*, whereby the *syn* isomer of **3** could be isolated as deeply red colored oil.

^{1}H NMR (100.61 MHz, C_6D_6, 293 K, δ in ppm): 4.21 (m, 4H, Cp-<u>H</u>); 4.14 (m, 2H, Cp-<u>H</u>); 1.86-1.91 (m, 4H, C<u>H</u>$_2$); 1.70 (m, 4H, C<u>H</u>$_2$); 1.59-1.61 (m, 4H, C<u>H</u>$_2$); 1.25 (s, 18H, C<u>H</u>$_3$).

$^{13}C\{^1H\}$ **NMR** (100.61 MHz, C_6D_6, 293 K, δ in ppm): 109.0 ($C_4H_3\underline{C}$-$C(CH_3)_3$); 72.0 (Cp); 71.8 (Cp); 68.7 (Cp); 51.2 ((C_6H_{12})$\underline{C}$$Cp^{tBu}_2$); 41.0 (*ipso*-$\underline{C}^{Cp}$); 34.9 ($\underline{C}H_2$); 34.7 ($\underline{C}H_2$); 32.2 ($\underline{C}(CH_3)_3$); 31.9 ($\underline{C}H_3$); 30.1 ($\underline{C}H_2$); 28.6 ($\underline{C}H_2$); 22.7 ($\underline{C}H_2$); 22.3 ($\underline{C}H_2$).

Thermal ring-opening oligomerization

100 mg of C[1]ferrocenophane **3** (0.25 mmol) were heated in an evacuated, sealed Pyrex tube at 250 °C for 1 h. The tube contents became molten and turned into a brownish-orange-colored oil, which turned immobile when cooling to room temperature. The product was soluble in thf, chloroform and hexane and was characterized without further purification.

^1H **NMR** (400.13 MHz, $CDCl_3$, 294 K, δ in ppm): 5.99 (brs, 1H, Cp-$\underline{H}$); 4.05-4.16 (m, 3H, Cp-$\underline{H}$); 3.91 (brs, 2H, Cp-$\underline{H}$); 2.43 (brs, 1H, $C\underline{H_2}$); 2.12 (brs, 1H, $C\underline{H_2}$); 1.77 (brs, 2H, $C\underline{H_2}$); 1.60 (brs, 3H, $C\underline{H_2}$); 1.42-1.48 (m, 3H, $C\underline{H_2}$); 1.19 (brs, 18H, $C\underline{H_3}$).
$^{13}C\{^1H\}$ **NMR** (100.62 MHz, $CDCl_3$, 294 K, δ in ppm): 141.3 ($C_4H_3\underline{C}$-$C(CH_3)_3$); 125.8 (Cp); 102.4 (Cp); 102.3 (Cp); 88.6 (Cp); 68.7 (Cp); 68.3 (Cp); 67.0 (Cp); 66.1 (Cp); 65.8 (Cp); 65.1 (Cp); 62.5 (Cp); 32.8 ((Cp^{tBu}_2Fe)$\underline{C}$(Cp^{tBu}_2Fe)); 32.1 ($\underline{C}H_2$); 31.8 ($\underline{C}H_3$); 31.7 ($\underline{C}H_3$); 30.8 ($\underline{C}(CH_3)_3$); 30.7 ($\underline{C}(CH_3)_3$); 29.0 ($\underline{C}H_2$); 27.2 ($\underline{C}H_2$).

Attempted anionic ring-opening polymerization

Polymerization attempts were conducted in a typical setup for anionic polymerization reactions, using freshly distilled solvents and dry Schlenk tubes inside a nitrogen glovebox. Solvents were first dried over 1,1-diphenyl-*n*-hexyllithium and directly distilled into the reaction vessel. The monomer was dissolved in a small quantity of dried solvent and filtered several times over activated aluminum oxide inside the glovebox, whereby no color changes or disintegration of the monomer was noticed.

In a typical polymerization reaction attempt, 30.0 mg (76.4 µmol, 255 eq) of **3** in 2.5 ml of thf, *n*-hexane, or toluene was treated with 1.87 µl of *sec*-butyllithium solution (0.16 M in *n*-hexane, 1.00 eq) and stirred at ambient temperature for 1 to 15 h. After the given time, no color change was observed. The reaction solution was treated again with the same amount of initiator as before and stirred at ambient temperature. This process was repeated several times with increasing initiator concentrations ranging from 2 to 50 times. No polymerization was observed in any of these cases.

2. NMR spectra

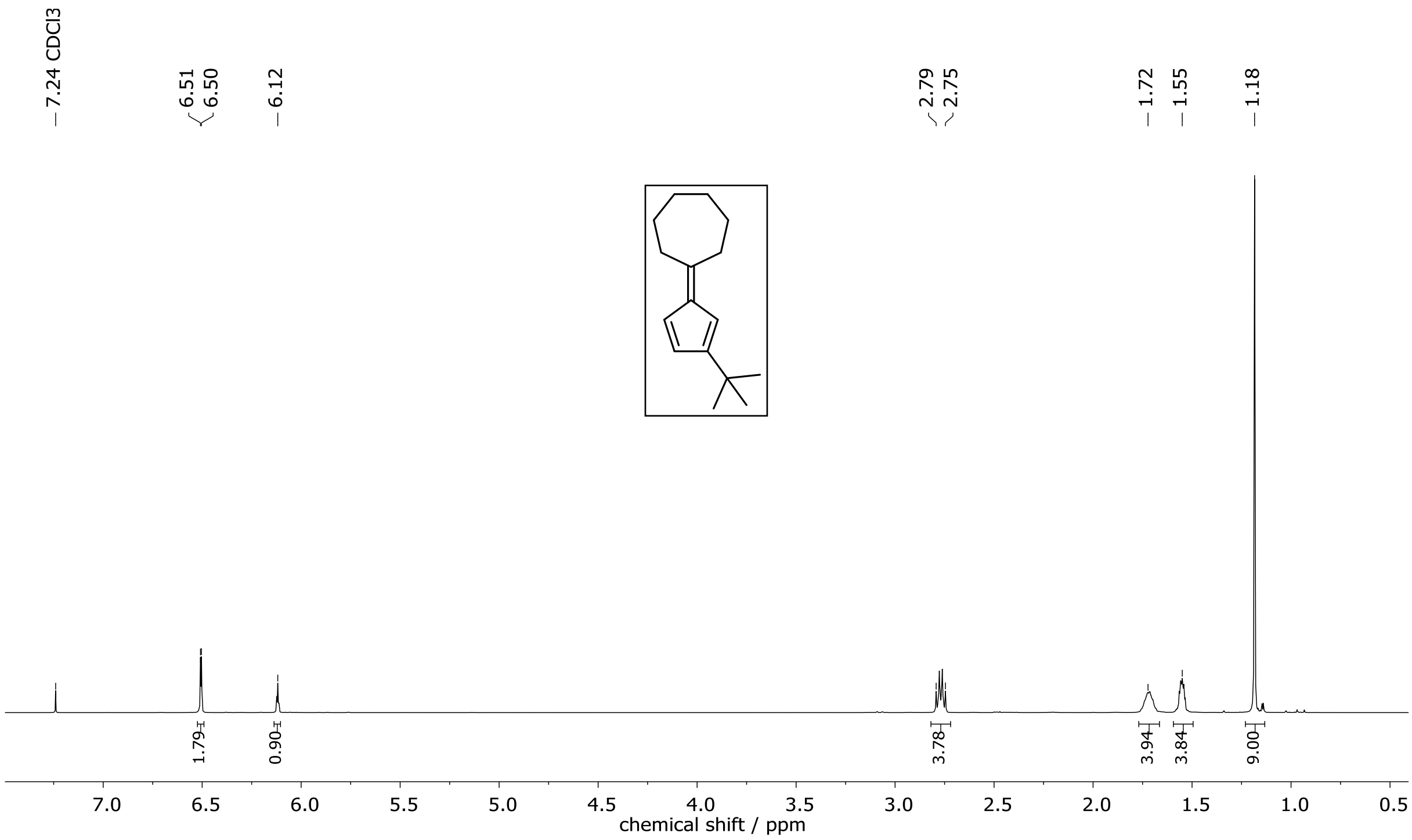

Figure S1: ^{1}H NMR spectrum (400.13 MHz, $CDCl_3$, 294 K) of 3-*tert*-butyl-6-cycloheptylidenefulvene.

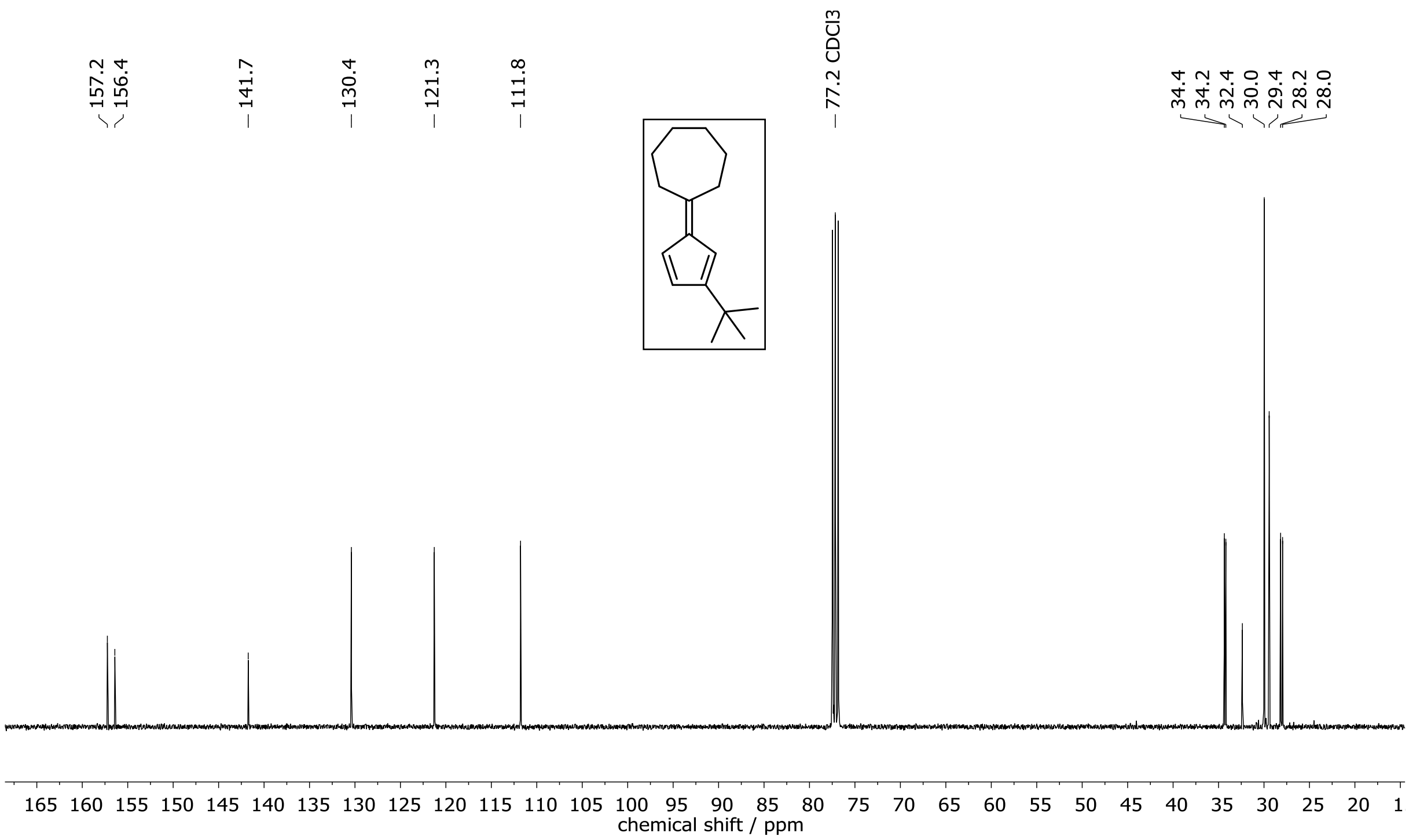

Figure S2: $^{13}C\{^{1}H\}$ NMR spectrum (100.62 MHz, $CDCl_3$, 294 K) of 3-*tert*-butyl-6-cycloheptylidenefulvene.

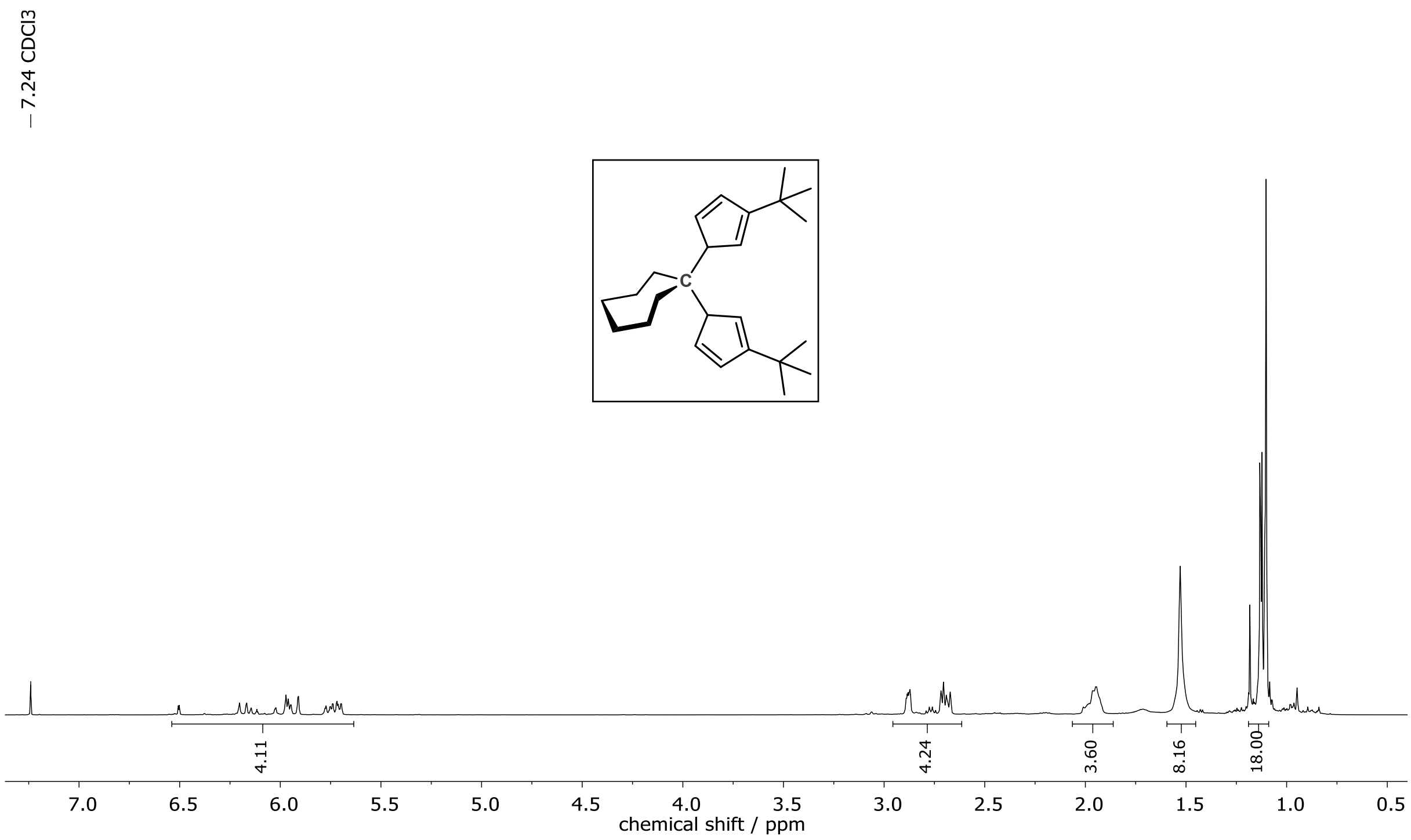

Figure S3: ^{1}H NMR spectrum (400.13 MHz, $CDCl_3$, 294 K) of **1** (mixture of isomers; insert shows one isomer).

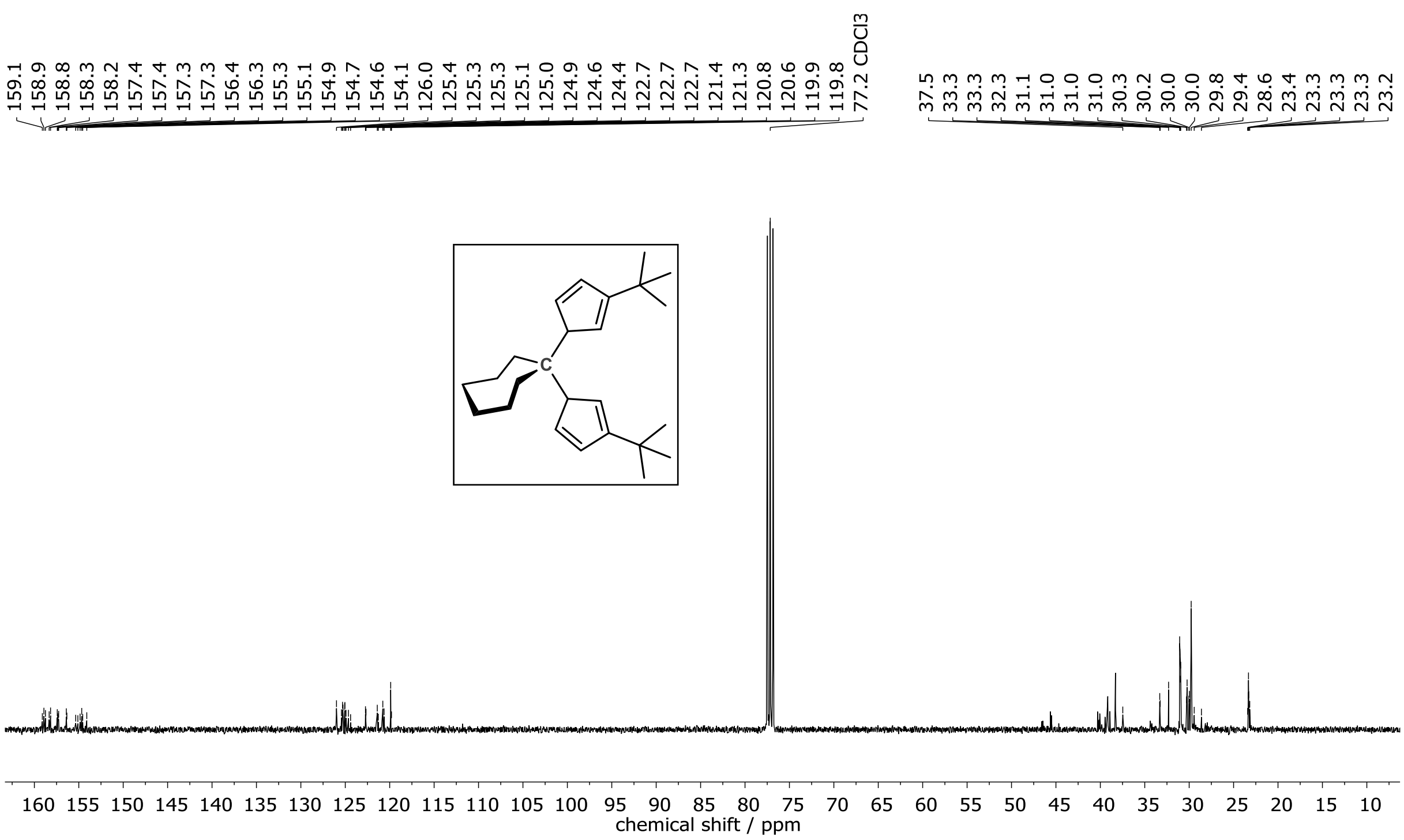

Figure S4: $^{13}C\{^{1}H\}$ NMR spectrum (100.62 MHz, $CDCl_3$, 294 K) of **1** (mixture of isomers; insert shows one isomer).

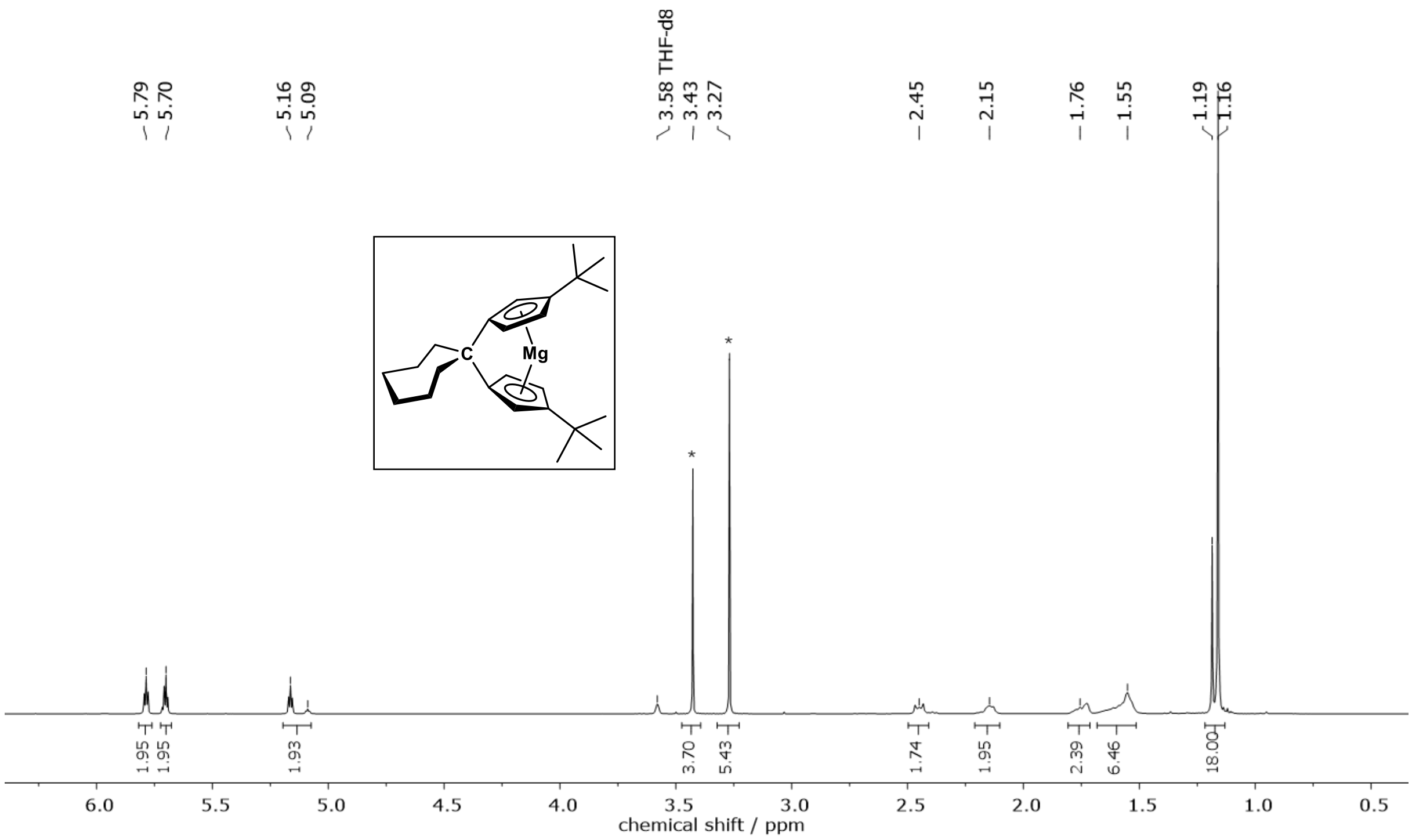

Figure S5: ^{1}H NMR spectrum (300.13 MHz, thf-d_8, 298 K) of **2**•dme crystals (* = dme).

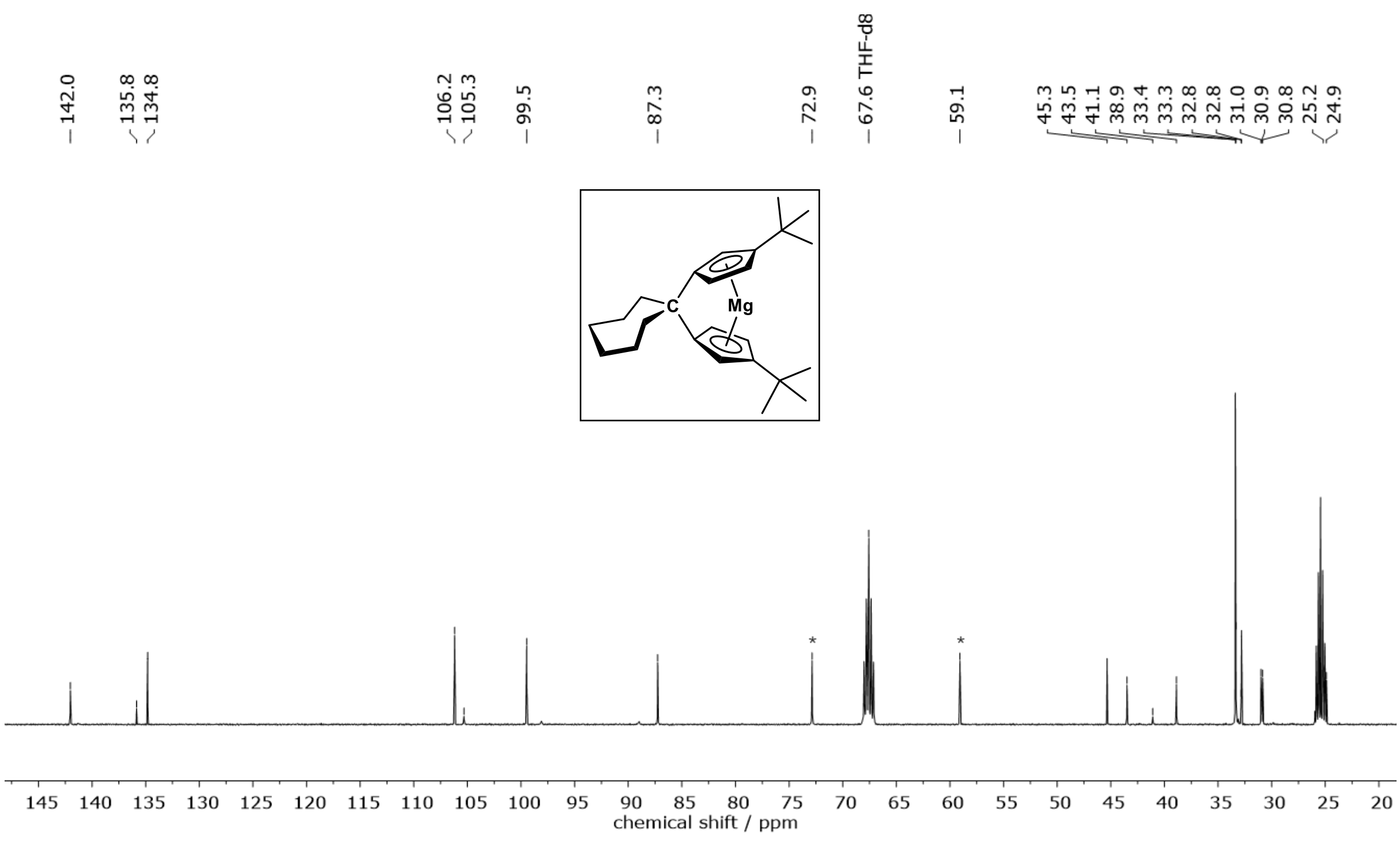

Figure S6: $^{13}C\{^{1}H\}$ NMR spectrum (100.62 MHz, thf-d_8, 294 K) of **2**•dme crystals (* = dme).

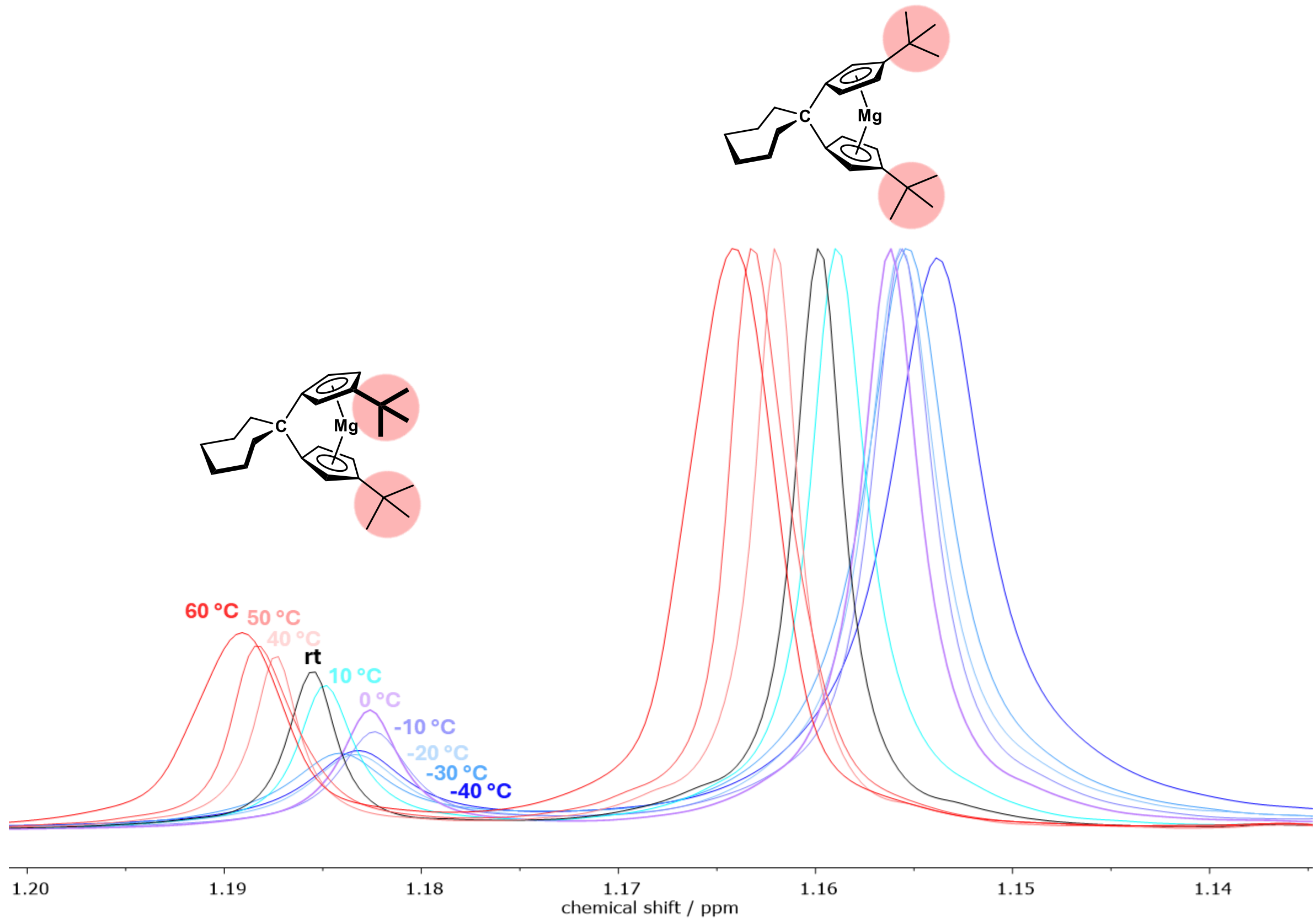

Figure S7: VT ^{1}H NMR spectra (300.13 MHz, thf-d_8) of **2**·dme crystals in the range of 1.13 ppm to 1.20 ppm at 60 °C, 50 °C, 40 °C, room temperature, 10 °C, 0 °C, -10 °C, -20 °C, -30 °C and -40 °C.

Figure S7 shows the VT ^{1}H NMR spectra of a solution of **2**·dme crystals in thf-d_8, enlarged in the region of the *tert*-butyl groups (1.13 ppm to 1.20 ppm). As the Mg–Cp bonds exhibit a high ionic character, the Cp rings can rotate around the *ipso*-C^{Cp}–C1 bonds in solution, resulting in different isomers. A decrease in temperature leads to the formation of the *anti* isomer (right), while an increase in temperature leads to the formation of the *syn* isomer (left). Thus, both isomers are in a temperature dependent equilibrium (the signal shift is a result of temperature).

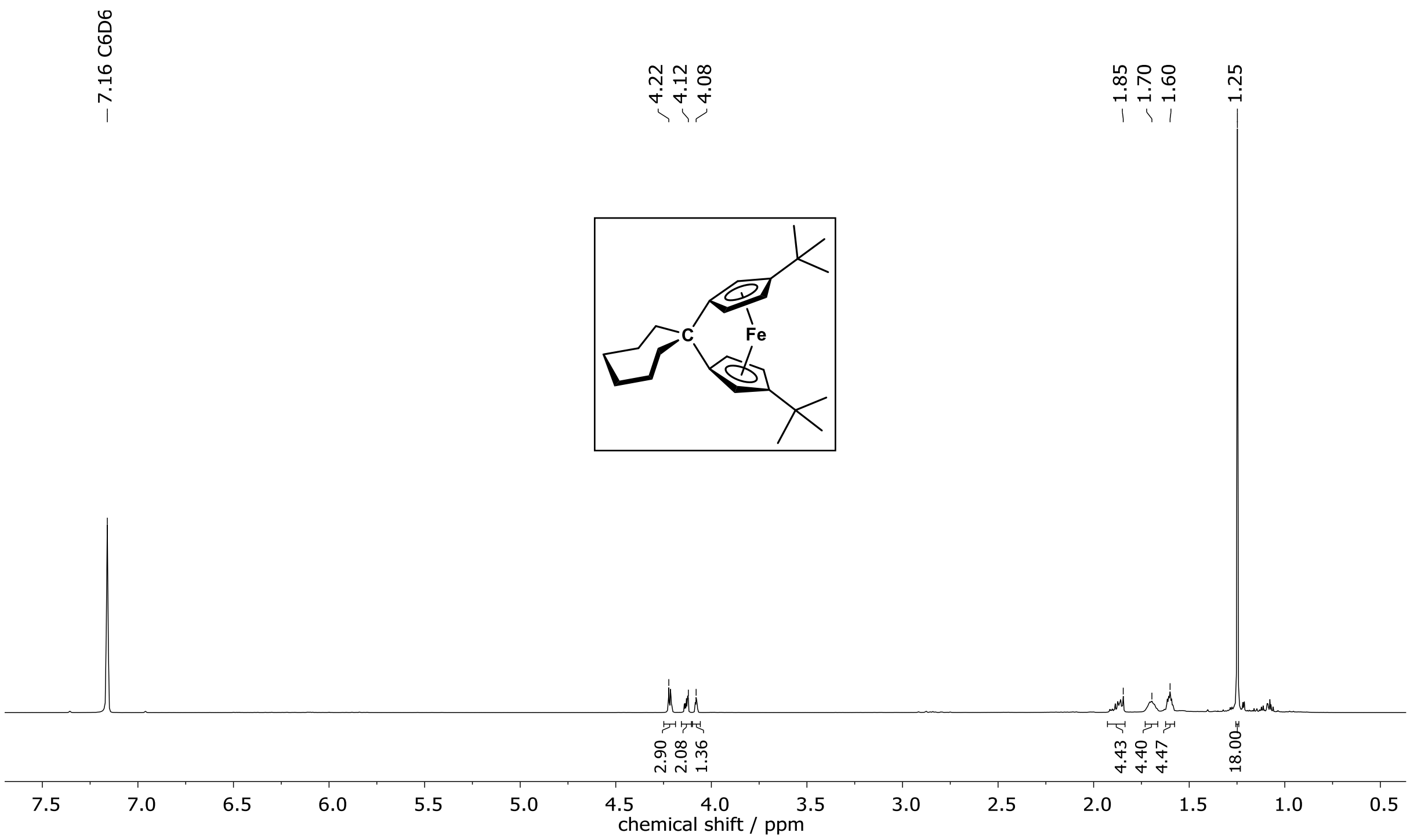

Figure S8: ^{1}H NMR spectrum (400.13 MHz, C_6D_6, 294 K) of **3**.

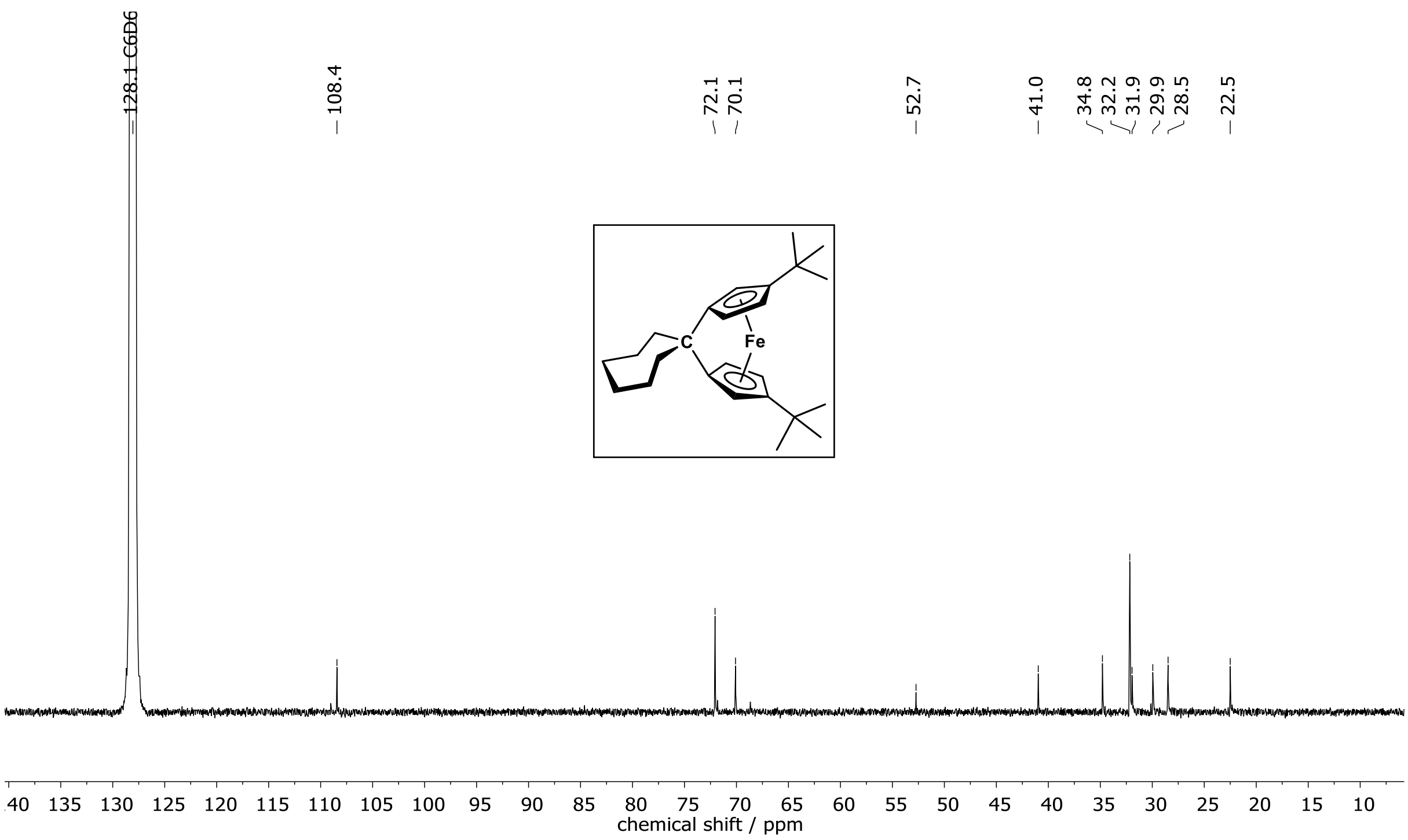

Figure S9: $^{13}C\{^{1}H\}$ NMR spectrum (100.62 MHz, C_6D_6, 294 K) of **3**.

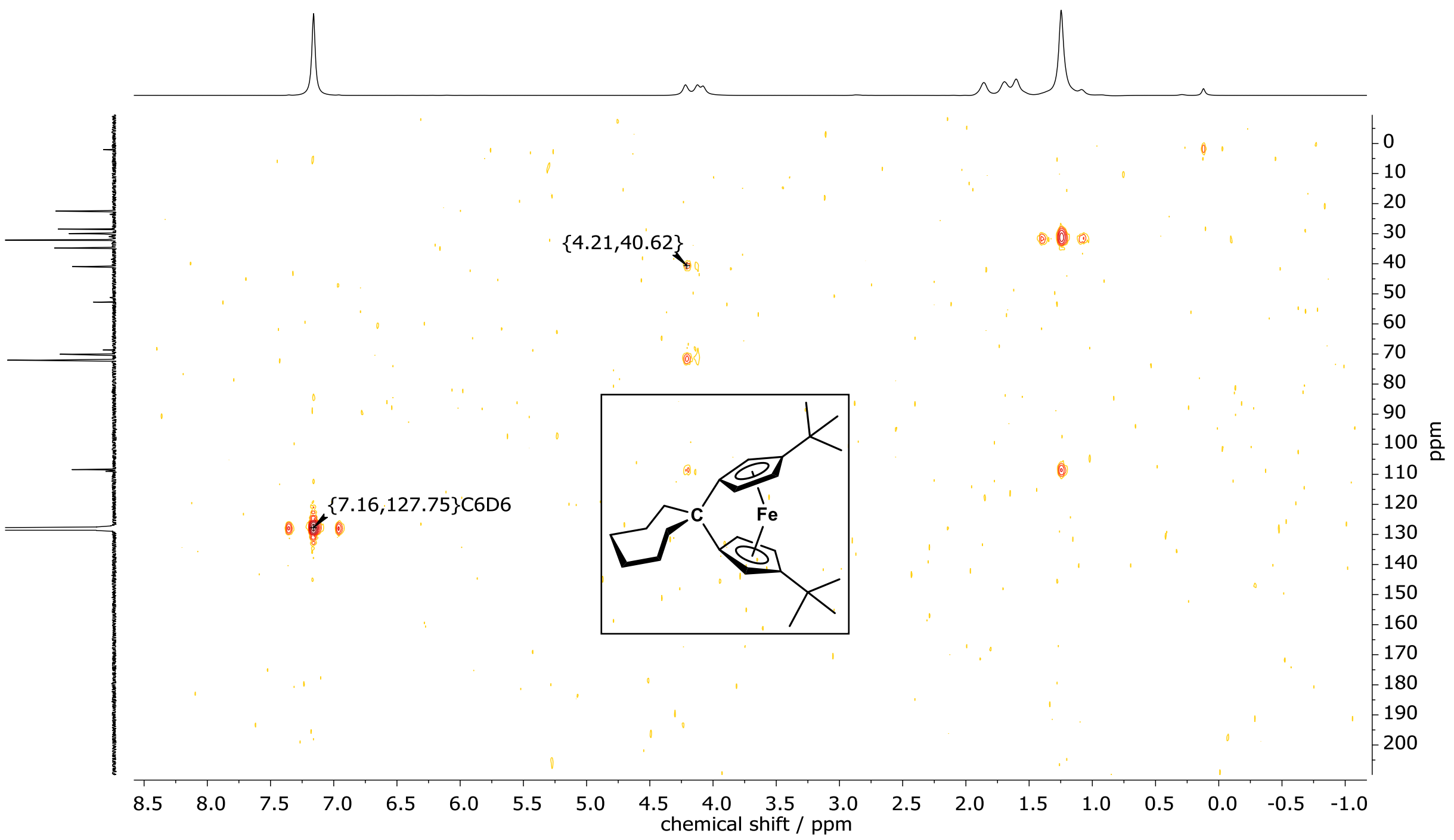

Figure S10: ^{1}H-$^{13}C\{^{1}H\}$ HMBC NMR spectrum (400.13 MHz, 100.62 MHz, C_6D_6, 294 K) of **3**, highlighting the *ipso*-carbon atom of the Cp moieties.

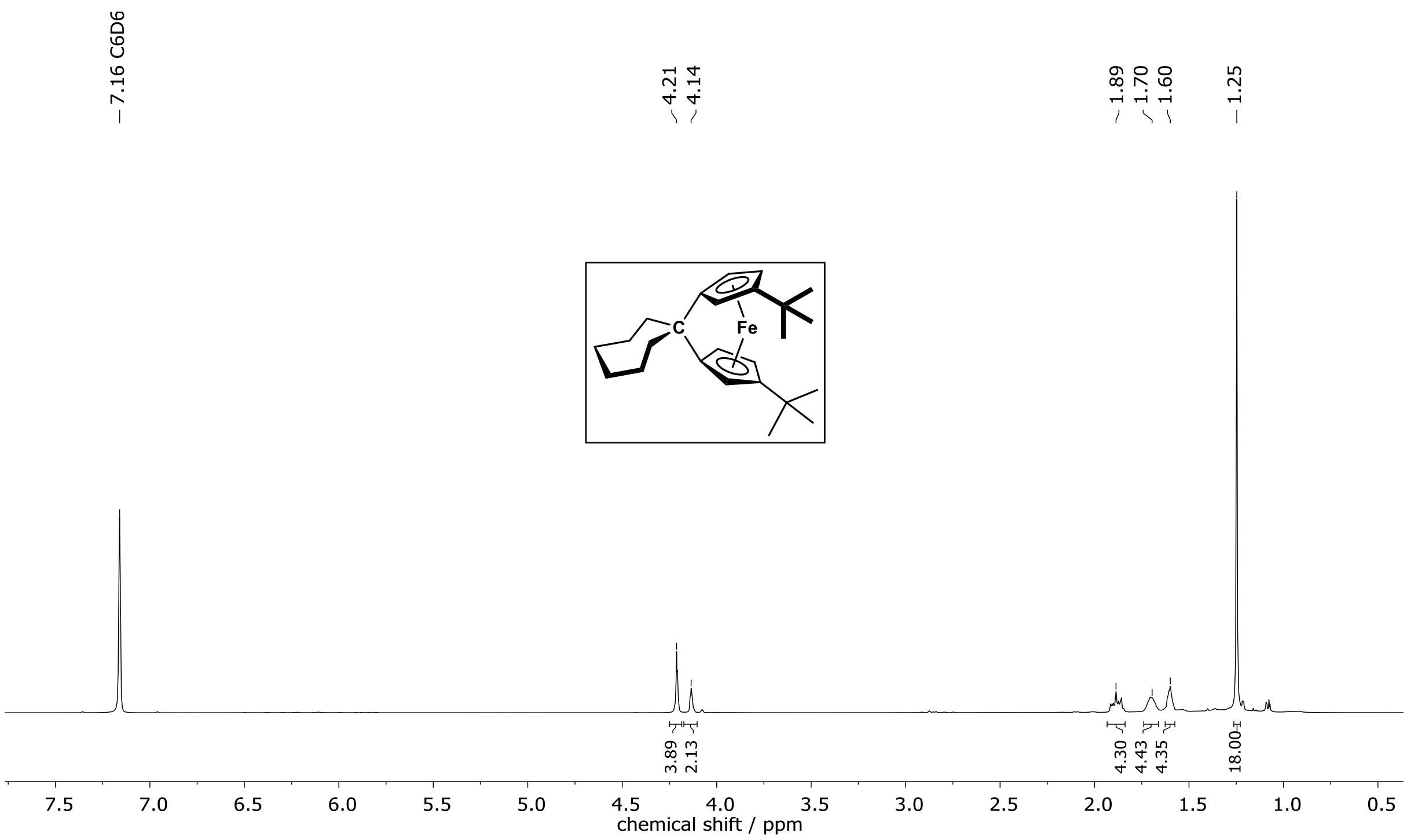

Figure S11: ^{1}H NMR spectrum (400.13 MHz, C_6D_6, 294 K) of the *syn* isomer of **3**.

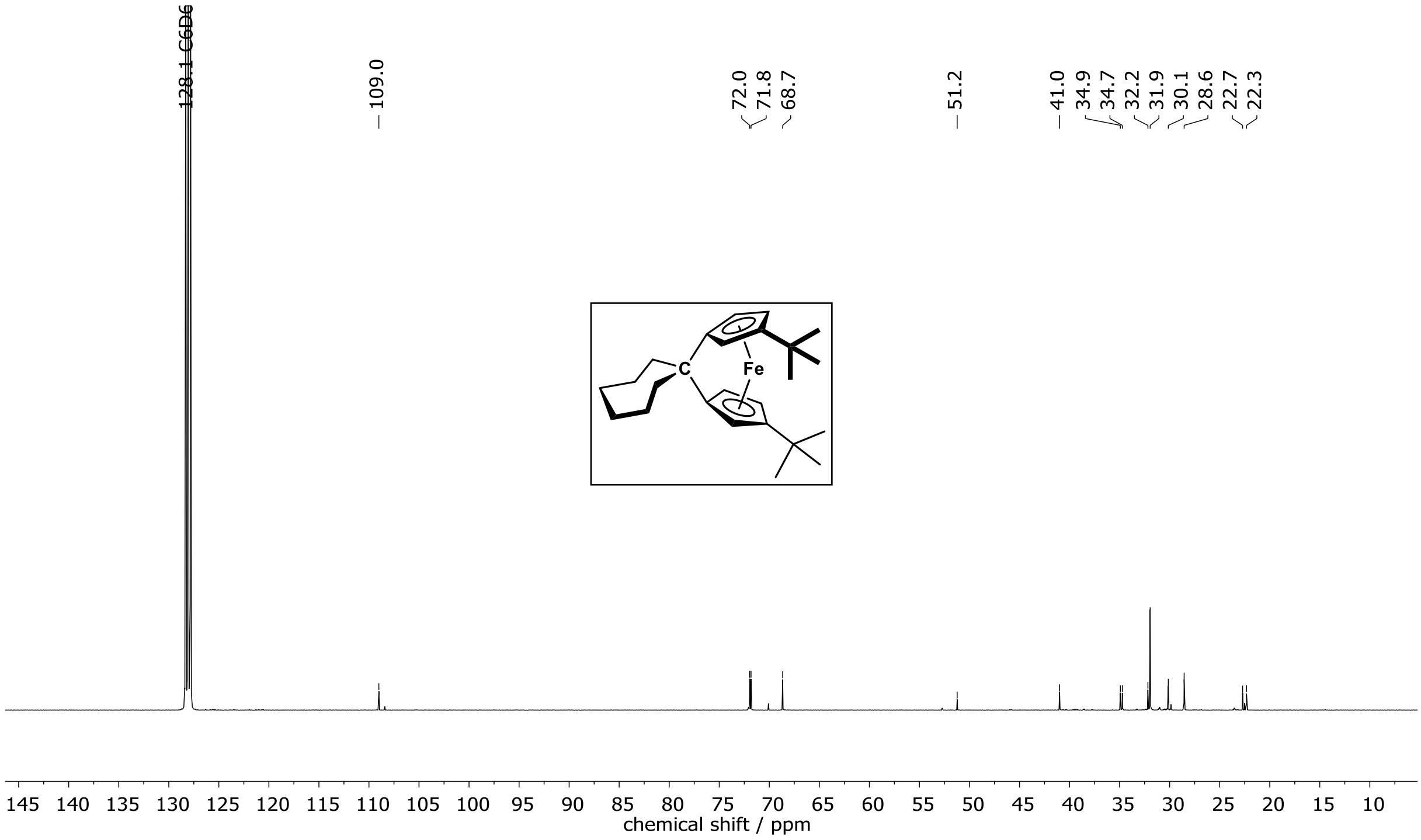

Figure S12: $^{13}C\{^1H\}$ NMR spectrum (100.62 MHz, C_6D_6, 294 K) of the *syn* isomer of **3**.

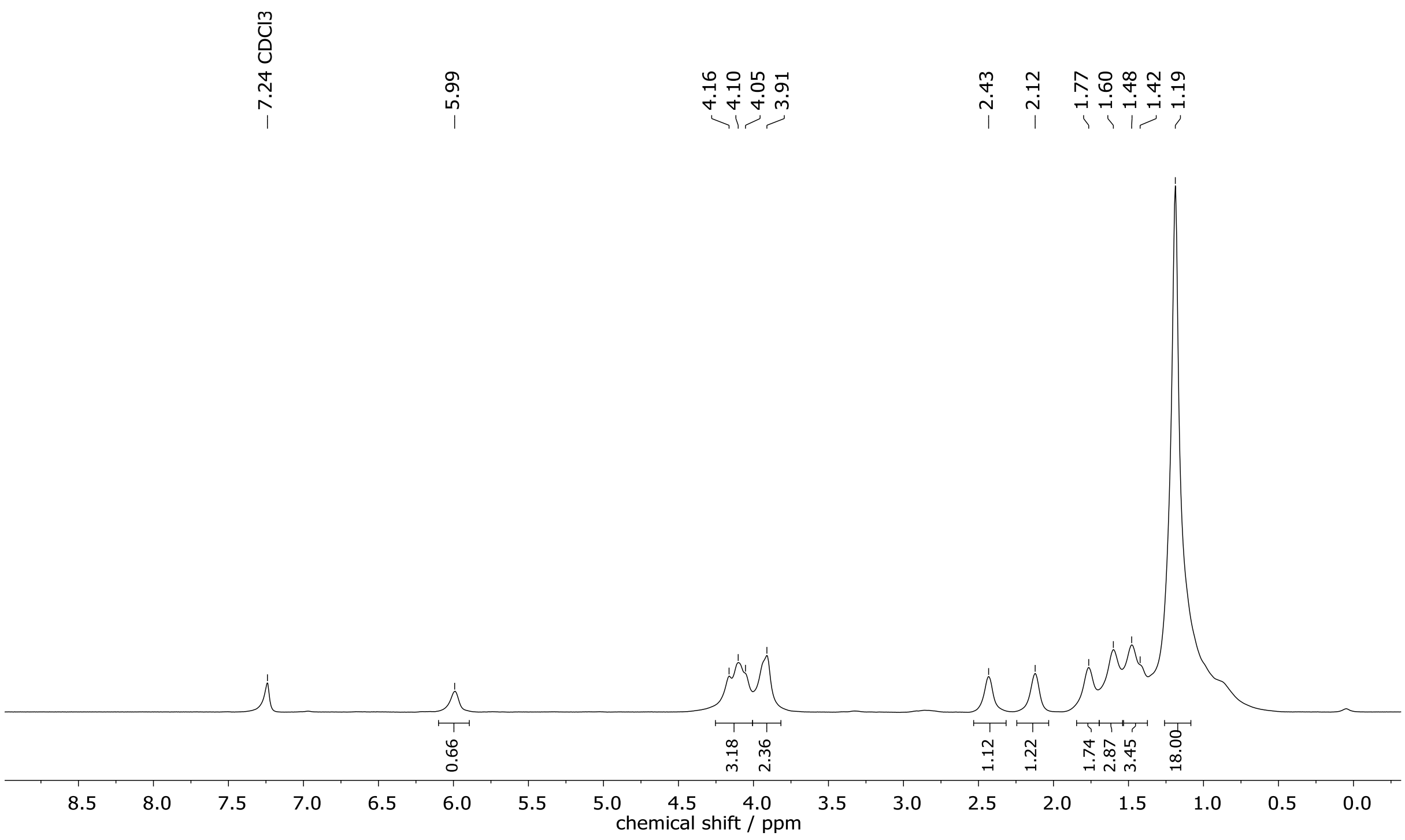

Figure S13: ^1H NMR spectrum (400.13 MHz, $CDCl_3$, 294 K) of product **4** obtained by the thermal ring-opening oligomerization.

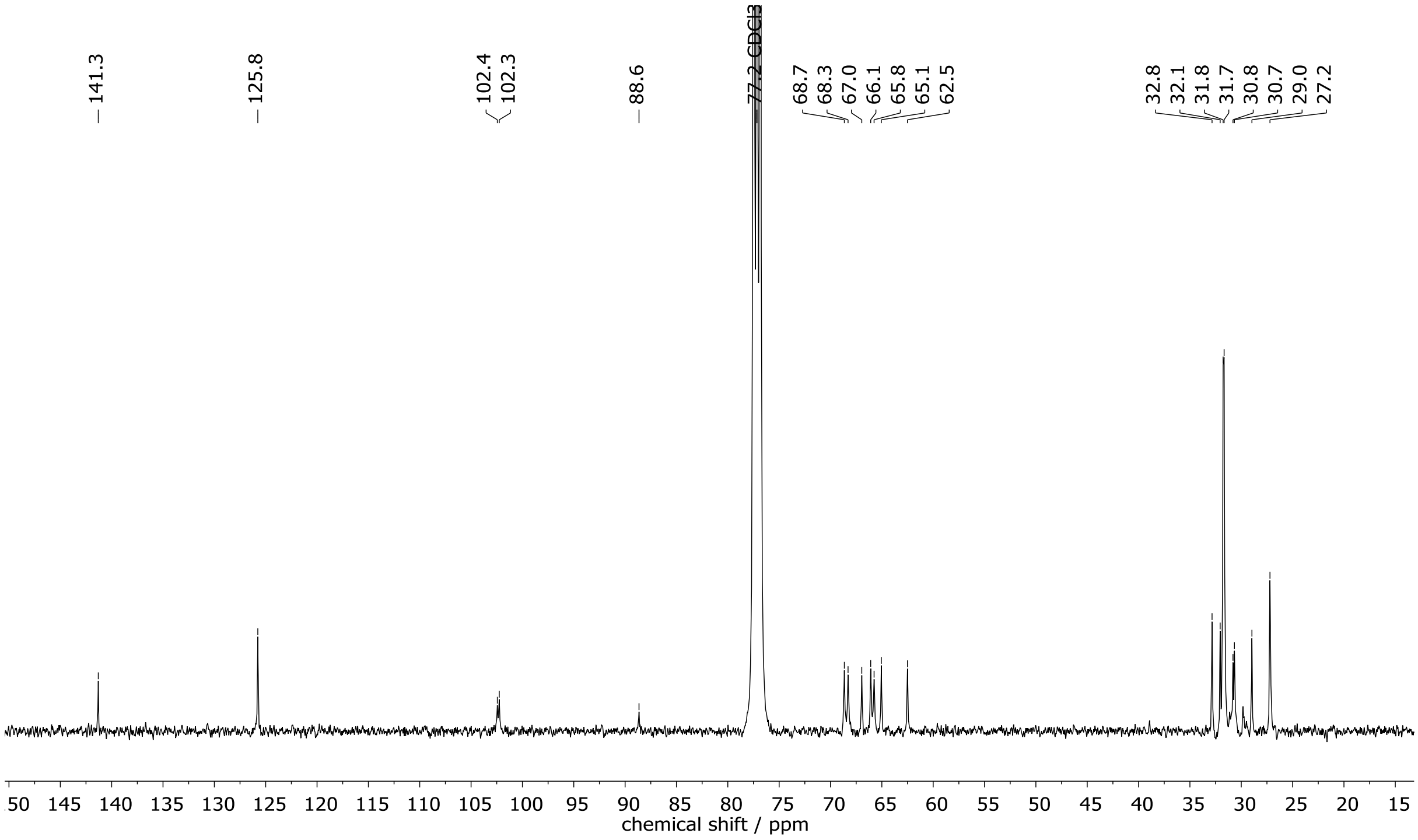

Figure S14: $^{13}C\{^1H\}$ NMR spectrum (100.62 MHz, $CDCl_3$, 294 K) of product **4** obtained by the thermal ring-opening oligomerization.

3. SC-XRD data

The data sets of C[1]magnesocenophane, **2**·dme, and C[1]ferrocenophane, **3**, were collected using a Rigaku XtaLAB Synergy-S diffractometer with a microfocus sealed tube and a HyPix-6000HE Hybrid Photon Counting (HPC) detector. Monochromated $Mo_{K\alpha}$ radiation (λ = 0.71073 Å) was used. Data was collected at 130(2) K and corrected for absorption effects using the multi-scan method. The structures were solved using SHELXT[95] and were refined by full matrix least squares calculations on F^2 (SHELXL2019[96]) in the graphical user interface Shelxle[97].

All crystal structures have been deposited with the Cambridge Crystallographic Data Centre (CCDC) and are available free of charge from the Cambridge Structural Database (CSD).

Crystal structure and refinement data of 2·dme:

CCDC number	2504718	
Empirical formula	$C_{29}H_{46}MgO_2$	
Formula weight	450.97	
Temperature	130(2) K	
Wavelength	0.71073 Å	
Crystal system	monoclinic	
Space group	$P2_1/c$	
Unit cell dimensions	a = 9.8443(5) Å	α = 90°
	b = 15.4990(11) Å	β = 90.824(5)°
	c = 17.1990(10) Å	γ = 90°
Volume	2623.9(3) $Å^3$	
Z	4	
Density (calculated)	1.142 mg m^{-3}	
Absorption coefficient	0.090 mm^{-1}	
F(000)	992	
Crystal size	0.160 x 0.140 x 0.020 mm^3	
θ range for data collection	2.369 to 25.021°	
Index ranges	$-11 \leq h \leq 11$; $-17 \leq k \leq 18$; $-20 \leq l \leq 20$	
Reflections collected	16291	
Independent reflections	4636 [R(int) = 0.0801]	
Completeness to θ = 25.021°	100.0 %	
Absorption correction	semi-empirical from equivalents	
Max. and min. transmission	1.0000 and 0.8042	
Refinement method	full-matrix least-squares on F^2	
Data / restraints / parameters	4636 / 232 / 372	
Goodness-of-fit on F^2	1.037	
Final R indices [I>2σ(I)]	R1 = 0.0599, wR2 = 0.1532	
R indices (all data)	R1 = 0.0899, wR2 = 0.1706	
Extinction coefficient	n/a	
Largest diff. peak and hole	0.636 and -0.329 e.$Å^{-3}$	

Crystal structure and refinement data of 3:

CCDC number	2504717	
Empirical formula	$C_{25}H_{36}Fe$	
Formula weight	392.39	
Temperature	130(2) K	
Wavelength	0.71073 Å	
Crystal system	monoclinic	
Space group	$P2_1/n$	
Unit cell dimensions	a = 6.9024(3) Å	α = 90°
	b = 16.3857(5) Å	β = 100.365(3)°
	c = 18.4825(7) Å	γ = 90°
Volume	2056.27(14) $Å^3$	
Z	4	
Density (calculated)	1.267 mg m^{-3}	
Absorption coefficient	0.739 mm^{-1}	
F(000)	848	
Crystal size	0.380 x 0.040 x 0.020 mm^3	
θ range for data collection	2.240 to 27.937°	
Index ranges	$-9 \le h \le 9$, $-21 \le k \le 21$, $-23 \le l \le 24$	
Reflections collected	32289	
Independent reflections	4900 [R(int) = 0.0405]	
Completeness to θ = 25.021°	100.0 %	
Absorption correction	semi-empirical from equivalents	
Max. and min. transmission	1.00000 and 0.55829	
Refinement method	full-matrix least-squares on F^2	
Data / restraints / parameters	4900 / 6 / 269	
Goodness-of-fit on F^2	1.022	
Final R indices [I>2σ(I)]	R1 = 0.0288, wR2 = 0.0665	
R indices (all data)	R1 = 0.0358, wR2 = 0.0690	
Extinction coefficient	n/a	
Largest diff. peak and hole	0.427 and -0.277 e.$Å^{-3}$	

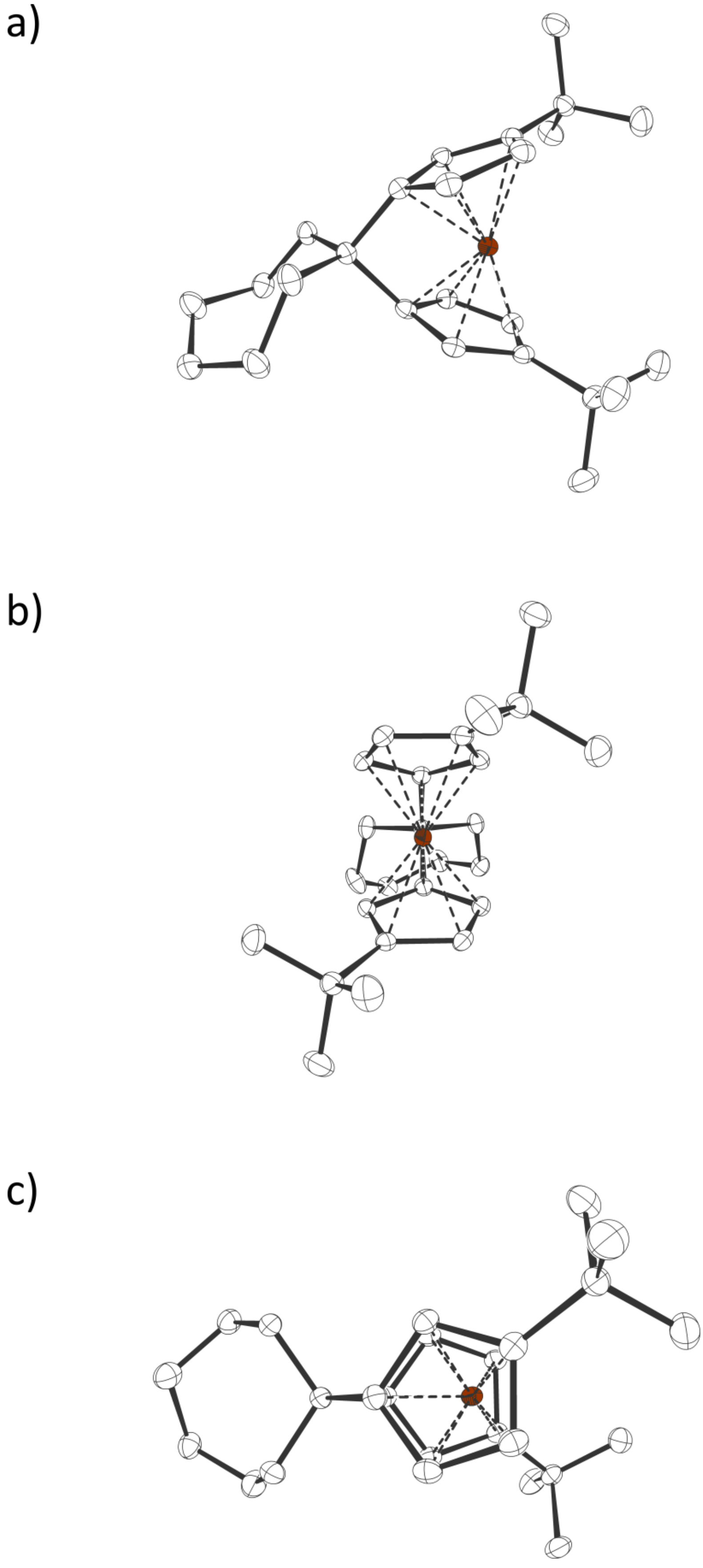

Figure S15: Molecular structure of **3** in the crystal; a) side view, b) front view, c) top view (displacement ellipsoids drawn at 50 % probability level; H-atoms omitted for clarity).

4. HR-MS data

High-resolution mass spectrometry was performed using a Bruker solariX 7T FT-ICR-MS (Massachusetts, USA) equipped with an electrospray ion source and a syringe pump with a flow rate of 10 µl min^{-1} for sample injection. The ions generated in the positive ion mode were accumulated in the hexapole region for 0.05 s and transferred subsequently into the ion cyclotron resonance (ICR) cell. For gentle desolvation, the drying gas temperature was set to 200 °C, the capillary voltage to 4500 V and the capillary exit voltage to 200 V.

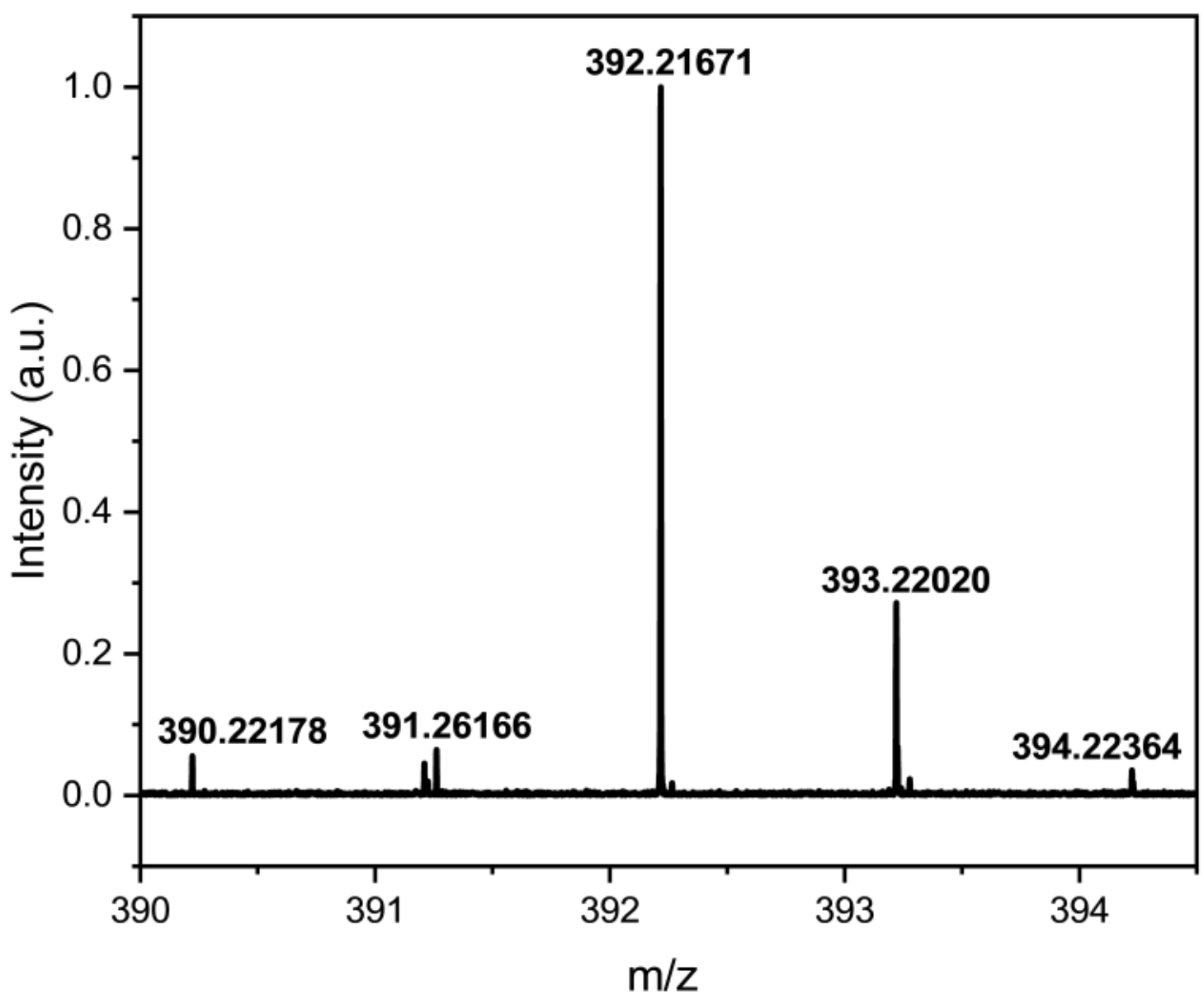

Figure S16: High-resolution mass spectrum of **3**.

5. TGA and DSC data

Thermogravimetric analysis (TGA) was done on a Netzsch TGA 209 Libra in a range of 30 °C to 590 °C, including a drying step with a heating rate of 5 K min^{-1} and in a nitrogen atmosphere. Differential scanning calorimetry (DSC) measurements were carried out with Netzsch DSC 214 in a range from 25 °C to 580 °C with a heating rate of 10 K min^{-1} and in a nitrogen atmosphere (for **3**); in a range of -80 °C to 120 °C with a heating of 5 K min^{-1} and in nitrogen atmosphere (for **4**). For TGA and DSC, the recorded data were evaluated using NETSCH Proteus-Thermal Analysis (version 8.0.3).

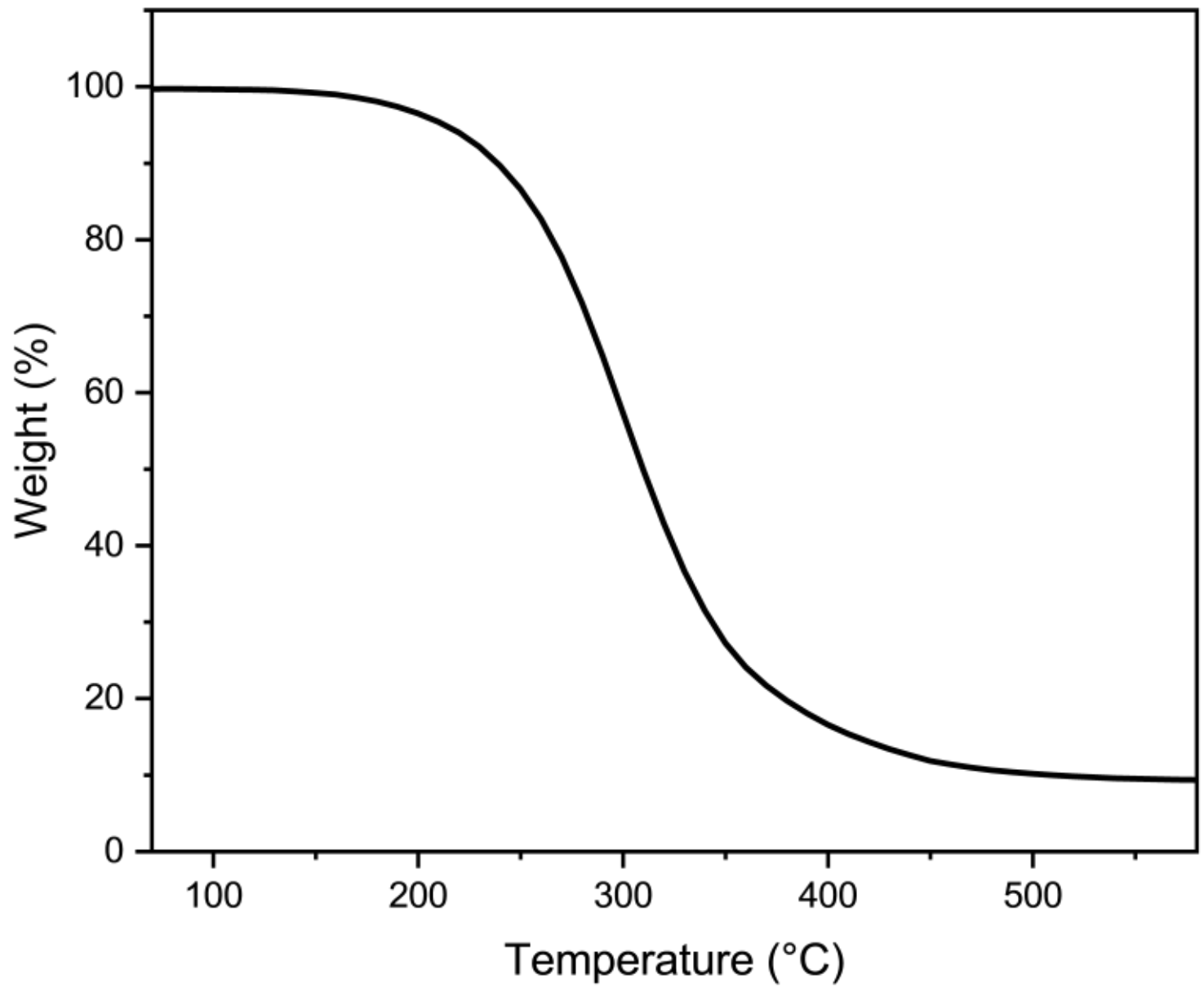

Figure S17: TGA curve of **4** using a heating rate of 20 K min^{-1} from 70 °C to 580 °C in a nitrogen atmosphere.

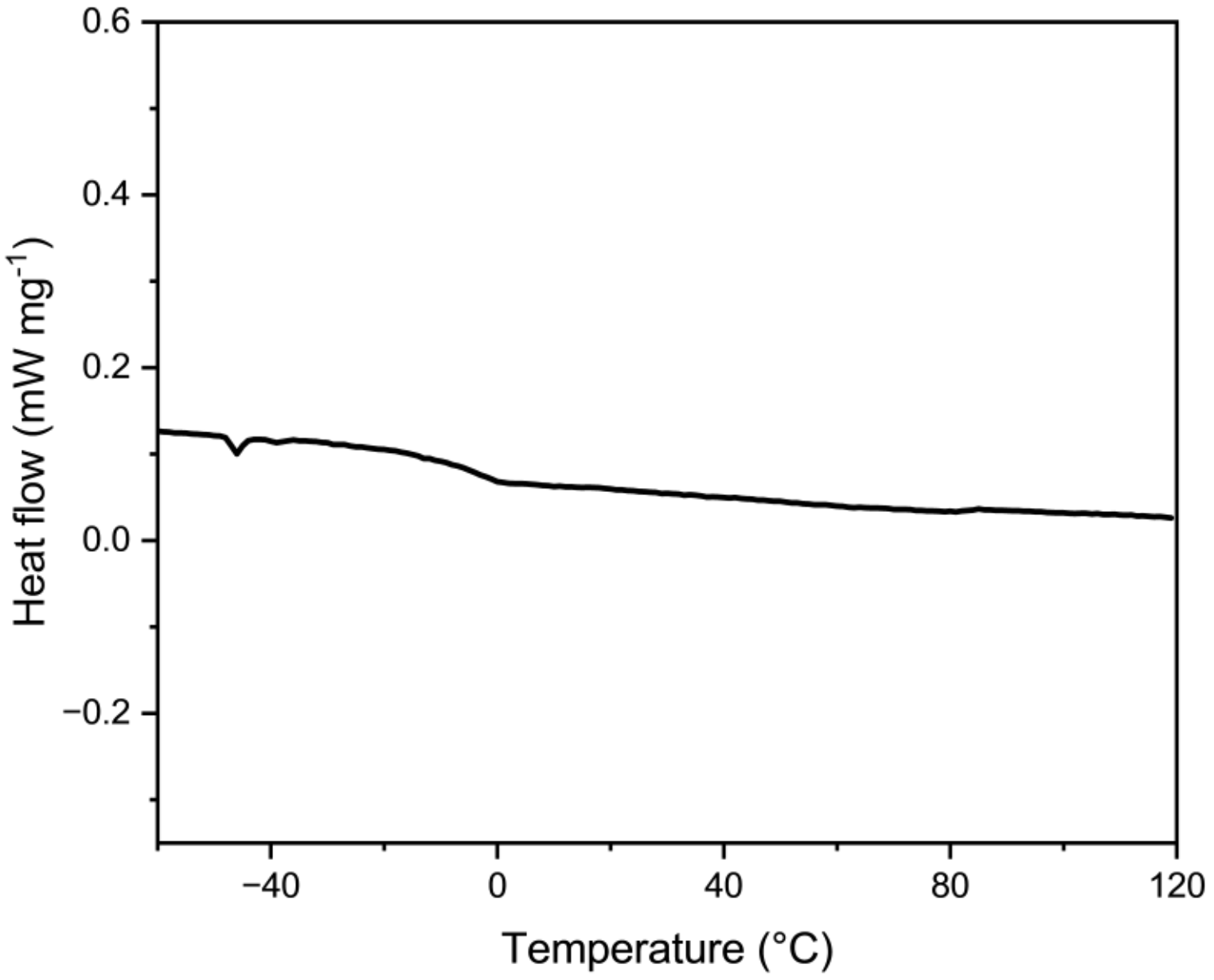

Figure S18: DSC curve of **4** using a heating rate of 5 K min^{-1} from -80 °C to 120 °C in a nitrogen atmosphere.

6. GPC data

Gel permeation chromatography (GPC) was performed with a system composed of a 1260 Infinity II (Agilent Technologies), an UV detector at 260 nm and an RI detector at 35°, with thf as the mobile phase (flow 82 rate 1 ml min^{-1}) on a SDV column set from polymer standard service (PSS) (SDV 10^3 Å, SDV 10^5 Å, and SDV 10^6 Å). Calibration was carried out using PS standards from PSS.

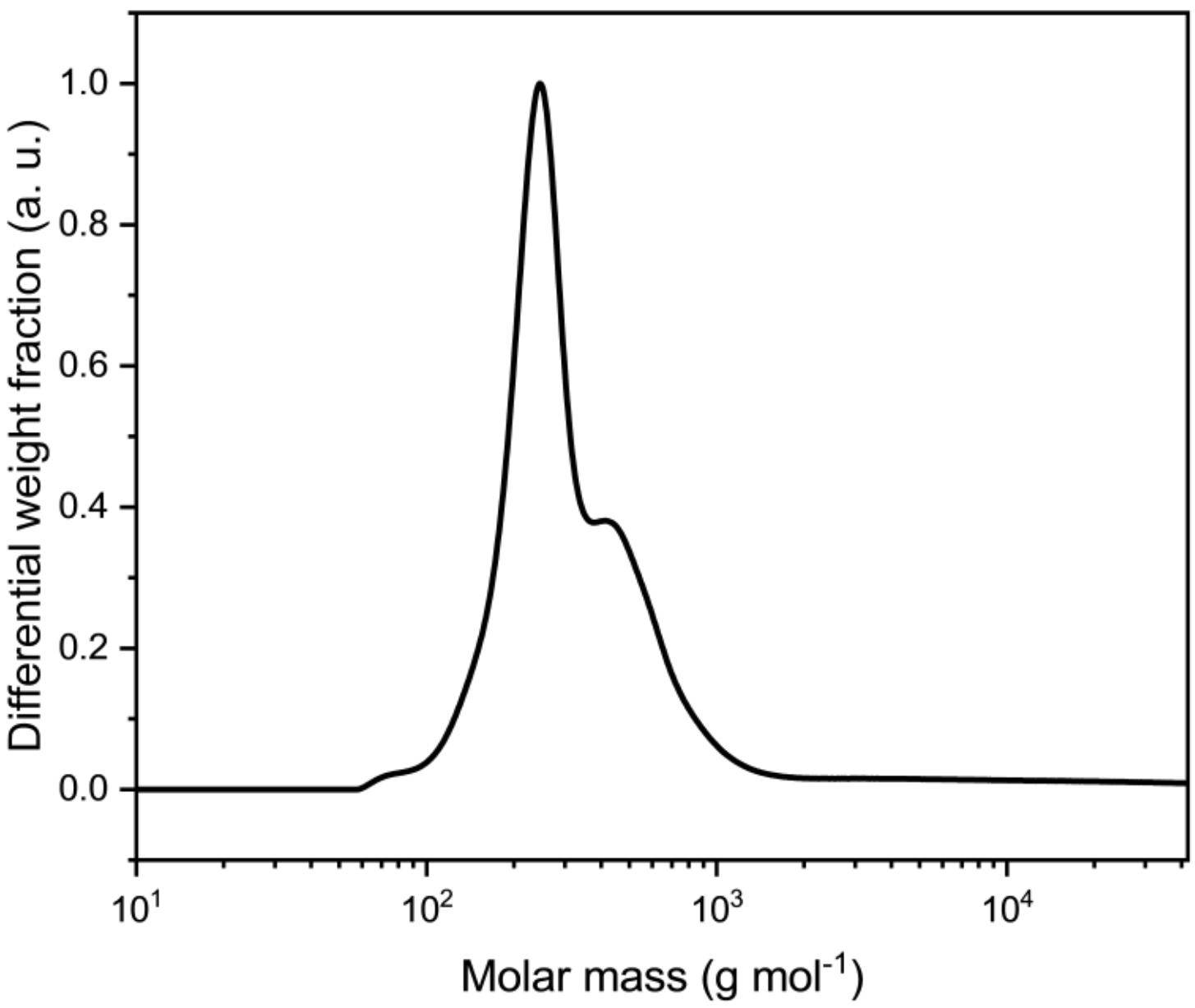

Figure S19: Molar mass distribution of **4** obtained by GPC measurement in thf *vs.* PS standards.

7. MALDI-ToF MS data

MALDI-ToF mass spectra were recorded on a Bruker Daltonik Autoflex speed TOF/TOF mass spectrometer. 2-[3-(4-*tert*-butylphenyl)-2-methyl-2-propenylidene]malononitrile (DCTB) and AgTFA were used as the matrix.

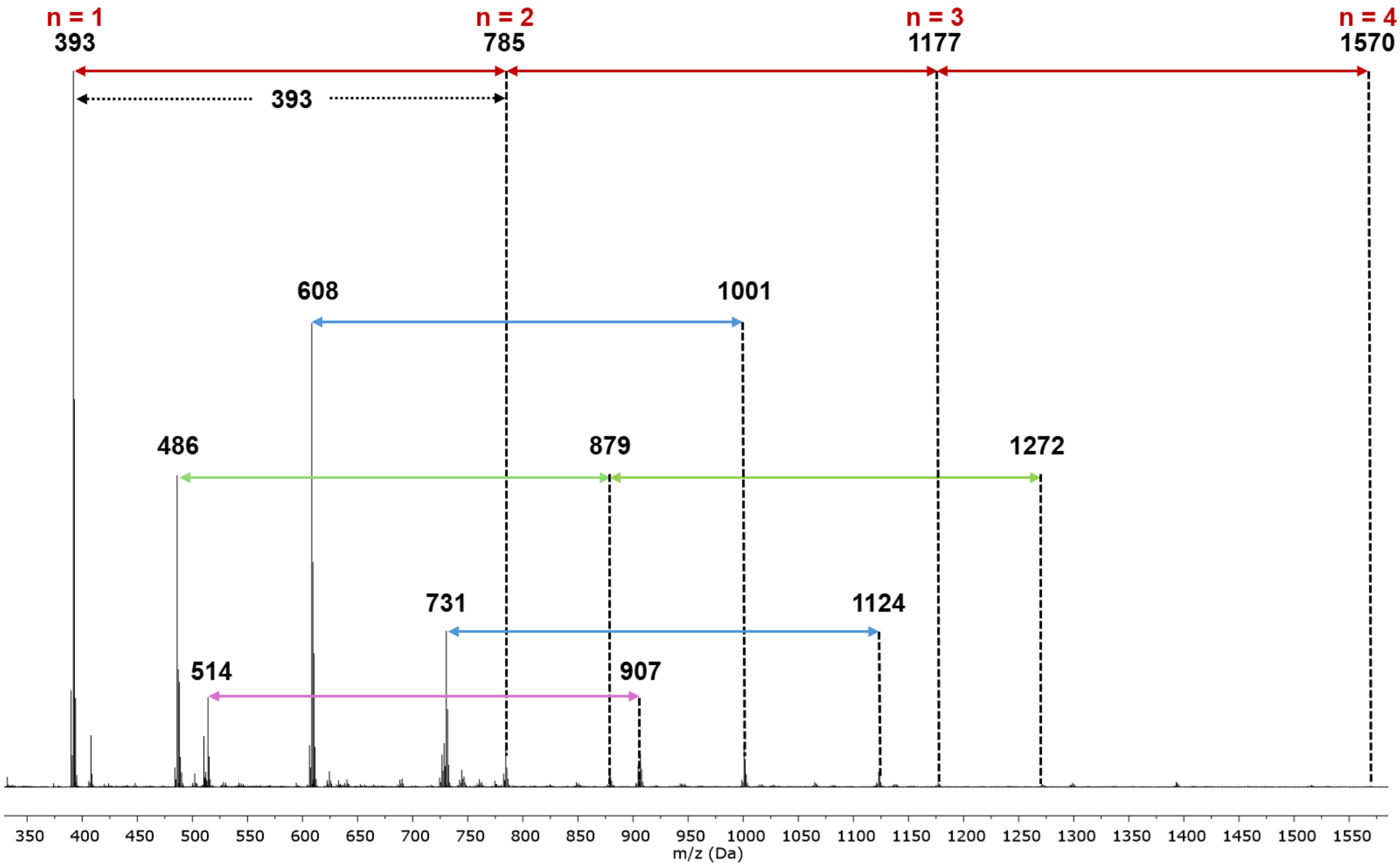

Figure S20: MALDI-ToF mass spectrum of **4** (repeat unit: 393 Da).

8. UV-vis data

UV-vis spectra were obtained on a Perkin Elmer-Lambda 750 spectrometer employing standard quartz cells (0.01 m) from 200 to 800 nm with a 1 nm increment and an integration time of 0.2 s.

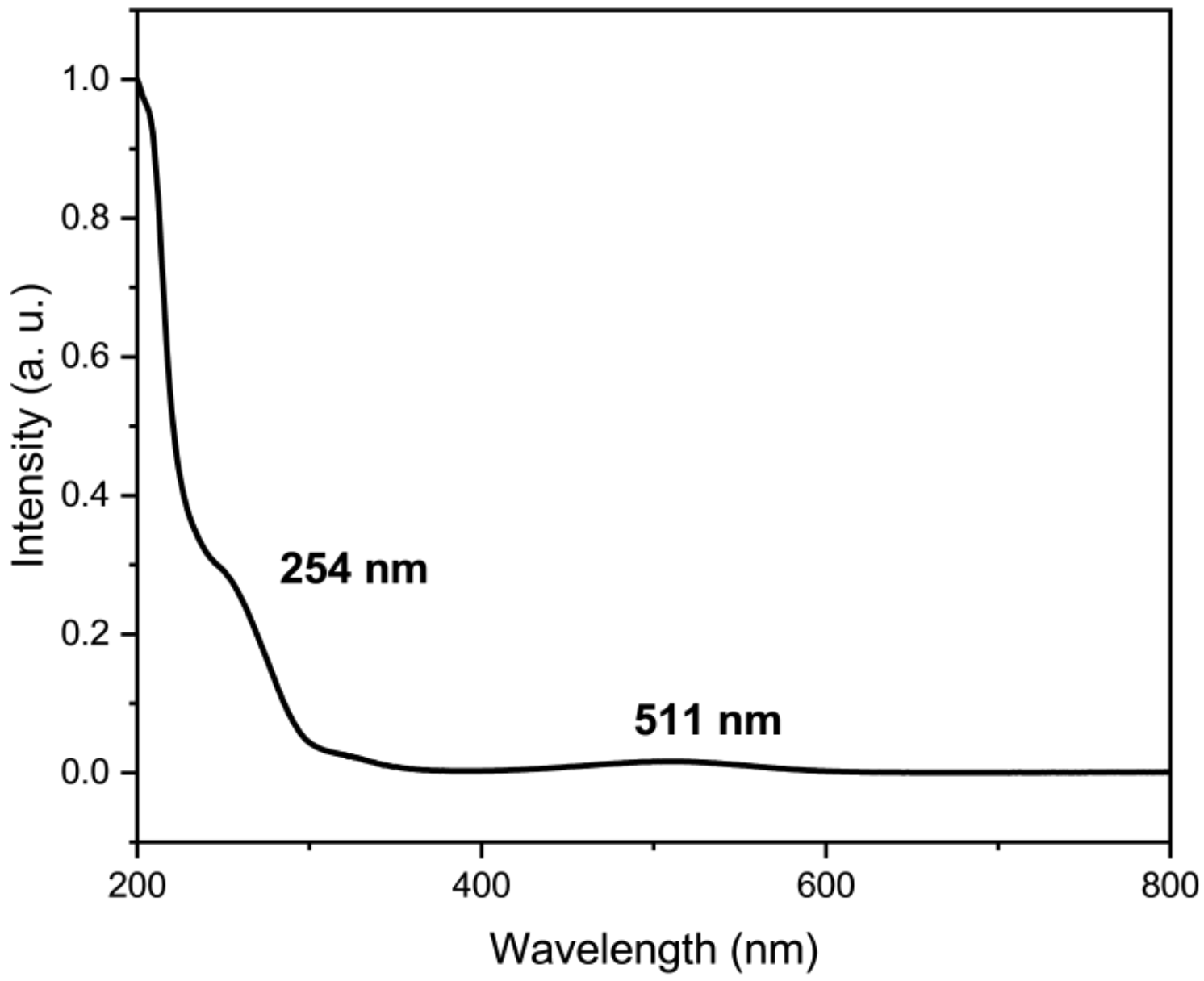

Figure S21: Normalized UV-vis spectrum of **3** in hexane in a range of 200 to 800 nm.

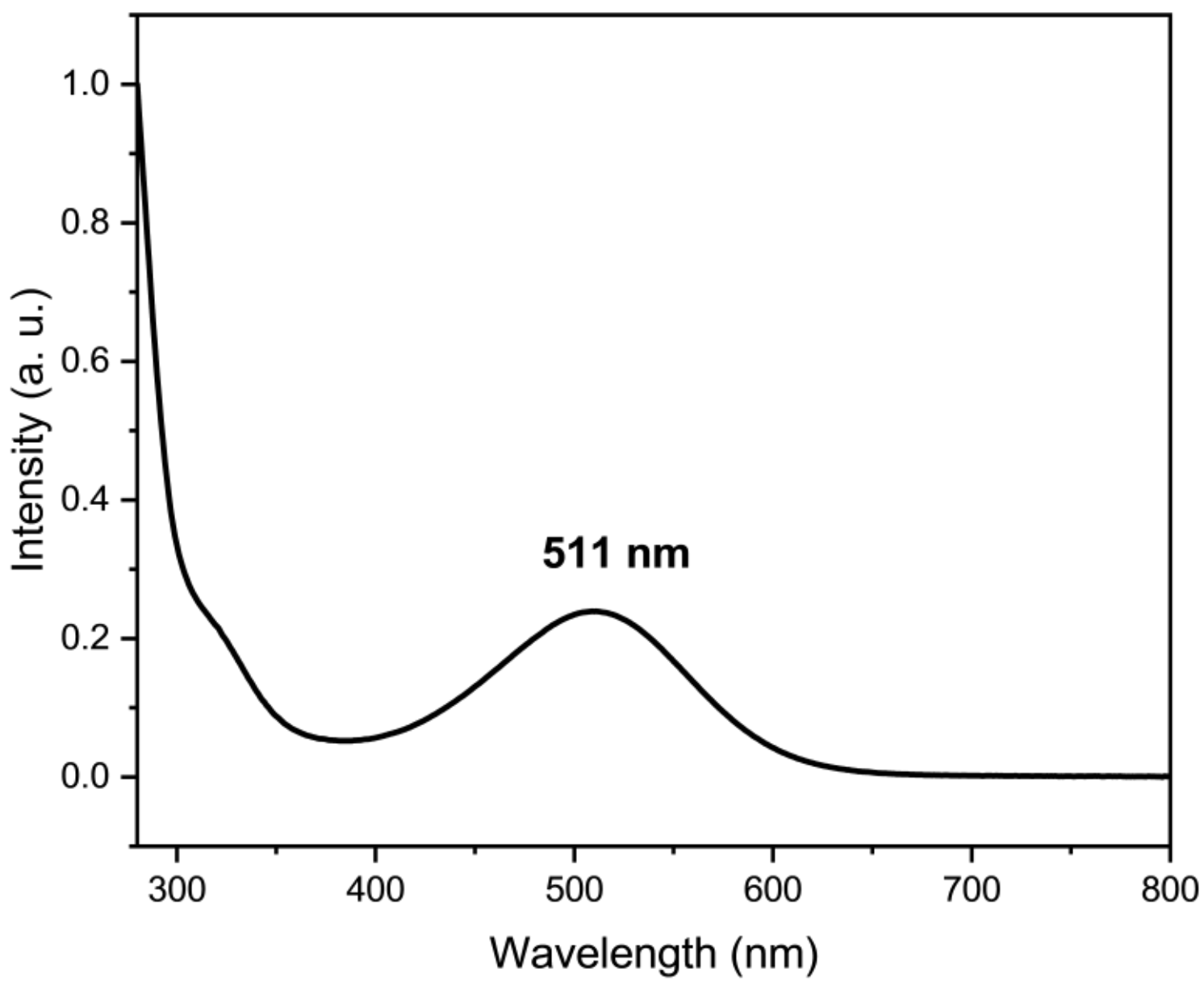

Figure S22: Normalized UV-vis spectrum of **3** in hexane in a range of 280 to 800 nm, highlighting the lowest energy absorbance at 511 nm.

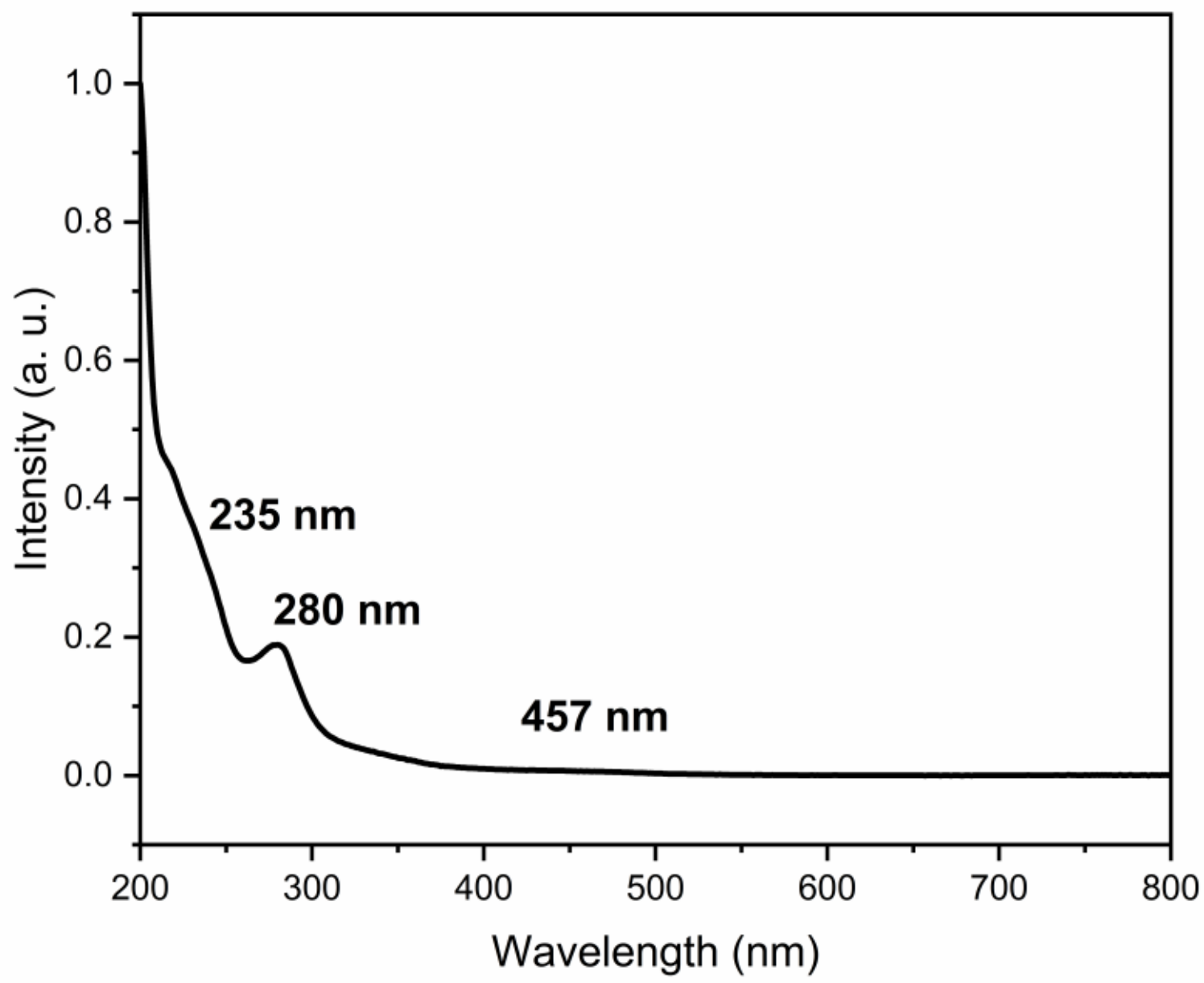

Figure S23: Normalized UV-vis spectrum of **4** in hexane in a range of 200 to 800 nm.

9. CV data

CV measurements were recorded with an EmStat^{3+} Pico blue potentiostat (PalmSens) in a nitrogen filled glovebox, a glassy carbon working electrode, platinum wire as counter electrode, a non-aqueous Ag/Ag^{+} pseudo reference electrode, as well as [*n*-Bu$_4$][PF$_6$] (0.1 M) in *o*-DFB (refluxed over CaH_2 for at least 2 d distilled and stored over activated 3 Å molecular sieves) as supporting electrolyte. After the initial measurements, ferrocene was added as internal standard, and the voltammograms were corrected by positioning the redox potential of ferrocene to 0 V. The data was processed with the PSTrace software package (PalmSens).

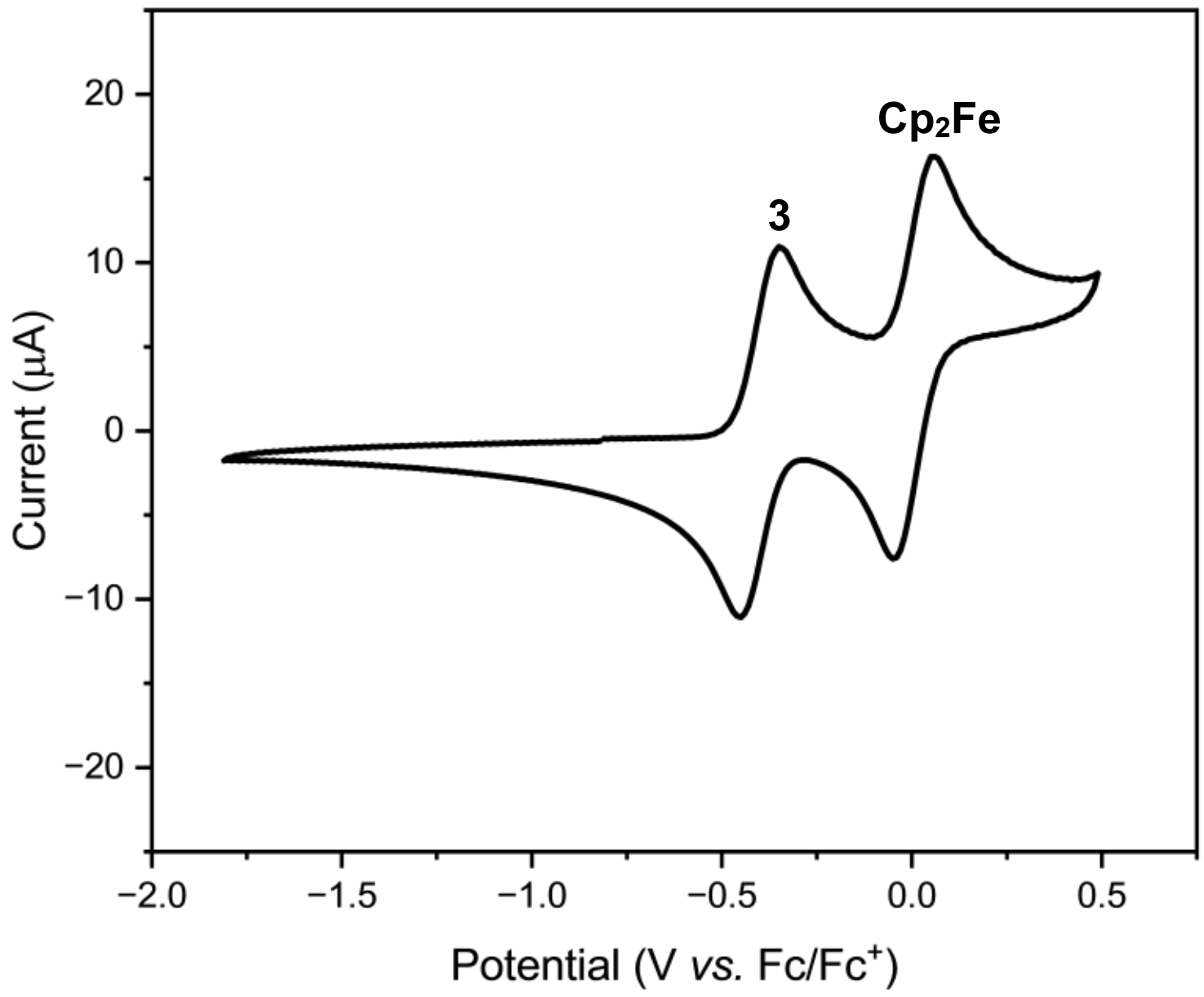

Figure S24: Cyclic voltammetry measurement of **3** in the presence of ferrocene (Cp_2Fe) in a potential range of -2.00 to 0.75 V *vs*. Fc/Fc^{+} and a scan rate of 1.00 V s^{-1} using tetrabutylammonium hexafluorophosphate (0.1 M in 1,2-difluorobenzene) as supporting electrolyte.

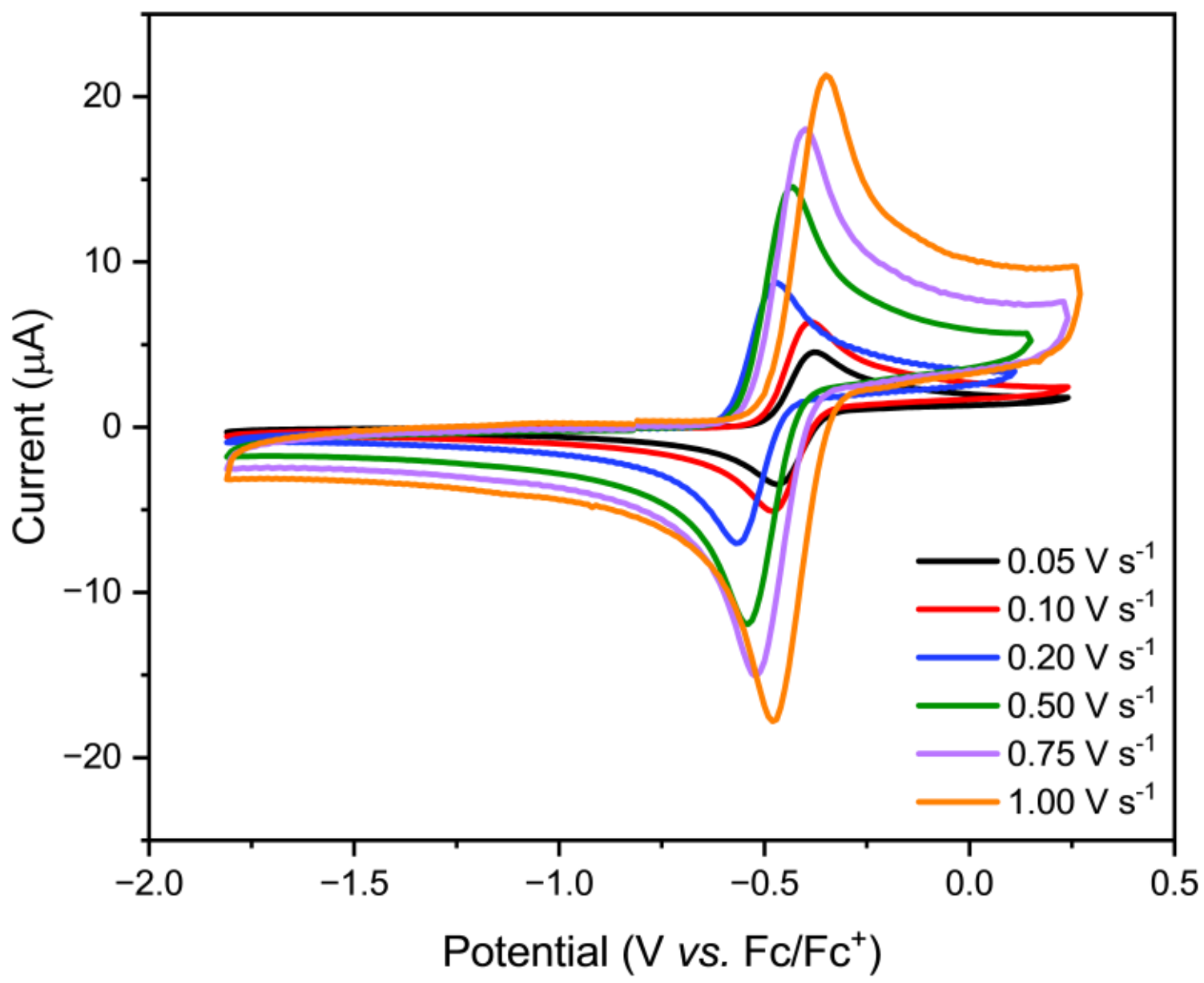

Figure S25: Cyclic voltammetry measurements of **3** in a potential range of -2.00 to 0.50 V *vs*. Fc/Fc^+ at different scan rates using tetrabutylammonium hexafluorophosphate (0.1 M in 1,2-difluorobenzene) as a supporting electrolyte.

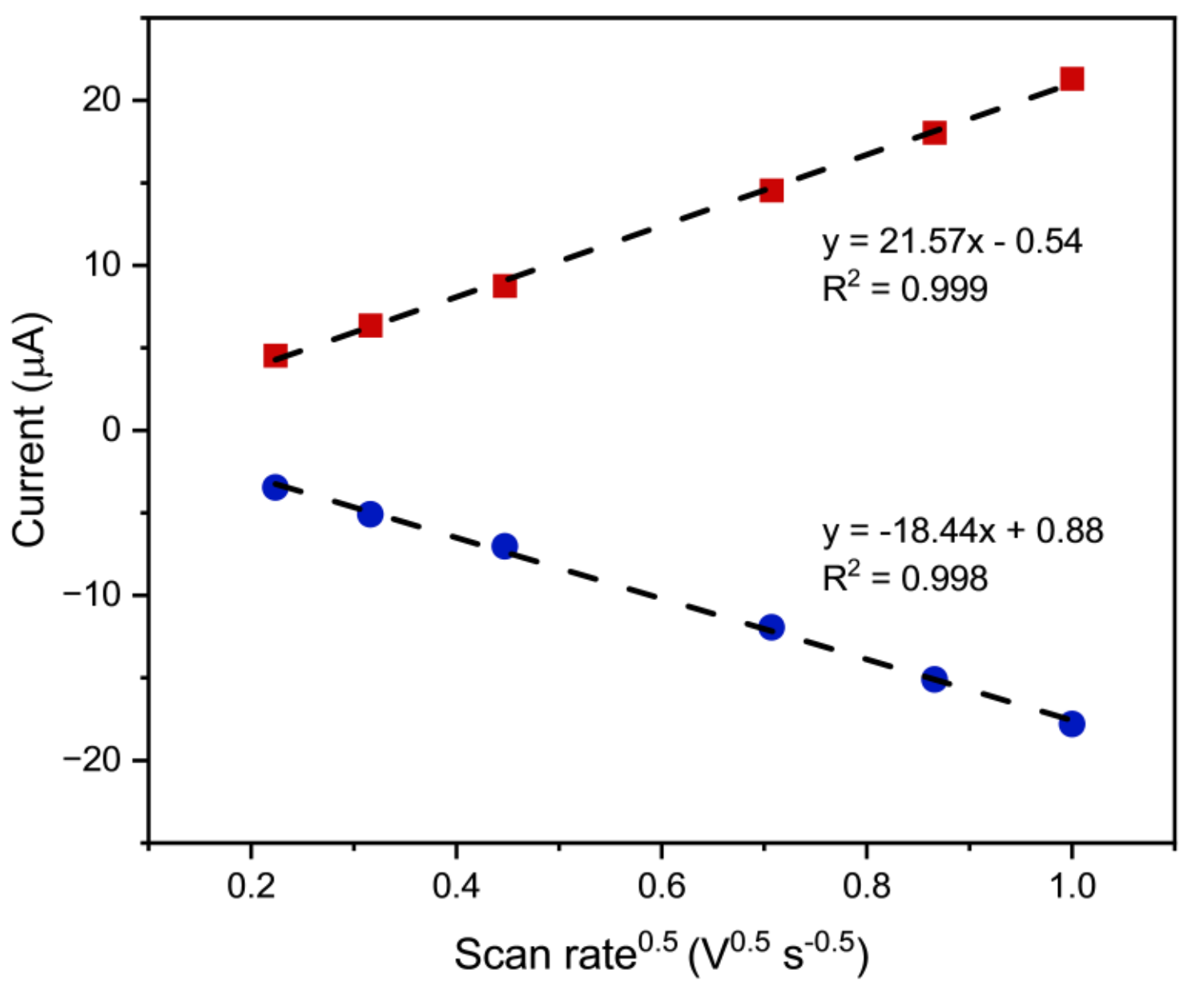

Figure S26: RANDLES–ŠEVČÍK analysis, which indicates reversible behavior of **3**.

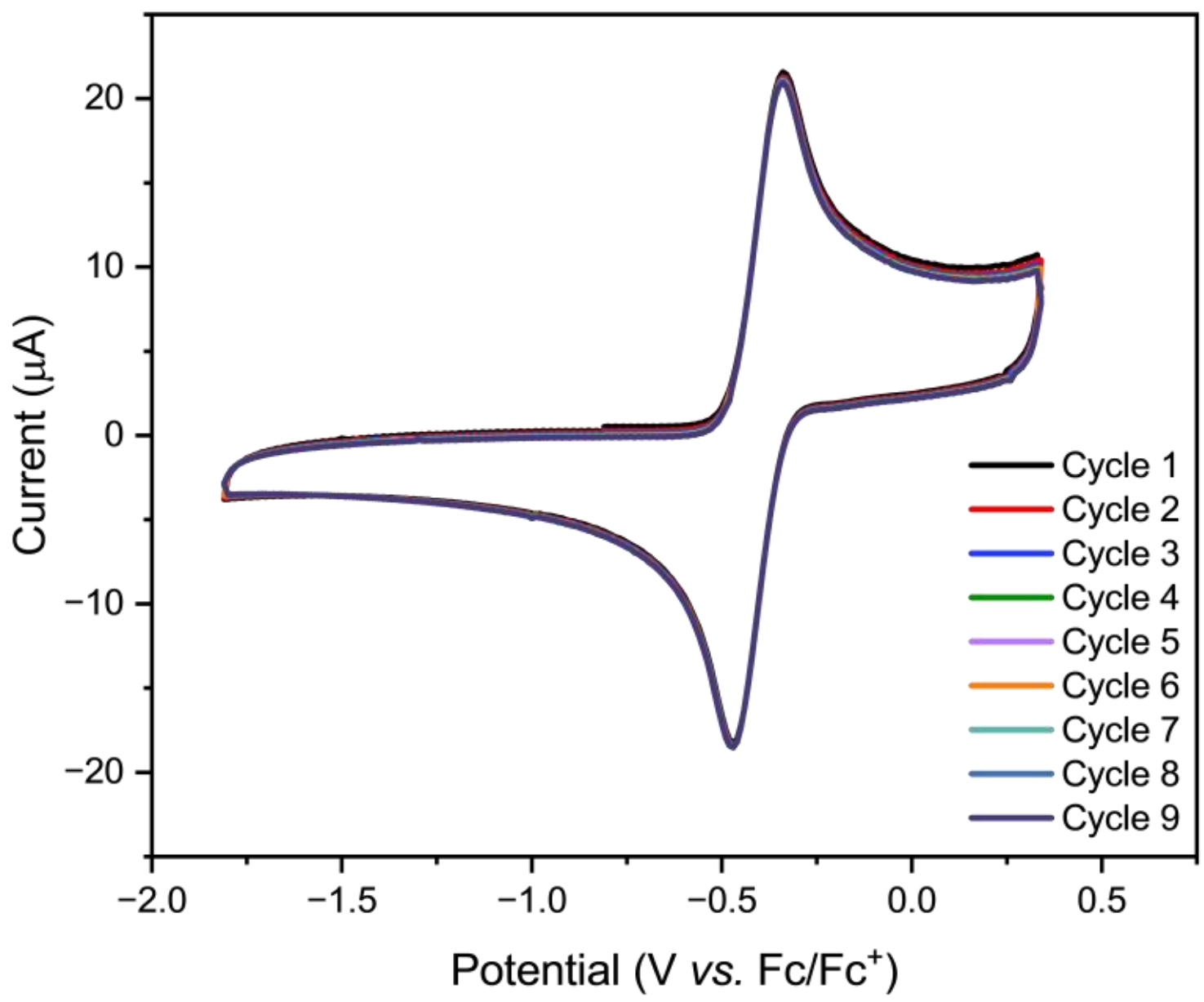

Figure S27: Cyclic voltammetry measurements of **3** in a potential range of -2.00 to 0.75 V *vs*. Fc/Fc^{+} and a scan rate of 1.00 V s^{-1} using tetrabutylammonium hexafluorophosphate (0.1 M in 1,2-difluorobenzene) as a supporting electrolyte.

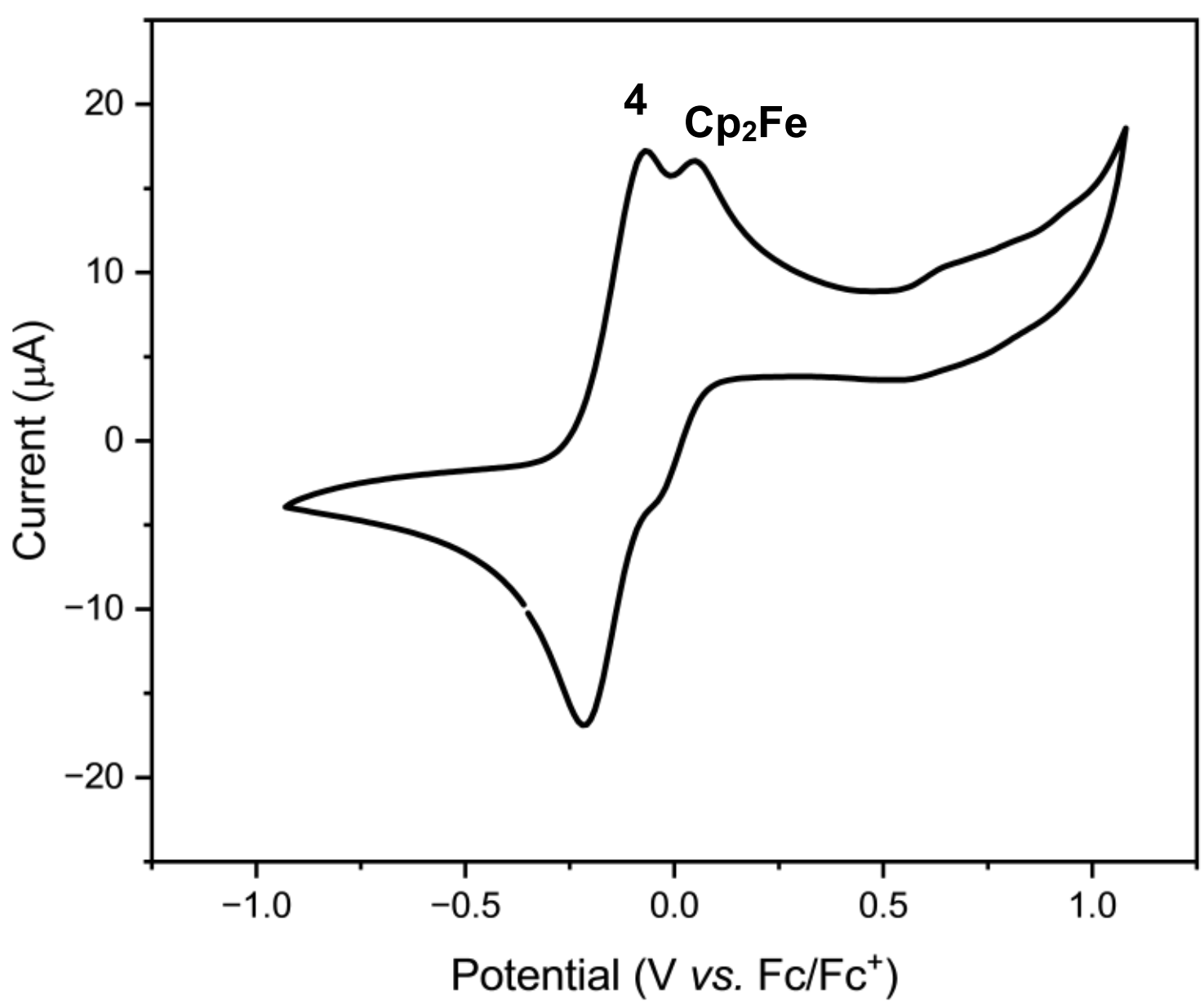

Figure S28: Cyclic voltammetry measurement of **4** in the presence of ferrocene (Cp_2Fe) in a potential range of -1.25 to 1.25 V *vs*. Fc/Fc^{+} and a scan rate of 0.10 V s^{-1} using tetrabutylammonium hexafluorophosphate (0.1 M in 1,2-difluorobenzene) as supporting electrolyte.

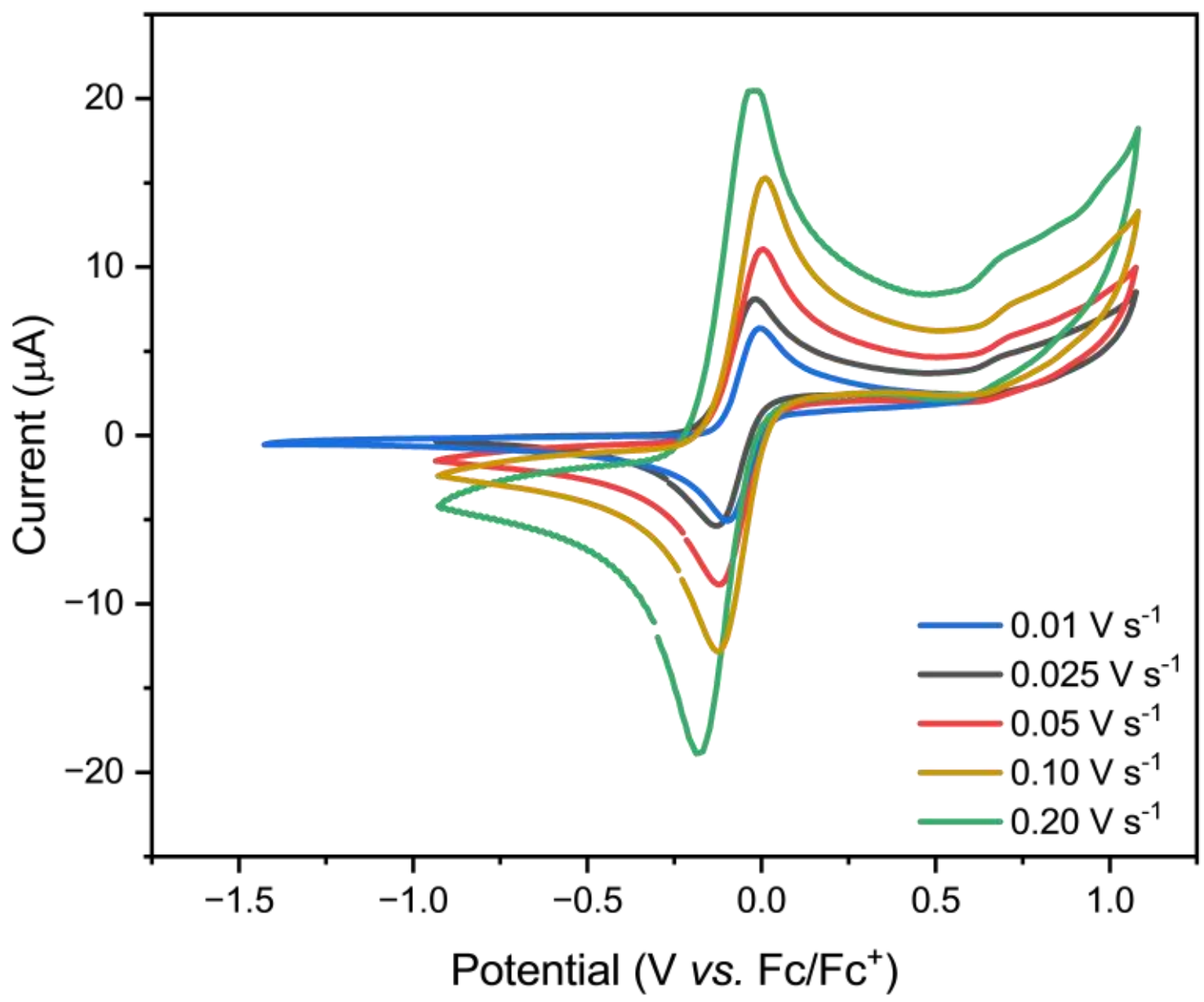

Figure S29: Cyclic voltammetry measurements of **4** in a potential range of -1.75 to 1.25 V *vs*. Fc/Fc^{+} at different scan rates using tetrabutylammonium hexafluorophosphate (0.1 M in 1,2-difluorobenzene) as a supporting electrolyte.

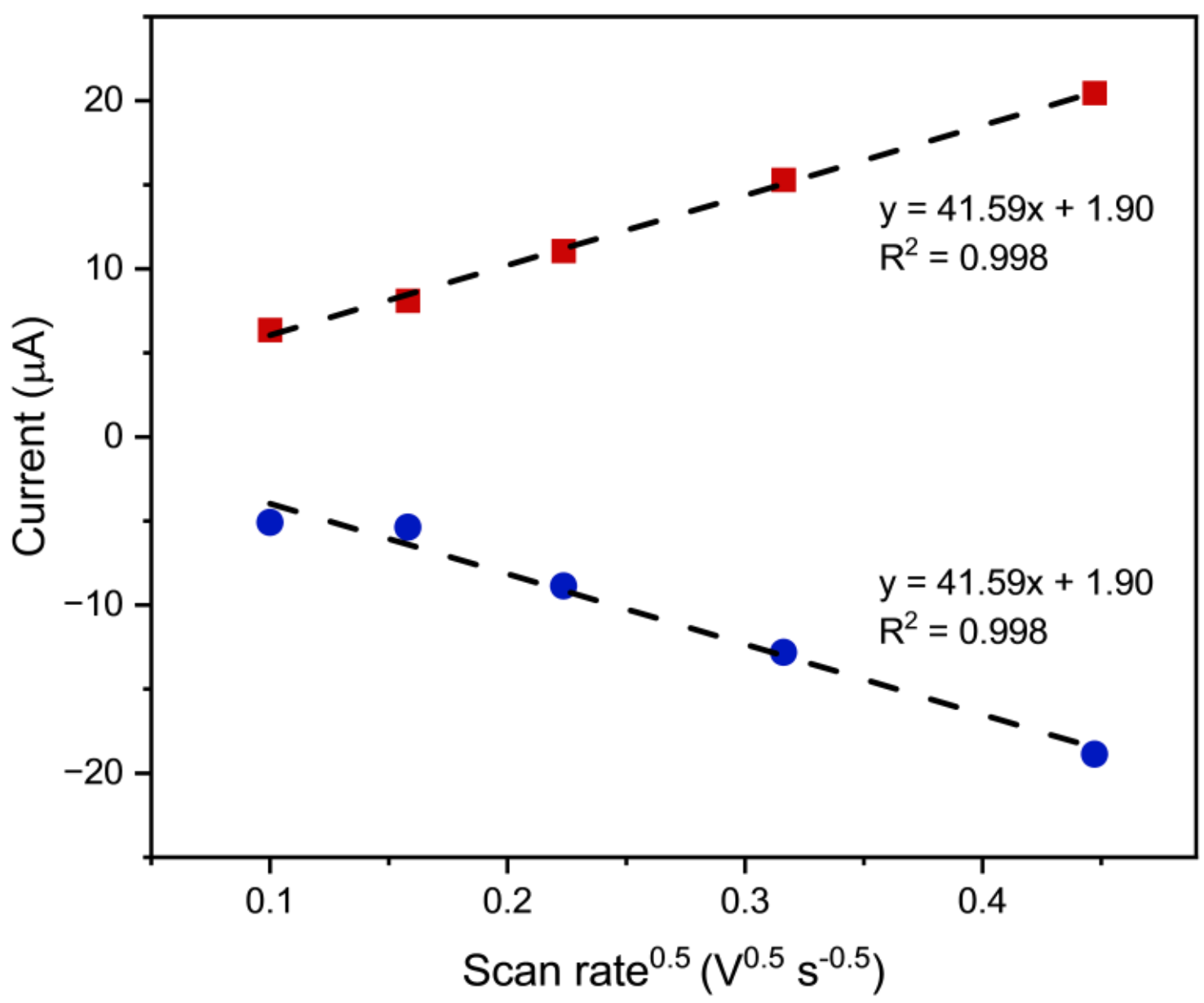

Figure S30: RANDLES–ŠEVČÍK analysis, which indicates reversible behavior of **4**.

10.Computational details

10.1 General details

Geometry optimizations were performed using the Gaussian 16, Revision C.01 software suite.[98] Geometry optimizations for the ring-strain calculations were computed using the B3PW91[99,100] density functional, in combination with the 6-311+G(d,p)[101-105] basis set. Geometry optimizations for the bonding analysis were carried out with the B3PW91 density functional, in combination with Grimme's third generation dispersion correction terms D3[106] and the Becke-Johnson damping function (BJ)[107,108], and the def2-SVP[109] basis set. Stationary points were verified to be minima on the potential energy surface by a subsequent frequency analysis (zero imaginary frequencies). Partial Charges, Bond Orders (BO) and Effective Oxidation States (EOS)[110] were conducted using the Topological Fuzzy Voronoi Cells (TFVC)[111] atomic definition, and 40 x 140 atomic grid for numerical integrations using the APOST3D software suite.[112] The Energy Decomposition Analysis (EDA) was carried out with ADF 2019[113] at the B3LYP(ZORA)/TZ2P[114] level of theory. Non-Covalent Interactions (NCI) were calculated with the NCI plot 4.0. Orbital pairwise decompositions were computed using the o-NCI software[89] and Pipek-Mezey localized orbitals obtained from the Gaussian 16, Revision C.01 software suite.

10.2 Ring-strain calculations

The ring-strain in C[1]ferrocenophanes has been calculated by adapting Müller's method[72], considering the isodesmic reaction of the respective C[1]ferrocenophane with ferrocene to give the respective dimer based on the mechanistic assumption of Fe–Cp bond cleavage.

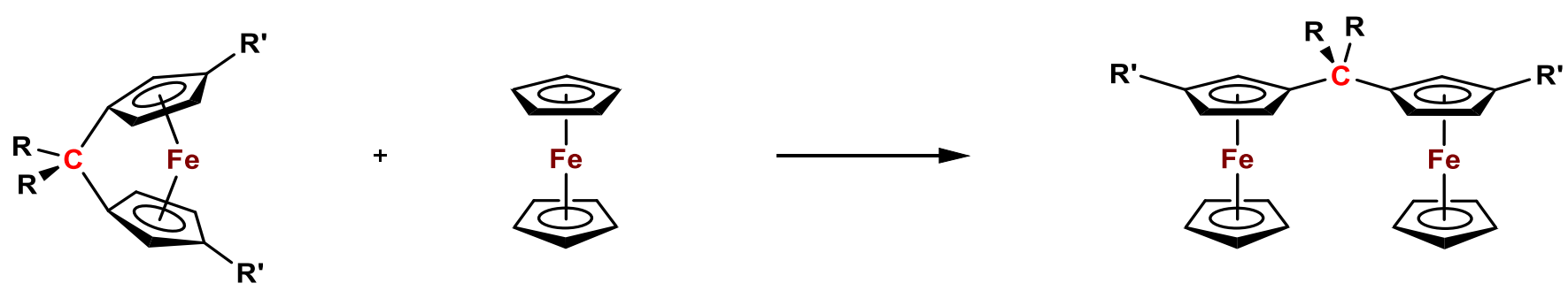

Figure S31: Isodesmic reaction considered for the calculations of ring-strain energies of C[1]ferrocenophanes.

10.3 Bonding Analysis

Table S1: Selected structural parameters of optimized [1]ferrocenophanes, calculated at the B3PW91-D3(BJ)/def2-SVP level of theory.

	Fe–Cp^{cent} (pm)	α (°)
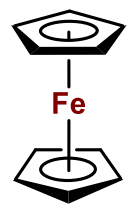	163.5	0
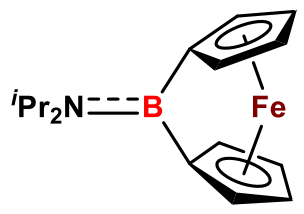	162.3 / 162.4	30.1
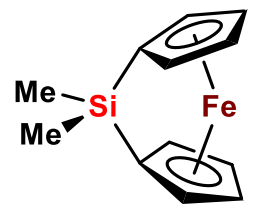	162.9	19.2
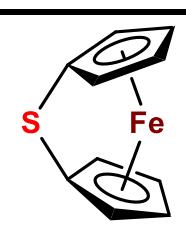	162.5	30.9
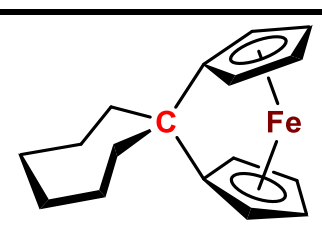	162.3 / 162.4	37.4
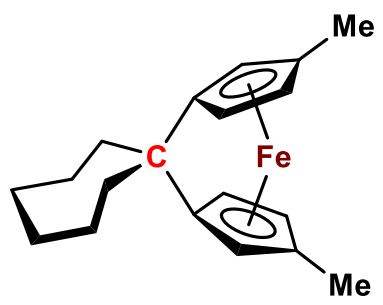	161.8 / 162.0	36.8
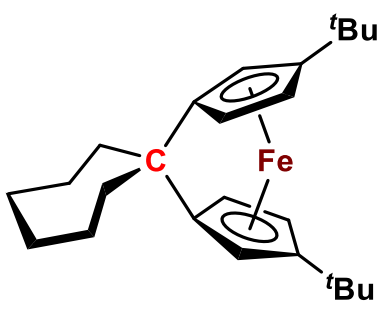	161.4 / 161.6	36.3

Table S2: Partial charges and bond orders using Topological Fuzzy Voronoi Cells AIM definition of different [1]ferrocenophanes, calculated at the B3PW91-D3(BJ)/def2-TZVP//B3PW91-D3(BJ)/def2-SVP level of theory.

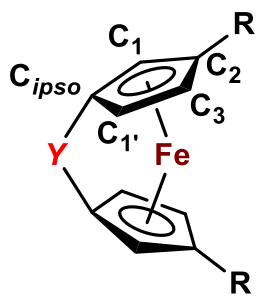	Q (Fe) (a. u.)	Q (L) (a. u.)	BO Fe–C_{ipso}	BO Fe–C_1	BO Fe–$C_{1'}$	BO Fe–C_2	BO Fe–C_3
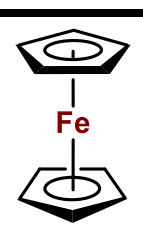	0.98	-0.98	0.49	0.49	0.49	0.49	0.49
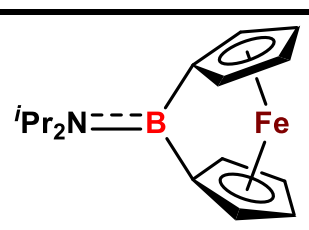	0.92	-0.91	0.51	0.51	0.51	0.47	0.47
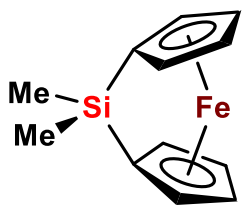	0.93	-0.92	0.52	0.51	0.51	0.47	0.47
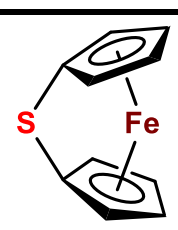	0.93	-0.92	0.52	0.50	0.50	0.46	0.46
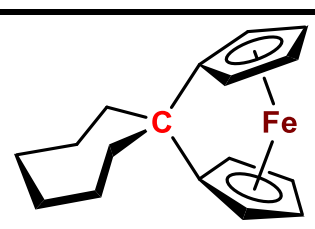	0.90	-0.89	0.51	0.51	0.51	0.46	0.46
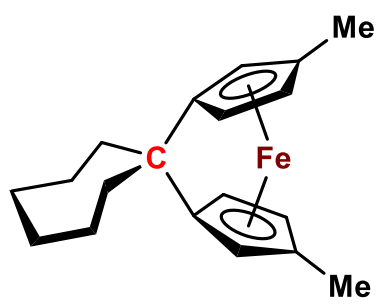	0.89	-0.89	0.51	0.51	0.51	0.46	0.44
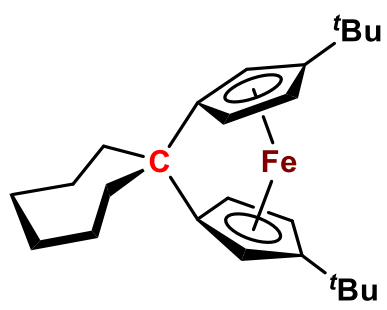	0.88	-0.87	0.51	0.51	0.50	0.46	0.44

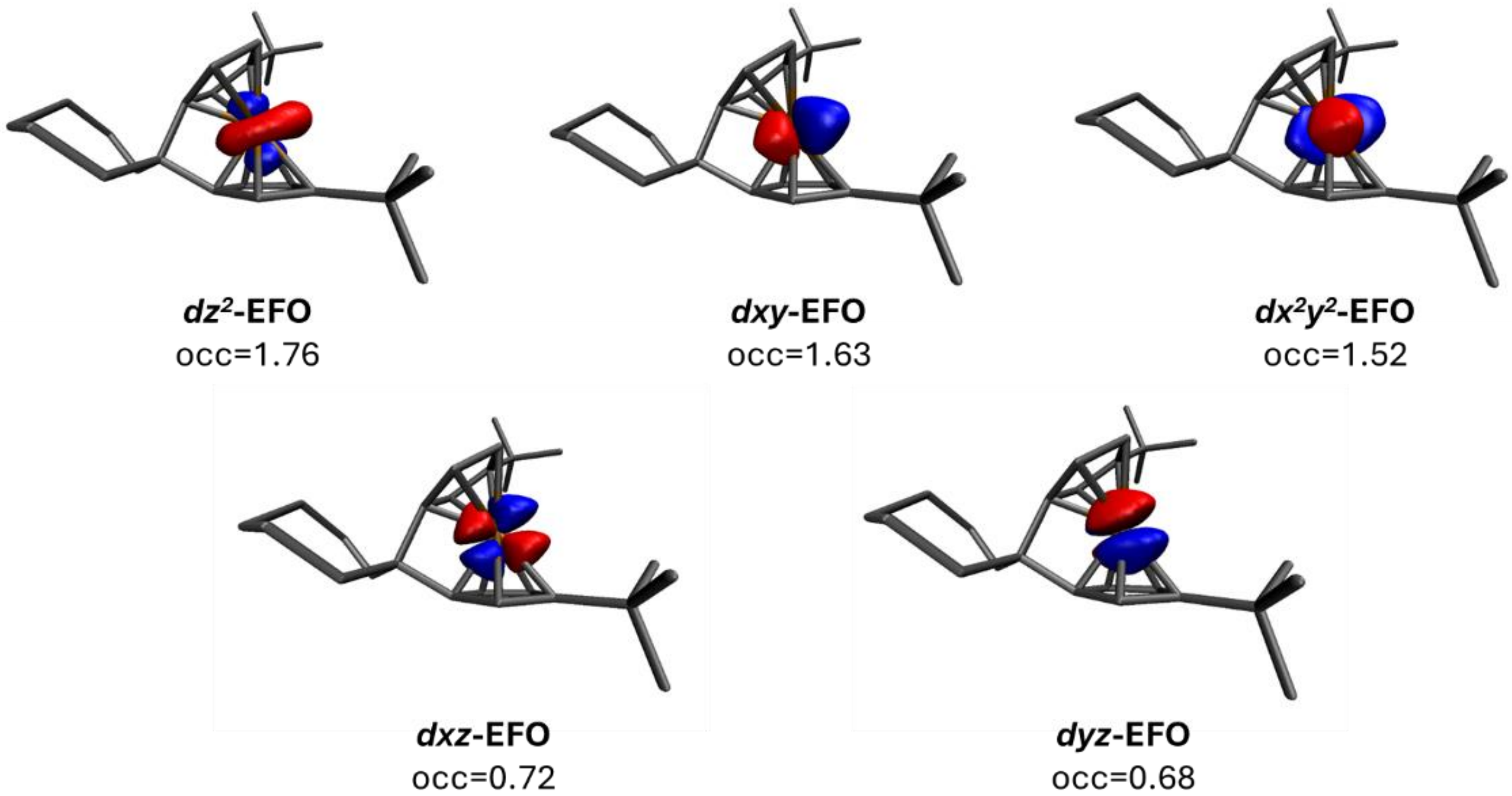

Figure S32: Selected EFOs of iron in **3**, calculated at the B3PW91-D3(BJ)/def2-TZVP//B3PW91-D3(BJ)/def2-SVP level of theory.

Table S3: EOS and Fe centered *d*-type EFO occupations of different [1]ferrocenophanes, calculated at the B3PW91-D3(BJ)/def2-TZVP//B3PW91-D3(BJ)/def2-SVP level of theory.

	**EFO** d_{z^2}	**EFO** d_{xy}	**EFO** $d_{x^2-y^2}$	**EFO** d_{xz}	**EFO** d_{yz}	**EOS** **Fe**	**R** **(%)**
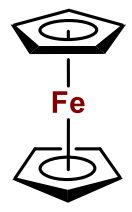	1.757	1.584	1.582	0.661	0.608	2	95
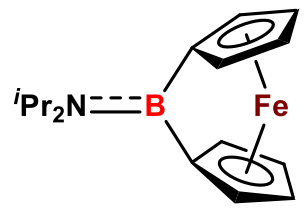	1.771	1.623	1.513	0.701	0.670	2	80
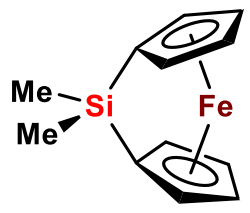	1.766	1.613	1.520	0.691	0.674	2	81
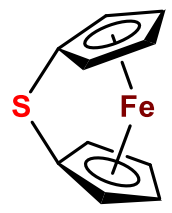	1.770	1.631	1.514	0.689	0.677	2	81
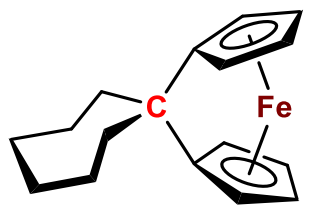	1.776	1.632	1.514	0.712	0.670	2	79
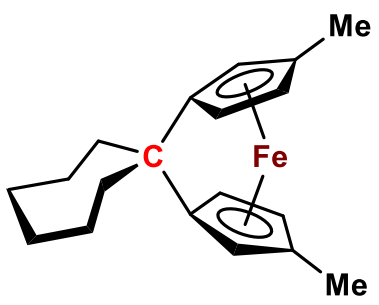	1.771	1.632	1.507	0.718	0.676	2	78
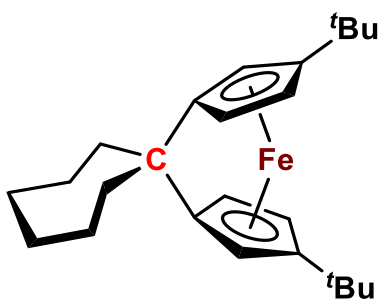	1.768	1.625	1.512	0.723	0.678	2	78

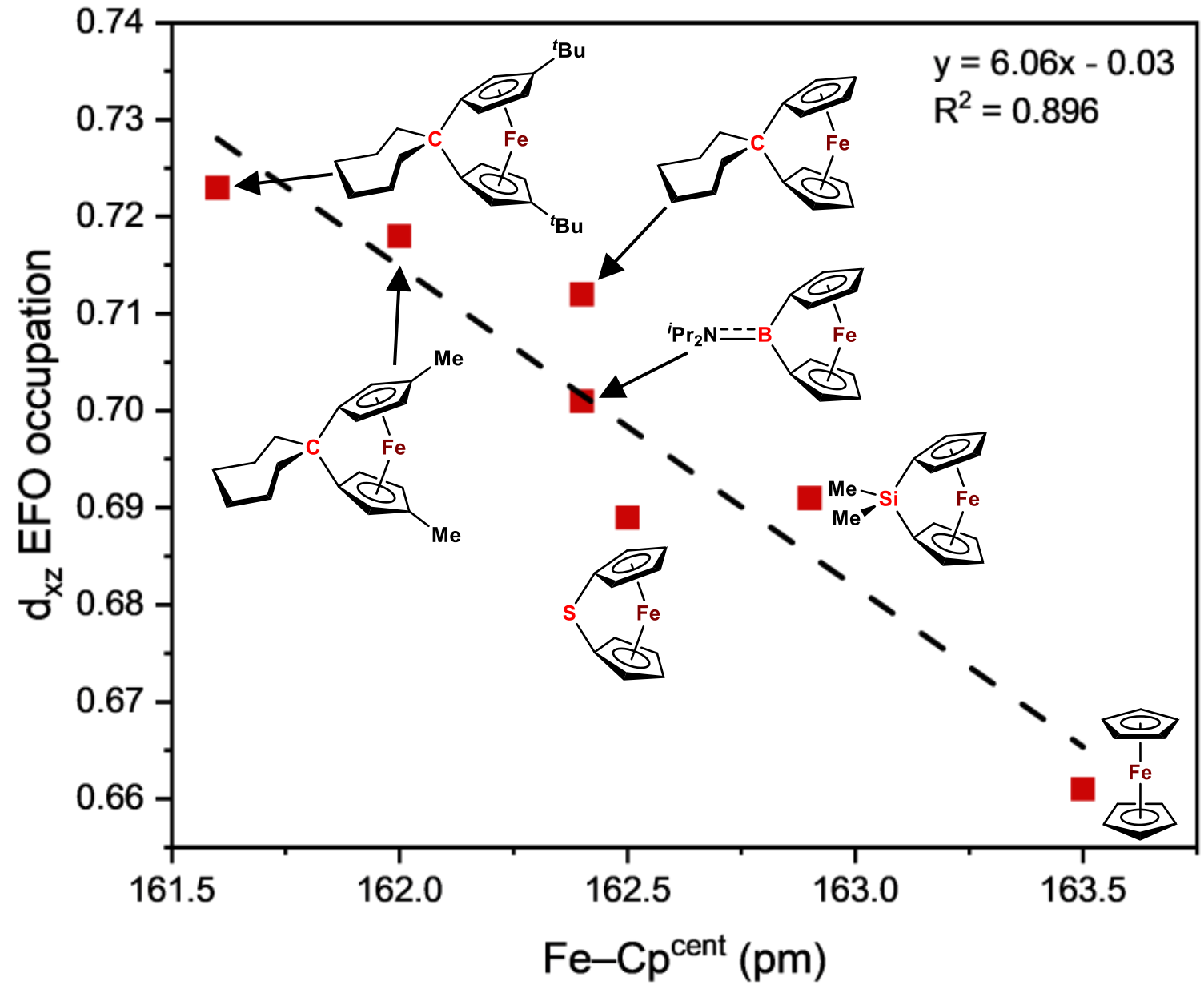

Figure S33: Correlation between the Fe–Cp^{cent} distances and d_{xz} EFO occupations, calculated at the B3PW91-D3(BJ)/def2-TZVP//B3PW91-D3(BJ)/def2-SVP level of theory.

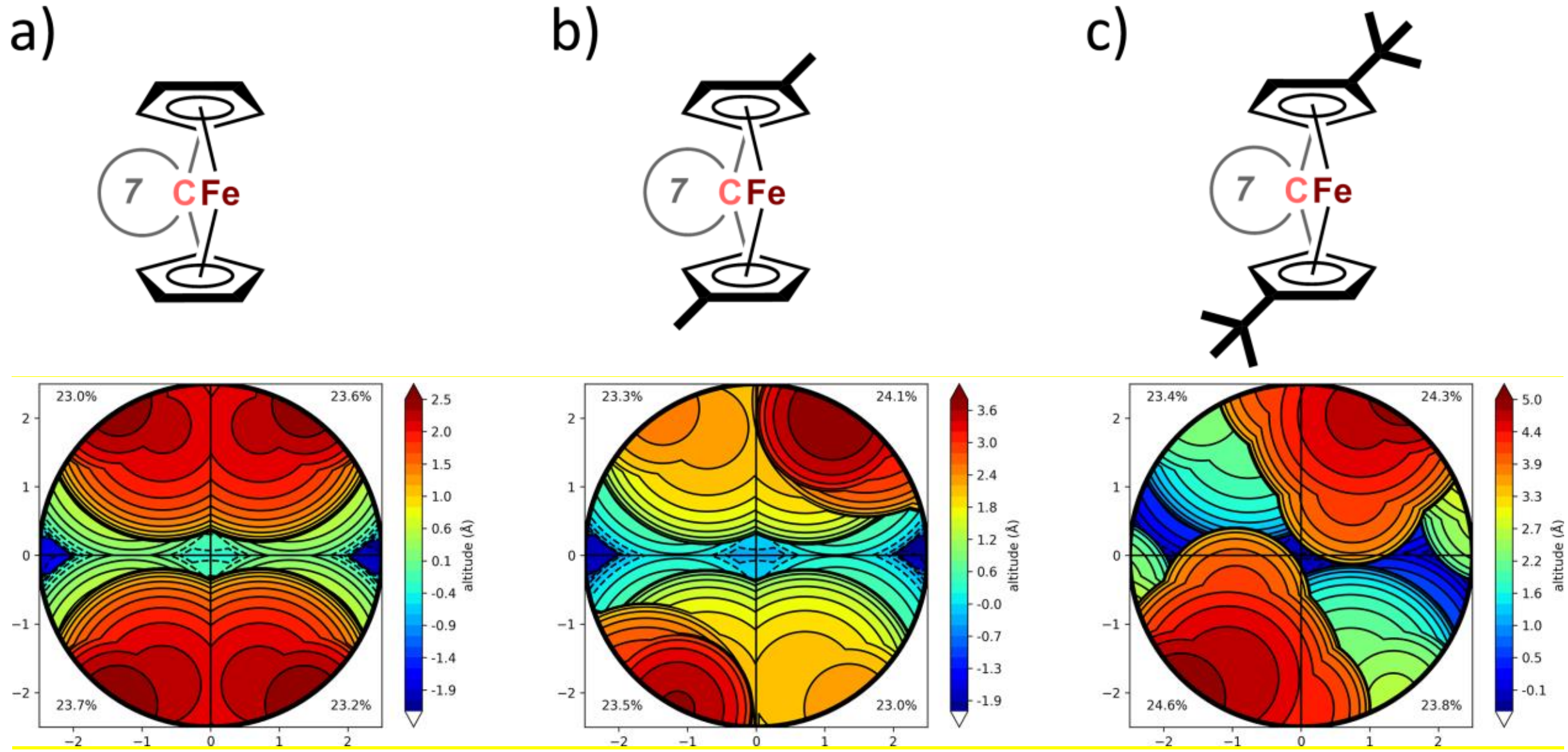

Figure S34: 2D buried volume maps[115] of C[1]ferrocenophane **3** (c)) and its Cp^{H}- (a)) and Cp^{Me}-substituted (b)) theoretical derivatives.

Table S4: EDA results for different [1]ferrocenophanes for the fragmentation LFe → L^{2-} + Fe^{2+}, calculated at the B3LYP-D3(BJ)/TZ2P//B3PW91-D3(BJ)/def2-SVP level of theory (energies in kJ mol^{-1}).

	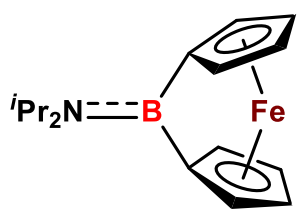	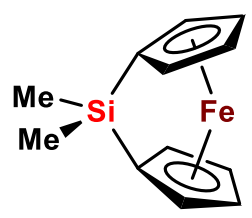	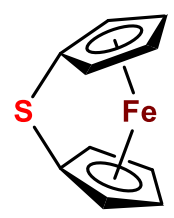	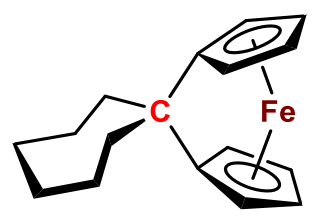	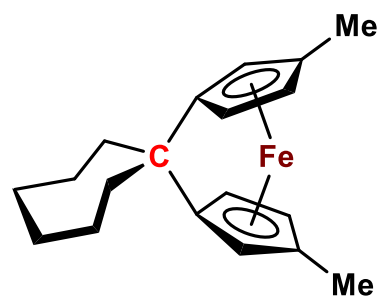	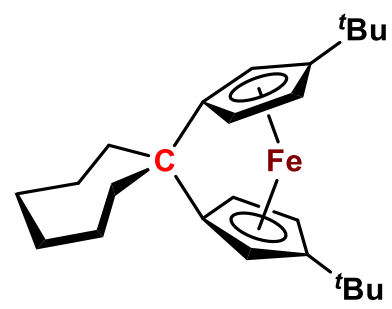
ΔE_{int}	-3447.2	-3439.7	-3380.3	-3406.2	-3410.8	-3377.3
ΔE_{Pauli}	1384.5	1344.7	1383.2	1408.3	1429.3	1443.9
ΔE_{elstat}[a]	-2519.6 (52.1%)	-2509.6 (52.5%)	-2525.0 (53.0%)	-2520.9 (52.4%)	-2498.7 (51.6%)	-2416.3 (50.1%)
ΔE_{disp}[a]	-10.9 (0.2%)	-12.1 (0.3%)	-11.7 (0.2%)	-12.6 (0.3%)	-14.6 (0.3%)	-19.2 (0.4%)
ΔE_{orb}	-2302.9	-2264.0	-2227.6	-2282.4	-2328.4	-2388.2
$\Delta E_{orb\text{-}HF}$	1.3	1.3	0.8	1.7	1.7	2.5
$\Delta E_{orb\text{-}corr}$[a]	-2301.6 (47.6%)	-2262.7 (47.3%)	-2226.7 (46.7%)	-2281.1 (47.4%)	-2326.7 (48.1%)	-2385.7 (49.5%)
ΔE_{prepL}	201.7	182.8	152.7	156.5	149.4	140.6
ΔE_{prepFe}[b]	326.0	326.0	326.0	326.0	326.0	326.0
D_e	2919.6	2930.9	2901.2	2923.8	2935.5	2910.4

[a] Values in parentheses give the percentage contributions to the total attractive interaction (ΔE_{elstat} + ΔE_{disp} + $\Delta E_{orb\text{-}corr}$).
[b] Preparation energies of iron are obtained considering high-spin Fe^{2+} (^{5}D).

We have analyzed a series of [1]ferrocenophanes featuring different elements as *ansa*-bridges (carbon, silicon, sulfur and boron). All the complexes display similar dissociation energies D_e, ranging from 2901 to 2936 kJ mol^{-1}. These values result from a balance between the interaction energy ΔE_{int} and the preparation energy ΔE_{prep}. In general, the complexes with stronger interaction energy exhibit higher preparation energy and *vice versa*, yielding similar dissociation energies D_e. The attractive interactions show comparable relative contribution (ca. 52% electrostatics (ΔE_{elstat}), ca. 48% orbital interaction (ΔE_{orb}), <1% dispersion interaction (ΔE_{disp})), suggesting a consistent bonding situation across the series. The complexes with short Fe–Cp^{cent} distances display stronger orbital stabilizations, in agreement with the Mayer's effective orbitals (EFOs) analysis (*vide supra*). However, shorter Fe–Cp^{cent} distances, correlate with higher Pauli repulsion ΔE_{Pauli} (Figure S35). Nonetheless, the orbital (ΔE_{orb}) and electrostatic (ΔE_{elstat}) stabilization largely surpass the Pauli repulsion.

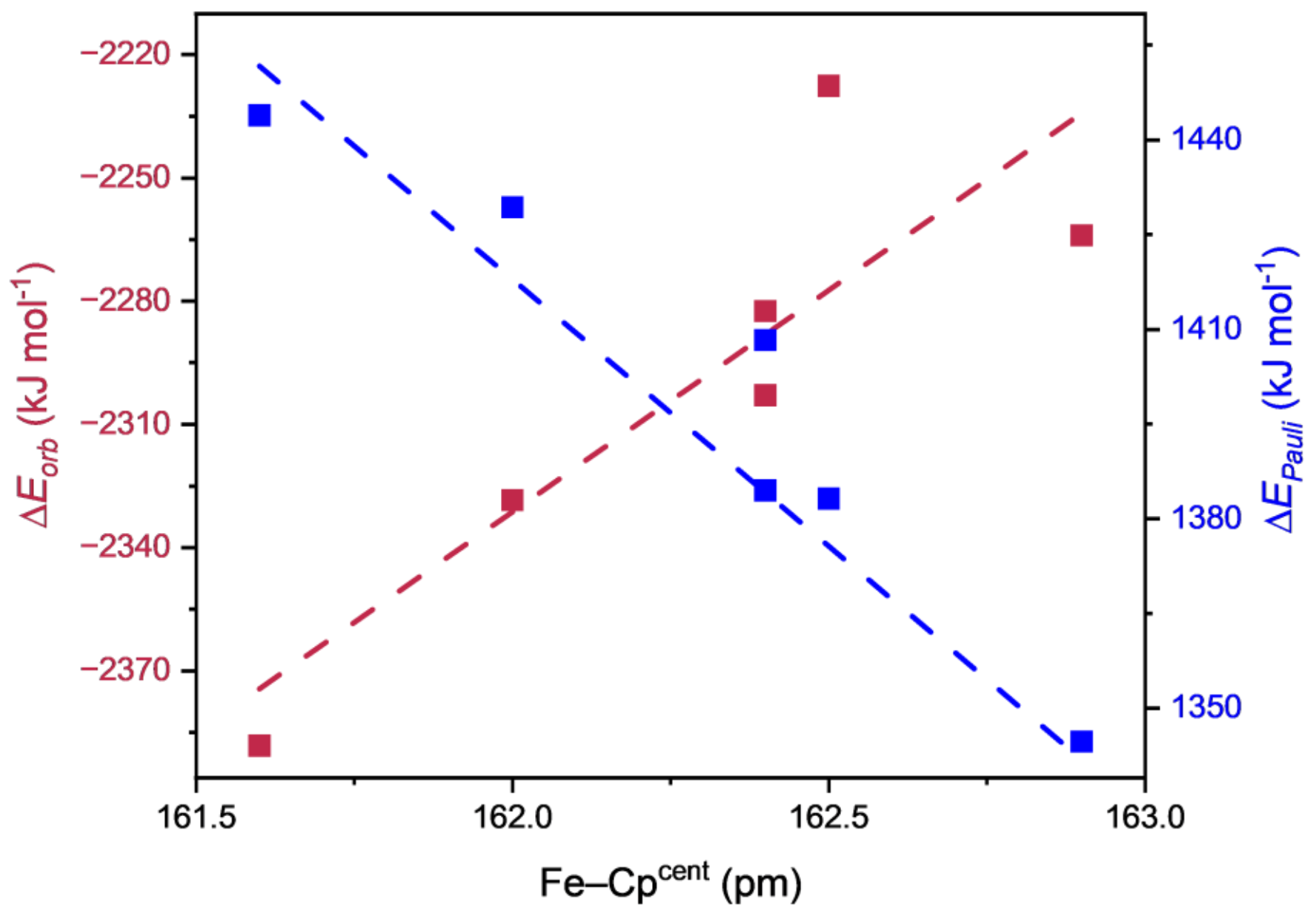

Figure S35: Correlation between the Fe–Cp^{cent} distances and the ΔE_{orb} and ΔE_{Pauli} contributions, obtained from the EDA for the fragmentation LFe → L^{2-} + Fe^{2+}, calculated at the B3PW91-D3(BJ)/def2-TZVP//B3PW91-D3(BJ)/def2-SVP level of theory.

Furthermore, it is worth noting that in **3**, the electrostatic stabilization ΔE_{elstat} is the weakest (-2416 kJ mol^{-1}) compared to the rest of the analyzed [1]ferrocenophanes (ca. -2510 kJ mol^{-1}). This can be rationalized by the closer proximity of the Cp scaffold to the iron center, resulting in an overall more repulsive interaction.

Noteworthy, EDA predicts the dispersion interactions to be the smallest contribution term in all the analyzed [1]ferrocenophanes. However, the NCI plots show a relevant van der Waals stabilization between the *tert*-butyl substituents in **3**. This attractive interaction cannot be captured by EDA because of the considered LFe → L^{2-} + Fe^{2+} fragmentation and hence both Cp rings being part of the same ligand fragment. In contrast, the NCI plot is a fragment-independent method, allowing the identification of these interactions. To understand the role of the substituents at the Cp rings with regards to dispersion interactions and their stabilization of the complexes, we performed EDA of three ferrocene model compounds, Cp_2Fe, $Cp^{Me}{}_2Fe$ and $Cp^{tBu}{}_2Fe$, with the same bent geometry as their ferrocenophane derivatives. With these models, it is possible to perform EDA based on three fragments, allowing to approximate the dispersion interactions of the Cp scaffolds (Table S5). Indeed, the dispersion interaction ΔE_{disp} increases from -44.4 kJ mol^{-1} (Cp_2Fe) to -64.9 kJ mol^{-1} ($Cp^{tBu}{}_2Fe$). To further probe the role of the dispersion stabilization, we optimized the complexes $(C_6H_{12})C[1]Cp^{Me}{}_2Fe$ and $(C_6H_{12})C[1]Cp^{tBu}{}_2Fe$, **3**, without applying Grimme's dispersion correction (Table S6). In the case of the Cp^{Me}-substituted derivative, the Fe–Cp^{cent} distance is computed to be slightly shorter, when dispersion interactions are considered (with GD3-BJ: 162.0 pm; without GD3-BJ: 162.9 pm), whereas for **3**, this effect is substantially more pronounced (with GD3-BJ: 161.6 pm; without GD3-BJ: 163.3 pm). Furthermore, without dispersion, the interaction energy decreases, mainly due to a reduction of the orbital interaction ΔE_{orb}. These results highlight the interplay between dispersion ΔE_{disp} and orbital interactions ΔE_{orb} in determining the overall bonding energy and geometry.

Table S5: EDA results for different bend ferrocenes with the fragmentation $L_2Fe \rightarrow 2\ L^{-} + Fe^{2+}$, calculated at the B3LYP-D3(BJ)/TZ2P//B3PW91-D3(BJ)/def2-SVP level of theory (energies in kJ mol^{-1}).

	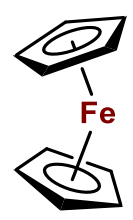	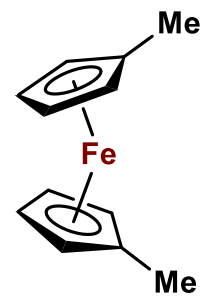	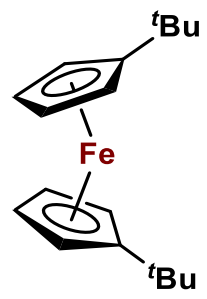
ΔE_{int}	-3025.5	-3046.0	-3039.7
ΔE_{Pauli}	2040.1	2061.0	2076.5
ΔE_{elstat}	-2992.4	-2990.7	-2934.7
ΔE_{disp}	-44.4	-49.4	-64.9
ΔE_{orb}	-2028.8	-2067.3	-2117.9
$\Delta E_{orb\text{-}HF}$	0.4	0.8	1.3
$\Delta E_{orb\text{-}corr}$	-2028.4	-2066.9	-2116.7

Table S6: EDA results for **3** and its Cp^{Me}-substituted theoretical derivative for the fragmentation LFe → L^{2-} + Fe^{2+}, calculated at the B3LYP/TZ2P//B3PW91/def2-SVP and B3LYP-D3(BJ)/TZ2P//B3PW91-D3(BJ)/def2-SVP levels of theory (distances in pm and energies in kJ mol^{-1}).

	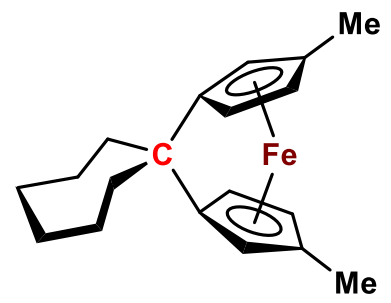		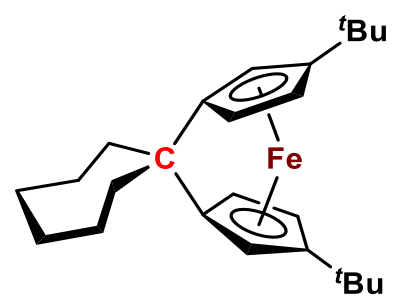	
	**(*with* GD3-BJ)**	**(*without* GD3-BJ)**	**(*with* GD3-BJ)**	**(*without* GD3-BJ)**
ΔE_{int}	-3410.8	-3397.0	-3377.3	-3359.8
ΔE_{Pauli}	1429.3	1388.3	1443.9	1373.6
ΔE_{elstat}	-2498.7	-2486.6	-2416.3	-2397.9
ΔE_{disp}	-14.6	-	-19.2	-
ΔE_{orb}	-2328.4	-2300.4	-2388.2	-2338.0
$\Delta E_{orb\text{-}HF}$	1.7	1.7	2.5	2.5
$\Delta E_{orb\text{-}corr}$	-2326.7	-2298.7	-2385.7	-2335.5
**Fe–Cp^{cent}**	162.0	162.9	161.6	163.3

Table S7: Decomposition of the NCI into pairwise Pipek-Mezey orbitals of the Cp^{H}-substituted theoretical derivative of **3**, calculated at the B3PW91-D3(BJ)/def2-TZVP//B3PW91-D3(BJ)/def2-SVP level of theory (S_AB = bicentric orbital pair contribution; isocontour = 0.15 a. u.).

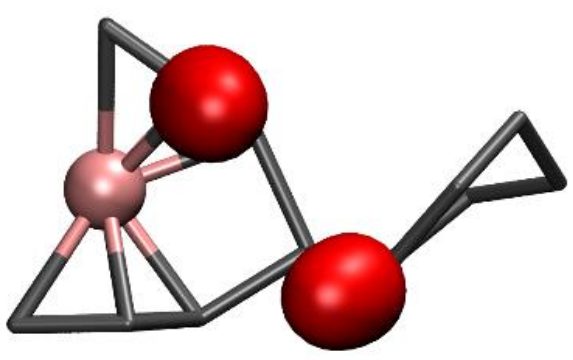	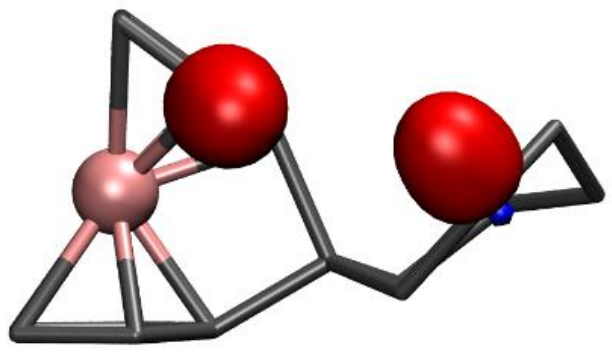
**S_AB=14.77**	**S_AB=22.04**
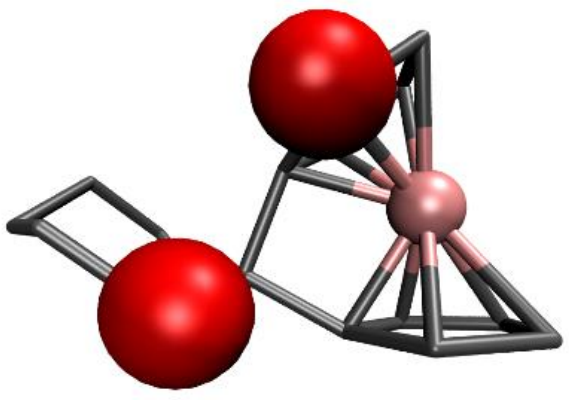	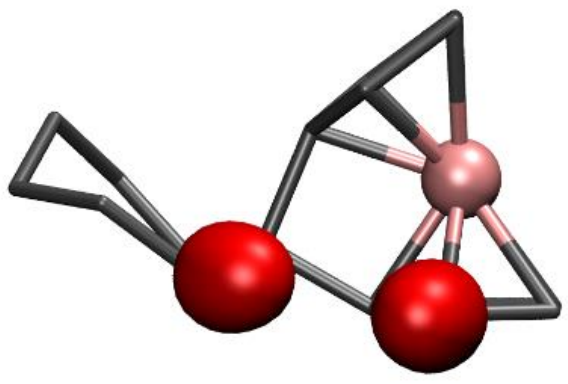
**S_AB=20.09**	**S_AB=18.91**
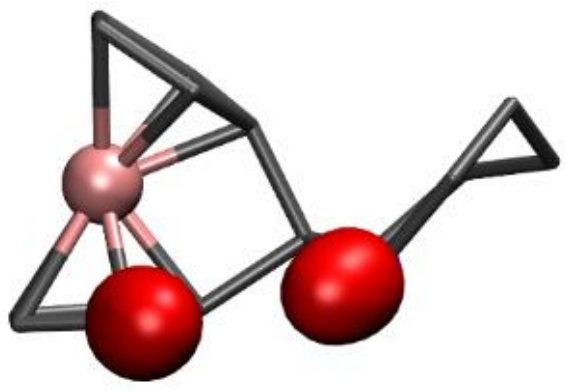	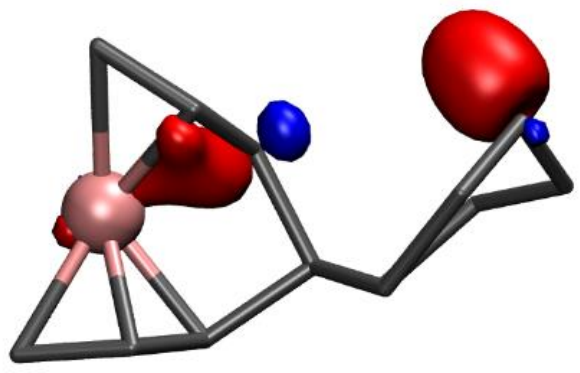
**S_AB=17.48**	**S_AB=17.82**
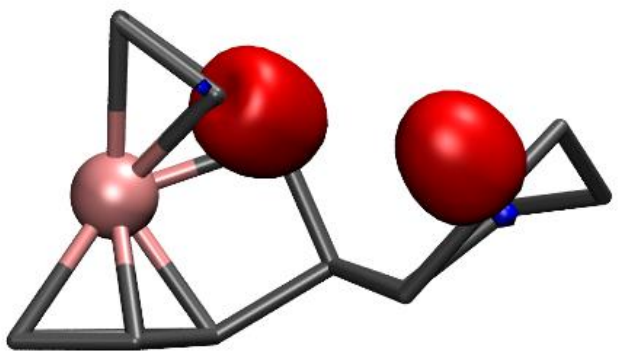	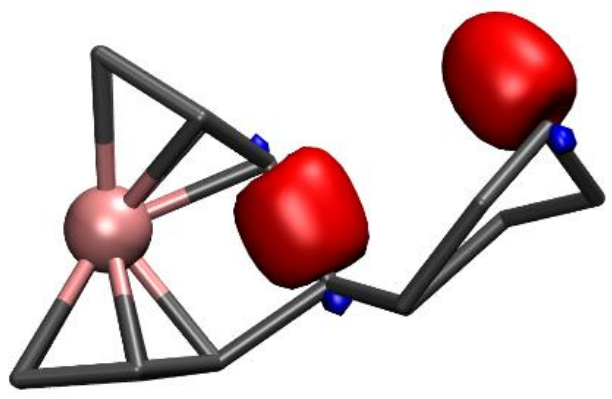
**S_AB=10.02**	**S_AB=11.94**
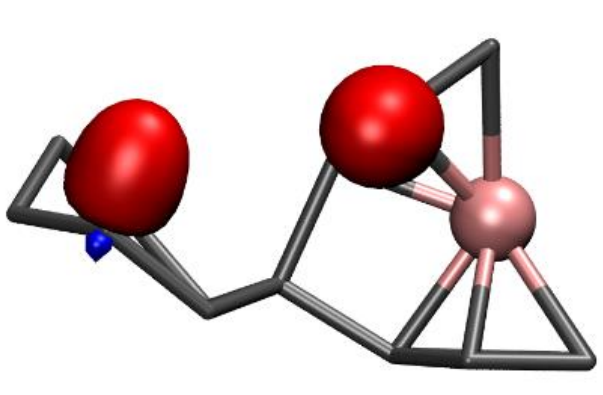	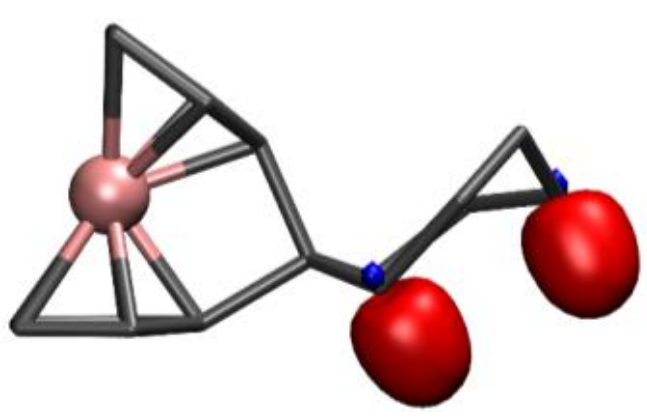
**S_AB=9.40**	**S_AB=7.80**
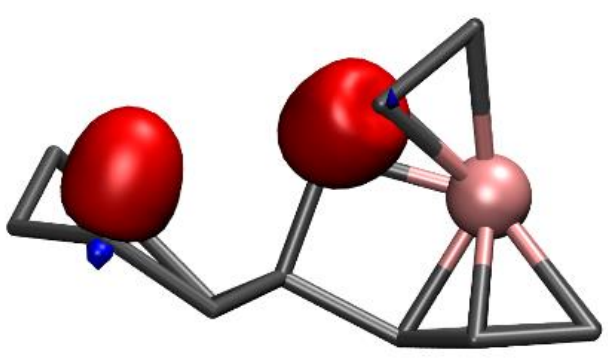	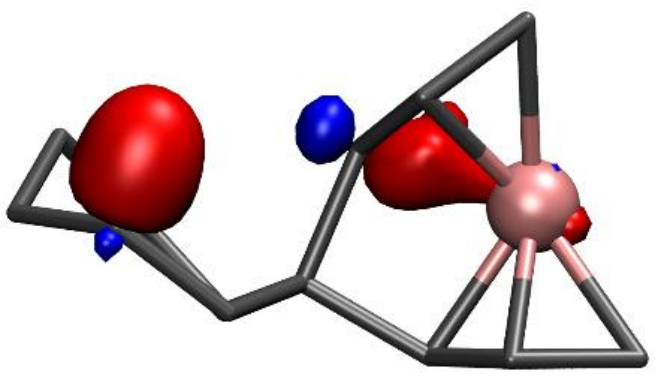

Table S8: Decomposition of the NCI into pairwise Pipek-Mezey orbitals of **3** at the B3PW91-D3(BJ)/def2-TZVP//B3PW91-D3(BJ)/def2-SVP level of theory (S_AB = bicentric orbital pair contribution; isocontour = 0.15 a. u.)

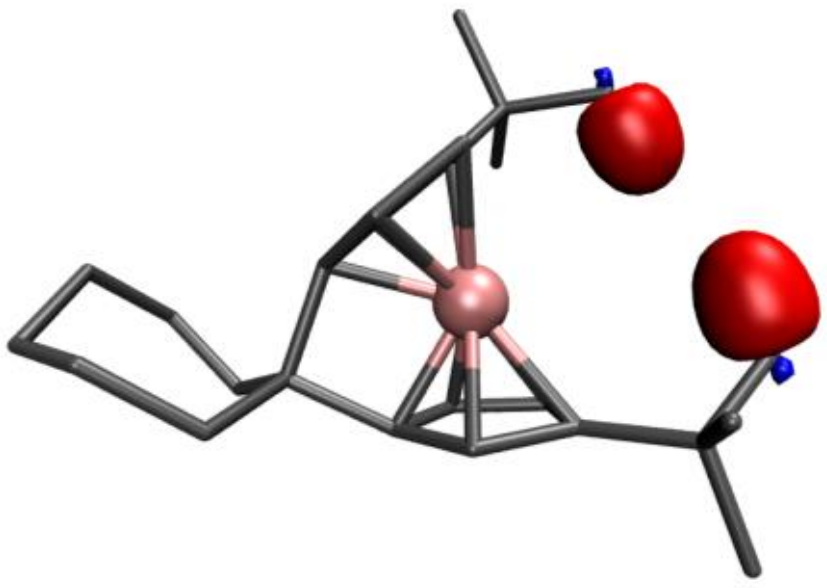

S_AB=18.64

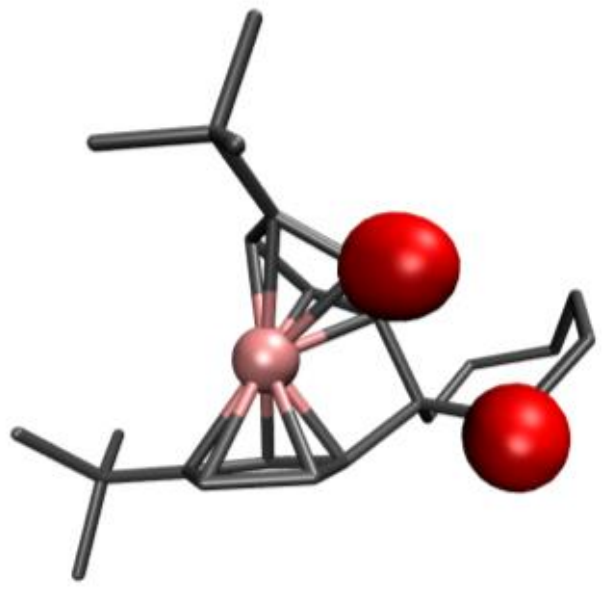

S_AB=15.83

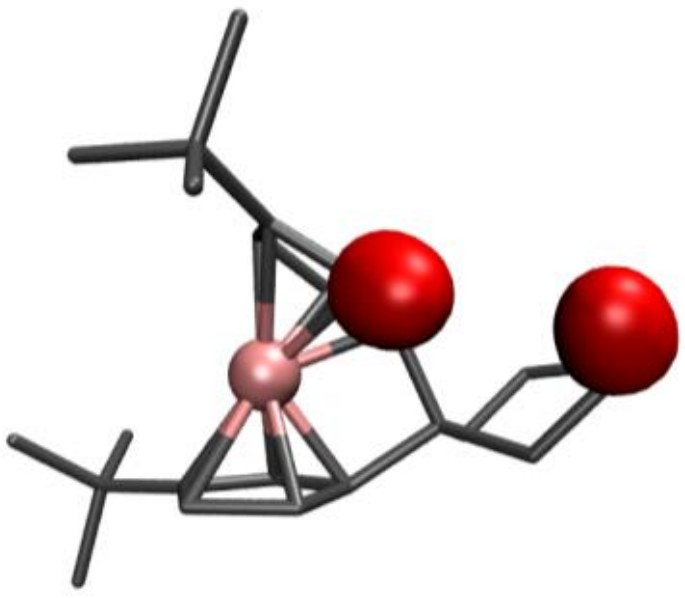

S_AB=23.04

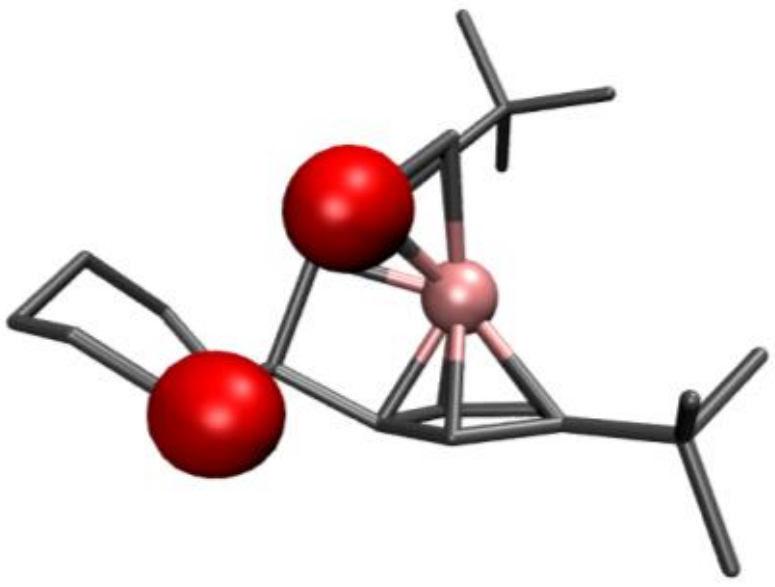

S_AB=19.05

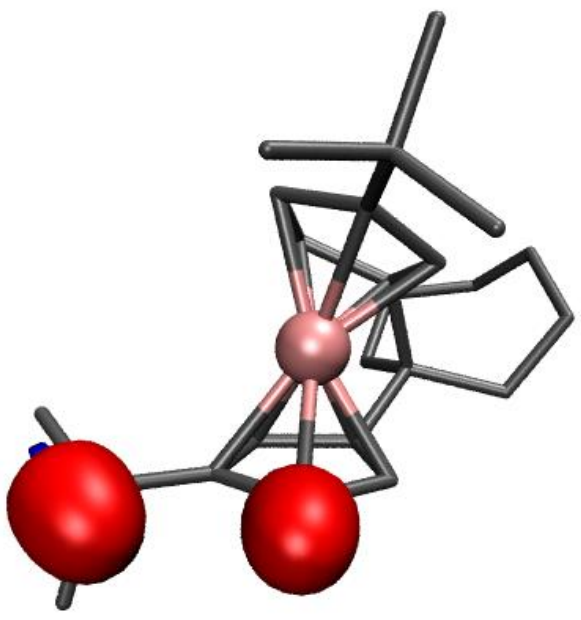

S_AB=14.64

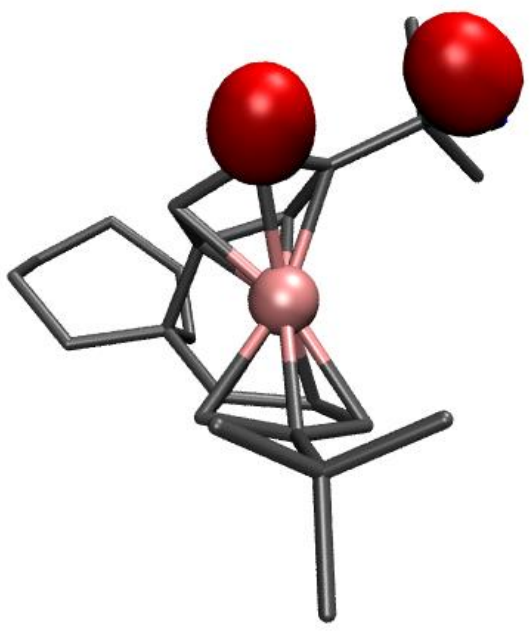

S_AB=14.41

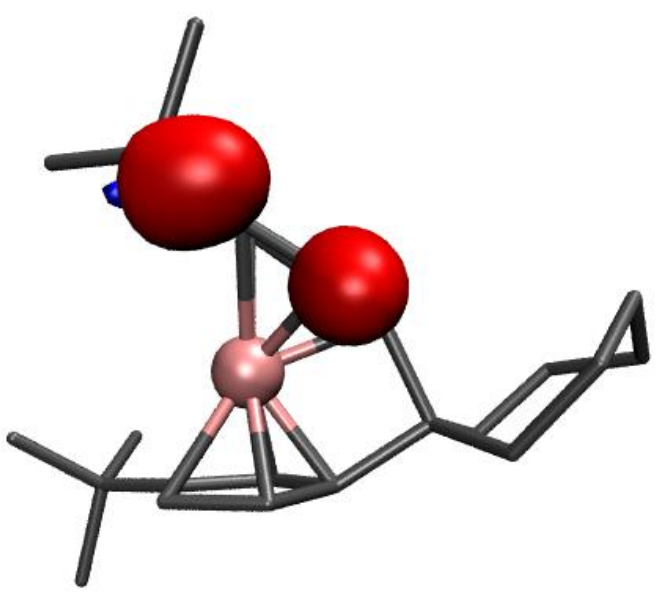

S_AB=15.83

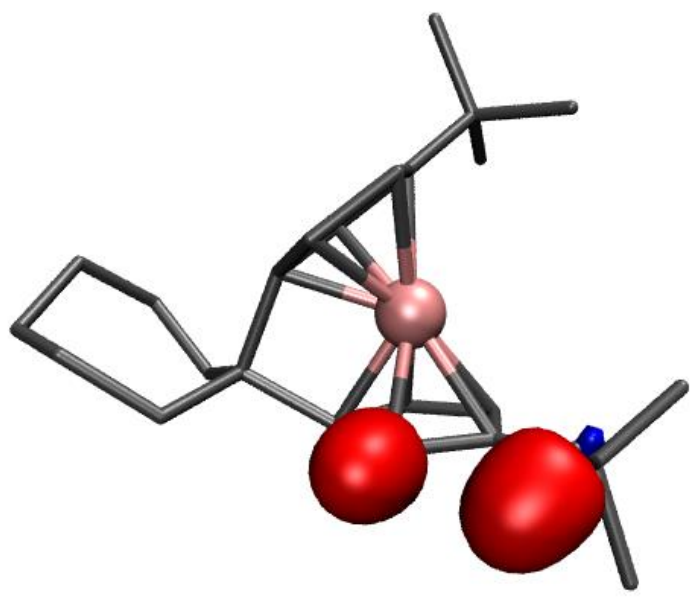

S_AB=15.71

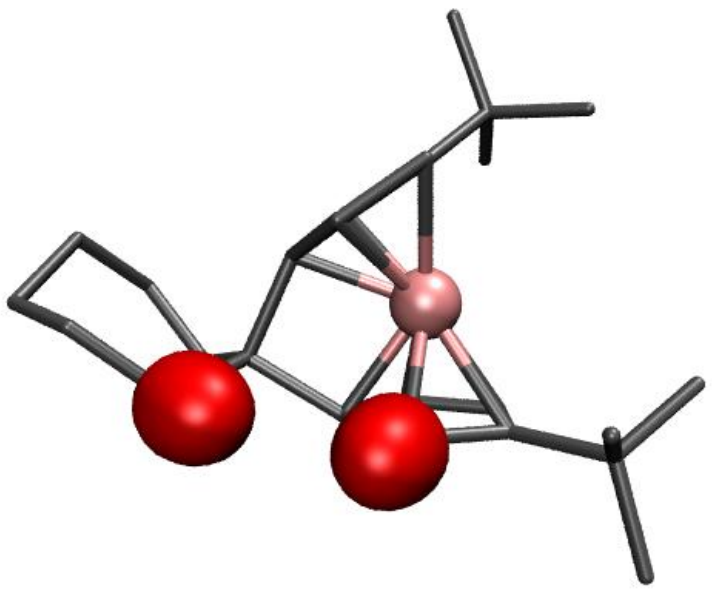

S_AB=19.47

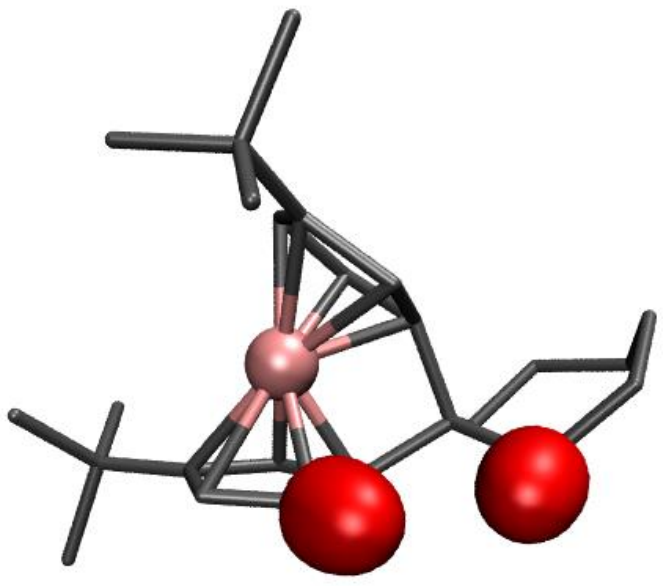

S_AB=16.72

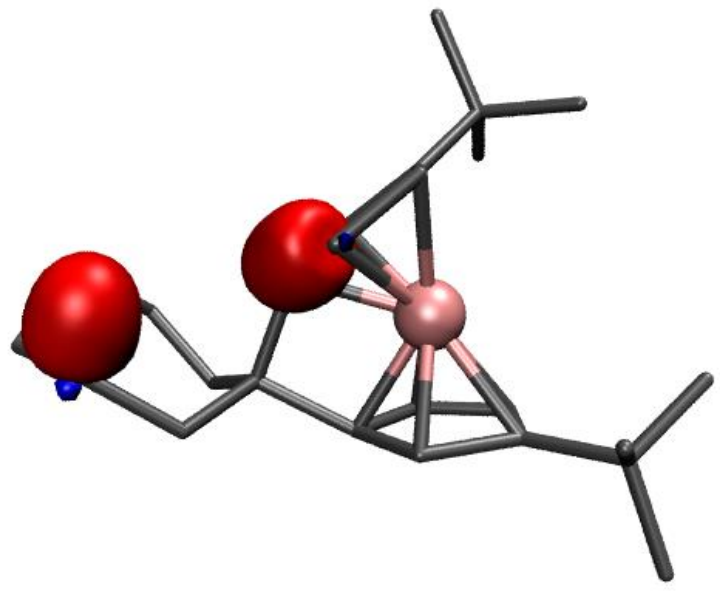

S_AB=12.40

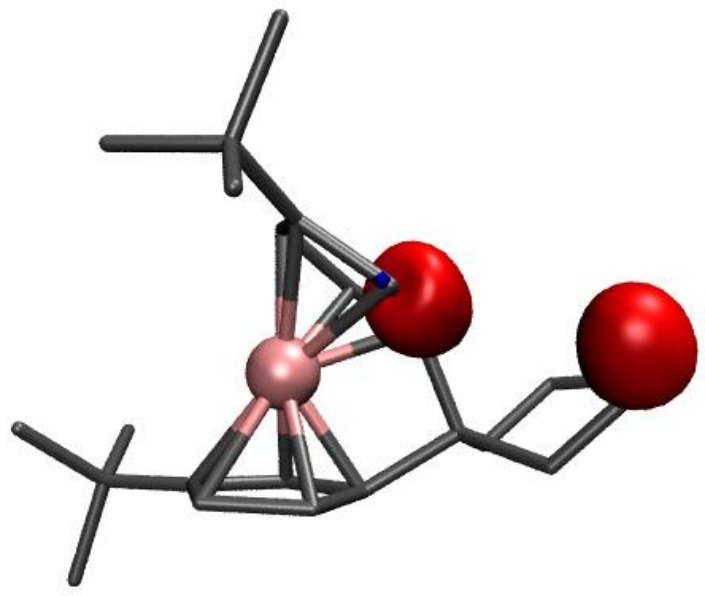

S_AB=11.32

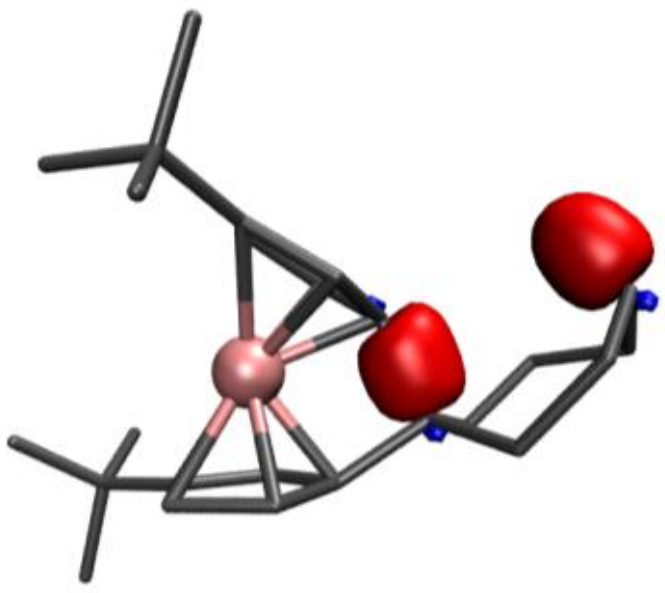

S_AB=15.25

11. Overview of selected [1]ferrocenophanes

A large variety of [1]ferrocenophanes has been reported, including all conceivable heavy 3rd and 4th row *p*-block elements: Aluminum, Silicon, Phosphorous, Sulfur, Gallium, Germanium, Arsenic and Selenium, as well as the 5th row *p*-block elements Indium, Tin and Antimony. Table S9 summarizes selected parameters and references for reported crystal structures of the before mentioned [1]ferrocenophanes, as well as of the 2nd row *p*-block elements Boron and the herein reported Carbon. It should be mentioned that beside these, *d*-block and *f*-block metal-bridged [1]ferrocenophanes are also known, but not listed in Table S9.[116-123]

Table S9: Selected structural parameters for different [1]ferrocenophanes obtained from single crystal structures and their references, arranged by atomic number of the bridging *p*-block element (due to the large number of Si[1]- and P[1]ferrocenophanes, only the first example of each is listed, as well as the famous Me_2Si[1]ferrocenophane).

E		[Ref]	α [°]	β/β' [°]	θ [°]	δ [°]
B	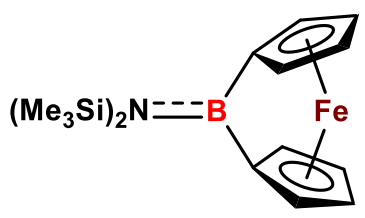	38, 44	32.4	33.8/ 33.4	100.1	155.2
	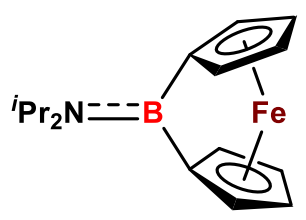	44	31.4	35.3/ 34.4	102.0	155.9
			31.0	35.6/ 35.2	103.3	156.1
	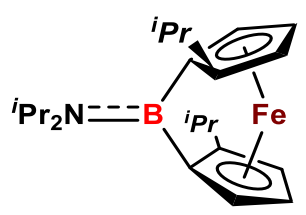	124	31.9	34.6/ 36.1	103.1	155.9
	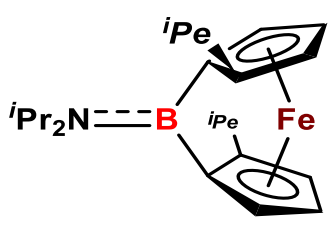	125	31.2	36.1/ 35.3	103.4	156.3
	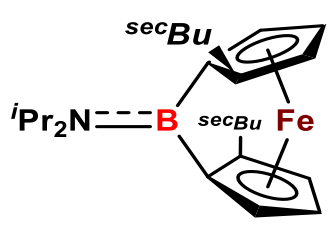	126	31.4	35.2/ 35.7	103.0	156.1
C	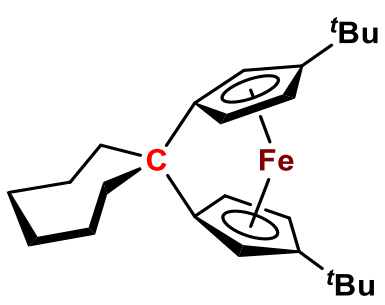		36.7	29.4/ 29.7	97.0	151.8
Al	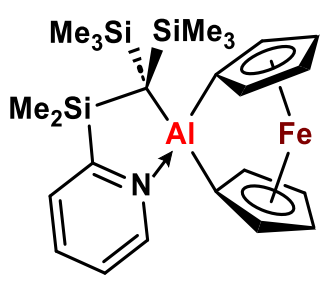	47	14.8	39.6/ 40.5	94.8	168.2
	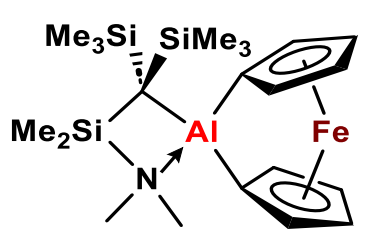	127	14.3	39.8/ 39.4	94.5	168.2

Si	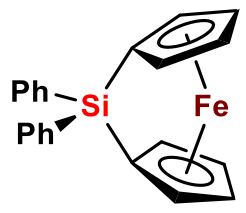	91	19.1	40.0	99.2	167.3
	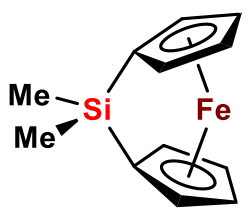	72<br>(128)	20.4<br>(20.6)	37.5/<br>37.8<br>(36.6/<br>37.4)	96.1<br>(95.7)	164.9<br>(164.7)
P	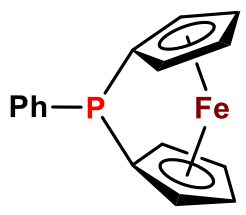	64<br>(48)	26.7,<br>(26.9)	32.4/<br>31.6,<br>(32.1/<br>32.3)	90.6<br>(90.7)	159.9<br>(159.8)
S	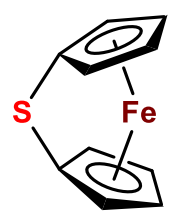	45,<br>40	31.1	29.1/<br>28.7	89.0	156.9
	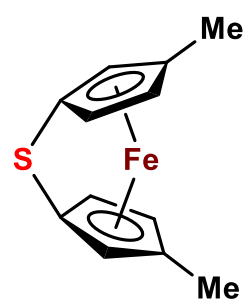	40	31.5	28.9	89.3	156.9
Ga	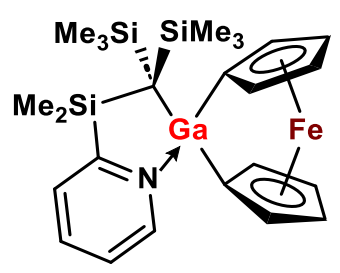	65,<br>129	15.5<br>16.4	38.3/<br>39.0<br>37.6/<br>38.50	92.7<br>92.2	167.0<br>166.3
	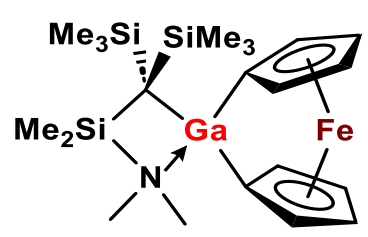	127	15.8	38.4/<br>38.3	92.8	166.8
	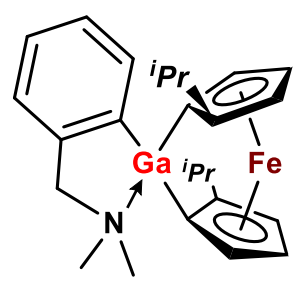	124	16.5<br>16.3	38.4/<br>38.9<br>39.1/<br>40.0	93.0<br>93.4	166.8<br>166.7

	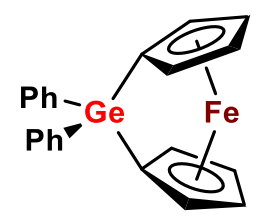	64	16.6	37.7	93.6	166.2
	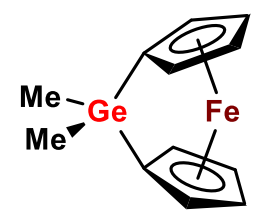	130	18.8	35.7/ 36.9	91.7	165.2
**Ge**	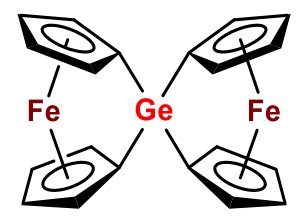	131	19.2	37.5/ 38.4	94.7	165.2
	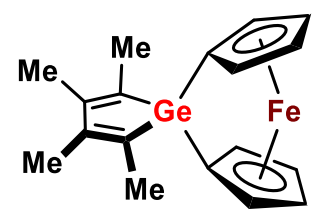	132	19.2	36.7	92.3	165.2
	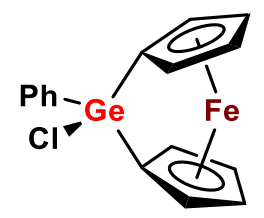	133	18.4	38.7/ 38.1	95.2	166.3
**As**	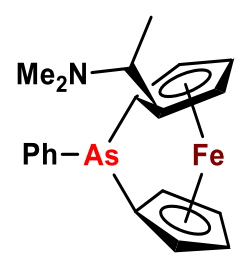	48	22.9	32.8/ 33.3	87.9	162.5
**Se**	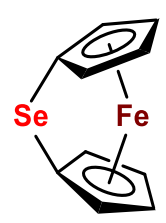	40	26.4	30.0	86.0	160.0
	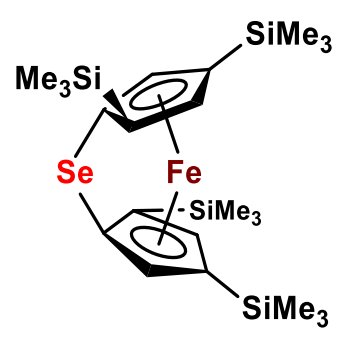	134	27.0	29.7	97.0	160.3

	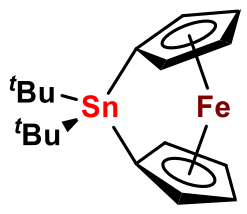	68, 135	14.1	36.2	86.5	168.6
	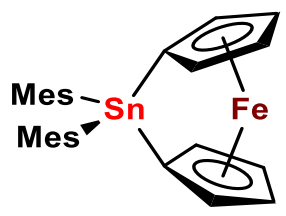	135	15.4	35.2/ 35.3	85.7	167.5
			15.7	35.4/ 35.9	85.9	167.3
**Sn**			14.5	36.5/ 35.4	85.9	167.9
	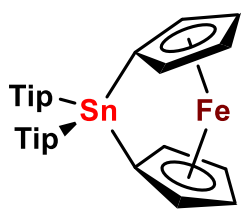	136	14.7	36.0/ 37.5	85.9	168.0
	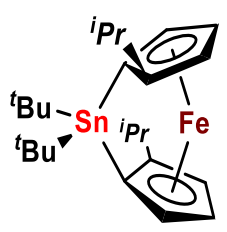	124	14.6	36.7/ 36.2	86.4	168.5
			13.5	37.3/ 36.9		169.0
**Sb**	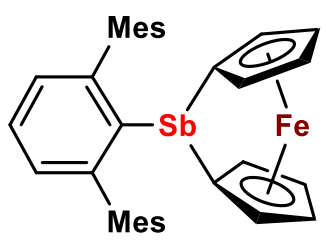	69	18.7	34.7/ 32.9	83.0	165.3

12. References

[93] G. R. Fulmer, A. J. M. Miller, N. H. Sherden, H. E. Gottlieb, A. Nudelman, B. M. Stoltz, J. E. Bercaw, and K. I. Goldberg, "NMR Chemical Shifts of Trace Impurities: Common Laboratory Solvents, Organics, and Gases in Deuterated Solvents Relevant to the Organometallic Chemist," *Organometallics* 29 (2010): 2176–2179, https://doi.org/10.1021/om100106e.

[94] J. Paradies, G. Kehr, R. Fröhlich, and G. Erker, "*Ansa*-Metallocene Polymerization Catalysts Derived from [2+2]Cycloaddition Reactions of Bis(1-methylethenyl-cyclopentadienyl)zirconium Systems," *PNAS* 103 (2006): 15333–15337, https://doi.org/10.1073/pnas.0602627103.

[95] G. M. Sheldrick, "SHELXT - Integrated Space-Group and Crystal-Structure Determination," *Acta Crystallographica A* 71 (2015): 3–8, https://doi.org/10.1107/S2053273314026370.

[96] G. M. Sheldrick, "Crystal structure refinement with SHELXL," *Acta Crystallographica C* 71 (2015): 3–8, https://doi.org/10.1107/S2053229614024218.

[97] C. B. Hübschle, G. M. Sheldrick, and B. Dittrich, "ShelXle: a Qt Graphical User Interface for SHELXL," *Journal of Applied Crystallography* 44 (2011): 1281–1284, https://doi.org/10.1107/S0021889811043202.

[98] M. J. Frisch, G. W. Trucks, H. B. Schlegel, G. E. Scuseria, M. A. Robb, J. R. Cheeseman, G. Scalmani, V. Barone, G. A. Petersson, H. Nakatsuji, X. Li, M. Caricato, A. V. Marenich, J. Bloino, B. G. Janesko, R. Gomperts, B. Mennucci, H. P. Hratchian, J. V. Ortiz, A. F. Izmaylov, J. L. Sonnenberg, D. Williams-Young, F. Ding, F. Lipparini, F. Egidi, J. Goings, B. Peng, A. Petrone, T. Henderson, D. Ranasinghe, V. G. Zakrzewski, J. Gao, N. Rega, G. Zheng, W. Liang, M. Hada, M. Ehara, K. Toyota, R. Fukuda, J. Hasegawa, M. Ishida, T. Nakajima, Y. Honda, O. Kitao, H. Nakai, T. Vreven, K. Throssell, J. A. Montgomery, Jr., J. E. Peralta, F. Ogliaro, M. J. Bearpark, J. J. Heyd, E. N. Brothers, K. N. Kudin, V. N. Staroverov, T. A. Keith, R. Kobayashi, J. Normand, K. Raghavachari, A. P. Rendell, J. C. Burant, S. S. Iyengar, J. Tomasi, M. Cossi, J. M. Millam, M. Klene, C. Adamo, R. Cammi, J. W. Ochterski, R. L. Martin, K. Morokuma, O. Farkas, J. B. Foresman, and D. J. Fox, Gaussian 16, Revision C.01; Gaussian, Inc.: Wallingford CT, (2019).

[99] A. D. Becke, "Density-Functional Thermochemistry. III. The Role of Exact Exchange," *The Journal of Chemical Physics* 98 (1993): 5648–5652, https://doi.org/10.1063/1.464913.

[100] J. P. Perdew in *Electronic Structure of Solids '91*, (Eds.: P. Ziesche, H. Eschrig), Akademie Verlag: Berlin, (1991).

[101] A. D. McLean and G. S. Chandler, "Contracted Gaussian-Basis Sets for Molecular Calculations. I. Second Row Atoms, Z = 11-18," *The Journal of Chemical Physics* 72 (1980): 5639–5648, https://doi.org/10.1063/1.438980.

[102] R. Krishnan, J. S. Binkley, R. Seeger, and J. A. Pople, "Self-Consistent Molecular Orbital Methods. XX. A Basis Set for Correlated Wave Functions," *The Journal of Chemical Physics* 72 (1980): 650–654, https://doi.org/10.1063/1.438955.

[103] A. J. H. Wachters, "Gaussian Basis Set for Molecular Wavefunctions Containing Third-Row Atoms," *The Journal of Chemical Physics* 52 (1970): 1033–1036, https://doi.org/10.1063/1.1673095.
[104] P. J. Hay, "Gaussian Basis Sets for Molecular Calculations. The Representation of 3d Orbitals in Transition-Metal Atoms," *The Journal of Chemical Physics* 66 (1977): 4377–4384, https://doi.org/10.1063/1.433731.
[105] K. Raghavachari and G. W. Trucks, "Highly Correlated Systems. Excitation Energies of First Row Transition Metals Sc-Cu," *The Journal of Chemical Physics* 91 (1989): 1062–1065, https://doi.org/10.1063/1.457230.
[106] S. Grimme, J. Antony, S. Ehrlich, and H. Krieg, "A Consistent and Accurate Ab Initio Parametrization of Density Functional Dispersion Correction (DFT-D) for the 94 Elements H-Pu," *The Journal of Chemical Physics* 132 (2010): 154104, https://doi.org/10.1063/1.3382344.
[107] S. Grimme, S. Ehrlich, and L. Goerigk, "Effect of the Damping Function in Dispersion Corrected Density Functional Theory," *Journal of Computational Chemistry* 32 (2011): 1456–1465, https://doi.org/10.1002/jcc.21759.
[108] D. G. A. Smith, L. A. Burns, K. Patkowski, and C. D. Sherrill, "Revised Damping Parameters for the D3 Dispersion Correction to Density Functional Theory," *The Journal of Physical Chemistry Letters* **7** (2016): 2197–2203, https://doi.org/10.1021/acs.jpclett.6b00780.
[109] F. Weigend and R. Ahlrichs, "Balanced Basis Sets of Split Valence, Triple Zeta Valence and Quadruple Zeta Valence Quality for H to Rn: Design and Assessment of Accuracy," *Physical Chemistry Chemical Physics* **7** (2005): 3297–3305, https://doi.org/10.1039/B508541A.
[110] E. Ramos-Cordoba, V. Postils, and P. Salvador, "Oxidation States from Wave Function Analysis," *Journal of Chemical Theory and Computation* 11 (2015): 1501–1508, https://doi.org/10.1021/ct501088v.
[111] P. Salvador and E. Ramos-Cordoba, "Communication: An approximation to Bader's Topological Atom," *The Journal of Chemical Physics* 139 (2013): 071103, https://doi.org/10.1063/1.4818751.
[112] P. Salvador, E. Ramos-Cordoba, M. Montilla, L. Pujal, and M. Gimferrer, "APOST-3D: Chemical Concepts from Wavefunction Analysis," *The Journal of Chemical Physics* 160 (2024): 172502, https://doi.org/10.1063/5.0206187.
[113] G. te Velde, F. M. Bickelhaupt, E. J. Baerends, C. Fonseca Guerra, S. J. van Gisbergen, J. G. Snijders, and T. Ziegler, "Chemistry with ADF," *Journal of Computational Chemistry* 22 (2001): 931–967, https://doi.org/10.1002/jcc.1056.
[114] E. van Lenthe and E. J. Baerends, "Optimized Slater-Type Basis Sets for the Elements 1-118," *Journal of Computational Chemistry* 24 (2003): 1142–1156, https://doi.org/10.1002/jcc.10255.
[115] G. Antinucci, B. Dereli, A. Vittoria, P. H. M. Budzelaar, R. Cipullo, G. P. Goryunov, P. S. Kulyabin, D. V. Uborsky, L. Cavallo, C. Ehm, A. Z. Voskoboynikov, and V. Busico, "Selection of Low-Dimensional 3-D Geometric Descriptors for Accurate Enantioselectivity Prediction," *ACS Catalysis* 12 (2022): 6934–6945, https://doi.org/10.1021/acscatal.2c00976.

[116] T. P. Latendresse, V. Vieru, B. O. Wilkins, N. S. Bhuvanesh, L. F. Chibotaru, and M. Nippe, "Magnetic Properties of a Terbium-[1]Ferrocenophane Complex: Analogies between Lanthanide–Ferrocenophane and Lanthanide–Bis-phthalocyanine Complexes," *Angewandte Chemie International Edition* 57 (2018): 8164–8169; *Angewandte Chemie* 130 (2018) 8296–8301 https://doi.org/10.1002/anie.201804075.

[117] G. R. Whittell, B. M. Partridge, O. C. Presly, C. J. Adams, and I. Manners, "Strained Metallocenophanes with Late Transition Metals in the Bridge: Syntheses and Structures of Nickel- and Platinum-Bridged [1]Ferrocenophanes," *Angewandte Chemie International Edition.* 47 (2008): 4354–4357, https://doi.org/10.1002/anie.200705672.

[118] A. D. Russell, G. R. Whittell, M. F. Haddow, and I. Manners, "Facile Formation of FePd Nanoparticles from Single-Source [1]Ferrocenophane Precursors," *Organometallics* 33 (2014): 5349–5357, https://doi.org/10.1021/om500673r.

[119] I. Matas, G. R. Whittell, B. M. Partridge, J. P. Holland, M. F. Haddow, J. C. Green, and I. Manners, "Synthesis, Electronic Structure, and Reactivity of Strained Nickel-, Palladium-, and Platinum-Bridged [1]Ferrocenophanes," *Journal of the American Chemical Society* 132 (2010): 13279–13289, https://doi.org/10.1021/ja103367e.

[120] R. Broussier, A. Da Rold, B. Gautheron, Y. Dromzee, and Y. Jeannin, "[1]Ferrocenophanes Containing a Group 4 Transition Metal in the Bridge. X-ray Structure of One Representative," *Inorganic Chemistry* 29 (1990): 1817–1822, https://doi.org/10.1021/ic00335a011.

[121] A. Bucaille, T. Le Borgne, M. Ephritikhine, and J.-C. Daran, "Synthesis and X-ray Crystal Structure of a Urana[1]ferrocenophane, the First Tris(1,1‘-ferrocenylene) Metal Compound" *Organometallics* 19 (2000): 4912–4914, https://doi.org/10.1021/om000483f.

[122] T. P. Latendresse, N. S. Bhuvanesh, and M. Nippe, "Slow Magnetic Relaxation in a Lanthanide-[1]Metallocenophane Complex," *Journal of the American Chemical Society* 139 (2017): 8058–8061, https://doi.org/10.1021/jacs.7b01499.

[123] T. P. Latendresse, V. Vieru, A. Upadhyay, N. S. Bhuvanesh, L. F. Chibotaru, and M. Nippe, "Trends in Trigonal Prismatic Ln-[1]Ferrocenophane Complexes and Discovery of a Ho^{3+} Single-Molecule Magnet," *Chemical Science* 11 (2020): 3936–3951, https://doi.org/10.1039/D0SC01197E.

[124] S. Sadeh, G. Schatte, and J. Müller, "A Flexible Approach to Strained Sandwich Compounds: Chiral [1]Ferrocenophanes with Boron, Gallium, Silicon, and Tin in Bridging Positions," *Chemistry – A European Journal* 19 (2013): 13408–13417, https://doi.org/10.1002/chem.201301991.

[125] H. Bhattacharjee, J. D. Martell, E. K. Sarbisheh, S. Sadeh, J. W. Quail, and J. Müller, "Insight into the Formation of Highly Strained [1]Ferrocenophanes with Boron in Bridging Position," *Organometallics* 35 (2016): 2156–2164, https://doi.org/10.1021/acs.organomet.6b00388.

[126] H. Bhattacharjee, J. Zhu, and J. Müller, "Unique Bora[1]ferrocenophanes with Sterically Protected Boron: A Potential Gateway to Helical Polyferrocenes," *Angewandte Chemie. International Edition* 58 (2019): 16575–16582; *Angewandte Chemie* 131 (2019):16728–16735, https://doi.org/10.1002/anie.201908993.

[127] C. L. Lund, J. A. Schachner, J. W. Quail, and J. Müller, "[1]Ferrocenophanes, [1]Chromarenophanes, and [1]Vanadarenophanes with Aluminium and Gallium in Bridging Positions," *Organometallics* 25 (2006): 5817–5823, https://doi.org/10.1021/om060736q.

[128] W. Finckh, B.-Z. Tang, D. A. Foucher, D. B. Zamble, R. Ziembinski, A. Lough, and I. Manners, "The Polymerization Behavior of [1]- and [2]Ferrocenophanes Containing Silicon Atoms in the Bridge: Comparison of the Molecular Structure of the Strained, Polymerizable Cyclic Ferrocenylsilane $Fe(\eta\text{-}C_5H_4)_2(SiMe_2)$ with That of the Cyclic Ferrocenyldisilane $Fe(\eta\text{-}C_5H_4)_2(SiMe_2)_2$," *Organometallics* 12 (1993): 823–829, https://doi.org/10.1021/om00027a035.

[129] J. A. Schachner, J. W. Quail, and J. Müller, "The Galla[1]ferrocenophane {[dimethyl(2-pyridyl)silyl]bis(trimethylsilyl)methyl-$\kappa^2 C,N$}(ferrocene-1,1′-diyl)gallium(III)," *Acta Crystallographica E* 64 (2008): m517, https://doi.org/10.1107/S1600536808005503.

[130] D. A. Foucher, M. Edwards, R. A. Burrow, A. J. Lough, and I. Manners, "Ring-Opening Polymerization of Strained, Ring-Tilted [1]Ferrocenophanes with Germanium in the Bridge: Structures of the [1]Germaferrocenophane $Fe(\eta\text{-}C_5H_4)_2GeMe_2$ and the Ferrocenylgermane $Fe(\eta\text{-}C_5H_4GeEt_2Cl)(\eta\text{-}C_5H_5)$," *Organometallics* 13 (1994): 4959–4966, https://doi.org/10.1021/om00024a044.

[131] M. J. MacLachlan, A. J. Lough, W. E. Geiger, and I. Manners, "Synthesis, Structures, and Properties of Strained Spirocyclic [1]Sila- and [1]Germaferrocenophanes and Tetraferrocenylsilane," *Organometallics* 17 (1998): 1873–1883, https://doi.org/10.1021/om971071i.

[132] S. Zürcher, V. Gramlich, and A. Togni, "Germanium-Containing Ferrocenes and Ferrocenophanes. Potential Precursors for Ring-Opening Polymerizations and 'Germaferrocenes'," *Inorganica Chimica Acta* 291 (1999): 355–364, https://doi.org/10.1016/S0020-1693(99)00133-4.

[133] M. Castruita, F. Cervantes-Lee, J. S. Mahmoud, Y. Zhang, and K. H. Pannell, "Synthesis and Characterization of New Germylferrocenyl, Germylferrocenophane and Polymeric Germyleneferrocenylene Systems," *Journal of Organometallic Chemistry* 637–639 (2001): 664–668, https://doi.org/10.1016/S0022-328X(01)00962-7.

[134] G. Thaler, B. Klotz, K. Wurst, and F. Sladky, "Bis[bis(trimethylsilyl)cyclopentadienyl]-selane as a Ligand: Selena-2,2′,4,4′-tetrakis(trimethylsilyl)-[1]ferrocenophane and 1,2,3-Triselena-2,2′,4,4′-tetrakis(trimethylsilyl)-[3]ferrocenophane," *Journal of Organometallic Chemistry* 637–639 (2001): 745–750, https://doi.org/10.1016/S0022-328X(01)00966-4.

[135] F. Jäkle, R. Rulkens, G. Zech, D. A. Foucher, A. J. Lough, and I. Manners, "Synthesis, Reactivity, and Ring-Opening Polymerization (ROP) of Tin-Bridged [1]Ferrocenophanes," *Chemistry – A European Journal* 4 (1998): 2117–2128, https://doi.org/10.1002/(SICI)1521-3765(19981102)4:11<2117::AID-CHEM2117>3.0.CO;2-E.

[136] H. K. Sharma, F. Cervantes-Lee, J. S. Mahmoud, and K. H. Pannell, "(Bis{2,4,6-triisopropylphenyl}stannylene)ferrocenophane and Related Ring-Opened Products," *Organometallics* 18 (1999): 399–403, https://doi.org/10.1021/om9805234.
